# The polymorphism analysis and therapy vaccine target epitopes screening of HPV-35 E6 E7 among the threaten α-9 HPV in Sichuan area

**DOI:** 10.1186/s12985-024-02357-3

**Published:** 2024-09-09

**Authors:** Jiaoyu He, Tianjun Li, Chunlan Cheng, Ning Li, Peng Gao, Dan Lei, Rong Liang, Xianping Ding

**Affiliations:** 1 Chengdu Second People’s Hospital, Chengdu, Sichuan 610021 China; 2 Key Laboratory of Bio-Resources and Eco-Environment of Ministry of Education, College of Life Sciences, Sichuan University, Chengdu, 610065 China; 3 Department of Laboratory Medicine, Sichuan Provincial Maternity and Child Health Care Hospital. The Affiliated Women’s and children’s Hospital of Chengdu Medical College, Chengdu, China; 4 Department of Ultrasound, Sichuan Provincial Maternity and Child Health Care Hospital, Chengdu, China; 5 Chongqing Nanchuan biotechnology research institute, Bio-resource Research and Utilization Joint Key Laboratory of Sichuan and Chongqing, Sichuan and Chongqing, China; 6 West China Hospital of Sichuan University, 610041, Chengdu, China

**Keywords:** Human Papillomavirus, α-9 genus HPV, HPV35, *E6* polymorphisms, *E7* polymorphisms, Protein structure, Positive selection site, Antigen epitope

## Abstract

High-risk human papilloma virus (HR-HPV) persistent infection is closely associated with the development of cervical cancer and squamous intraepithelial lesion (SIL).The α-9 HPVs, which is predominantly composed of HR-HPV types, account for 75% of HR-HPV infection in Sichuan. The oncoproteins E6 and E7 of HPV play a crucial role in tumor initiation and progression. Notably, HPV-35 is the only HR-HPV type within the α-9 genus that is not included in the nine-valent HPV prophylactic vaccine. Cervical cell samples obtained from Sichuan were collected for HPV detection and genotyping. Among the 406 HPV-positive samples, 31 HPV-35 were detected, 24 HPV-35 E6 and 26 E7 were successfully amplified and sequenced, five nucleotide mutations in E6 and three in E7 were detected, T232C, T434G of E6 (W78R, I145R) and C67T, G84T of E7 (H23Y, L28F) were non-synonymy mutation. PAML 4.8 server was used to detect positive selection sites of HPV-35 E6, E7, and E6 is W78R. Phyre2 were used to predict and analyze protein structures, W78R made influences on protein structure. IEDB were used to screen epitopes vaccine target for HPV-35 affection therapy, and 5 HPV-35 E6 and 3 HPV-35 E7 most potential epitopes were obtained, the most potential peptides for therapy vaccine design were 79-91YRYSVYGETLEKQ, 45-60FACYDLCIVREGQPY, 124-135RFHNIGGRWTGR of E6; 3-19GEITTLQDYVLDLEPEA, 38-47TIDGPAGQAK, 70-88VQSTHIDIRKLEDLLMGTF of E7 and W78R mainly decreased the epitopes affinity.

**Conclusions** Amino acid substitution in the positive selection sites of HPV-35 E6 and E7 genes have been found to influence protein structure and to decrease the overall affinity of antigen epitopes. This observation aligns with the evolutionary significance of positive selection site, which may confer advantages to the virus by making infected cells more challenging for the immune system to detect, thereby enhancing HPV’s adaptability to the host environment. The polymorphism analysis of HPV-35 E6, E7 contributes to the enrichment of α-9 HPV data in Sichuan China, which is instrumental in improving the effectiveness of clinical detection. Furthermore, these findings provide a relevant theoretical foundation for the prevention and treatment of HPV-related diseases.

**Supplementary Information:**

The online version contains supplementary material available at 10.1186/s12985-024-02357-3.

## Introduction

Cervical cancer, alongside liver cancer, is a type of malignancy associated with viral infection with 99.7% of cases linked to persistent infection with HR-HPVs. The α-9 genus HPVs predominantly comprises mucosal HR-HPVs, representing approximately 75% of HR-HPV infection in Sichuan. This genus includes HPV types 16, 31, 33, 35, 52 and 58. Notably, HPV-35 is the only HR α-9 HPV that has been omitted from the nine-valent prophylactic vaccine and has not been extensively studied. The scarcity of data on HPV-35 hinders our understanding of its characteristics. Since HPV characteristic can vary by geography and ethnicity, it is particularly crucial to conduct research in Sichuan, to provide a theoretical basis for the prevention and treatment of HPV-35 infections.

HR-HPV are known to induce the occurrence and progression of cervical cancer and other malignant tumors primarily through the action of their E6 and E7 oncoproteins [1]. Protein structure determines its function, HPV E6 is consists of one N-terminal (residues 1–36), one C-terminal (residues 147–158) and two Zinc finger (residues 37–73 and 110–146). These Zinc finger motifs are characterized by a consensus CXXC-(29x)-CXXC amino acid sequence and are critical for binding zinc ions, which are essential for various cellular processes, including RNA transcription, transformation, cell immortalization, and protein–protein interactions with host cell proteins. Moreover, the two Zinc finger domains fold into a deep pocket, which, through their common alpha-helical structure, is involved in the ubiquitination and degradation of the tumor suppressor protein p53 [2–7]. Distinct from low-risk HPVs (LR-HPVs), the carboxyl terminal of HR-HPV E6 contains a highly conserved and unique amino acid sequence that serves as a PDZ-binding motif. This motif is known to interact with PDZ domain-containing proteins, which are involved in intracellular cell adhesion, cell junction formation and cell polarization. The binding of E6 to these specific proteins can confer a more aggressive phenotype on cells or maintain high levels of protein expression [8, 9]. The PDZ-binding motif (residues 145–149) is a key target for the E6 protein’s involvement in cellular transformation, and the carboxy terminal half, principally responsible for p53 binding, appears to be crucial for the induction of cellular immortality [10]. HPV E7, on the other hand, promotes cell immortalization and inhibits apoptosis in infected cells by binding to the retinoblastoma protein (pRB). The E7 protein is divided into three conserved regions (CR1:1–15, CR2:16–37, CR3:38–98). The CR2 region contains a strictly conserved sequence LXCXE, with amino acids at positions 21–29 comprising the pRB-binding regions [11]. The CR2 region exhibits a high affinity for the RB protein and considered the active region of the E7 protein. Additionally, the LXCXE sequence serves as a phosphorylation site for casein kinase (CK) II [12], further modulating the protein’s interactions and functions within the host cell. The CR3 region of HPV E7 contains two CXXC sequences separated by 29 and 30 amino acids, respectively, which constitute the zinc finger binding domain. The amino acids at positions 58, 61, 91 and 94 of the HPV E7 protein serve as the Zn-binding sites. The zinc finger binding domain is involved in the interaction with a variety of functional proteins [13]. Substitutions within this region have the potential to alter the conformation, and consequently, the function of the E7 protein to some degree.

Human leukocyte antigens (HLAs) are molecules presen on the surface of all human cells that plays an crucial role in distinguishing immune responses by recognizing autoantigen epitope targets and either stimulating or modulating these responses. This mechanism enables the body to resist and eliminate HPV infections. Currently, there is no specific drug available to cure HPV infection, instead, the primary clearance mechanism is through the immune system, with T cell immunity being the key component. In the context of HPV infection-related immune responses, CD8^+^ cytotoxic T lymphocytes (CTL) and CD4^+^ helper T lymphocytes (Th) cells are the primary functional cells. The activation and proliferation of CTLs can directly kill tumor cells and secrete cytokines to combat tumors, making them the primary effectors against HPV infection and cervical cancer cells [14, 15]. Activated CD4^+^ Th cells can produce lymphocytokines that enhance the function of CTLs and natural killer cells (NK cells), activate antigen-presenting cells (APCs), and assist in the elimination of HPV-infected and cervical cancer cells [16]. The E6 and E7 proteins are early proteins, distinct from the targets of preventive vaccines such as L1 and L2, which are expressed in large quantities following infection to activate the immune response. These oncoproteins are considered as important targets for therapeutic vaccines. T-cell epitopes of E6 and E7 have been found to be located in the C-segment and zinc finger region, and HLA-A epitopes may also be present in the N-terminal.

The E6 and E7 oncoproteins of HR-HPVs are critical for the induction of cervical cancer. These proteins not only possess transforming and carcinogenic capabilities but also exhibit trans-activation of viral genes and cellular gene transcription. Upon HPV infection, the immune system recognizes, processes, and presents foreign proteins. Nucleotide non-synonymy mutations can lead to amino acid substitutions in the protein sequence, altering the physical, chemical properties, structure, and immunogenicity of the protein. These changes can affect the immune response to the virus and its pathogenicity. Positive selective mutation can result in a stable increase in gene frequency, enhancing the adaptability of the virus to the environment. This adaptability can lead to changes in infection rates, allowing those variants that are better suited to the natural environment to survive and thrive. Such as specific mutations L83V (L90V), D25E (D32E) in HPV-16 E6 [17]. This adaptability can lead to changes in infection rates, allowing those variants that are better suited to the natural environment to survive and thrive.

Epidemic data indicate that HPV-35 is one of the most prevalent genotypes in cervical cancer cases and is associated with a risk of cervical cancer that is second only to that of HPV-16, making it a significant HR-HPV type. While, there is relevant research data on HPV-35 in North America, Canada, and other places, but lack of relevant research on HPV-35 in China, especially in Sichuan. The types of HPV and the prevalence of specific mutations can vary between different regions and ethnic groups. Consequently, it is imperative to investigate the polymorphism of E6 and E7, positive selection sites, epitopes, and protein structures of HPV-35 in Sichuan. Such research will aid in identifying therapeutic vaccine targets and provide crucial data for the prevention and control of HPV-35 infection-related diseases within this specific region.

## Materials and methods

### Samples resource

Cervical cell specimens were randomly collected from January 2019 to December 2022 at Sichuan Provincial Maternity and Child Health Care Hospital, the Affiliated Hospital of the Sichuan Reproductive Health Research Center, Chengdu Jinsha hospital, the Infertility Hospital Affiliated to Chengdu Medical College, Chengdu Song zi niao Sterility Hospital. These specimens were stored in a -20℃ antiseptic buffer. Prior to sample collection, written informed consent was obtained from all patients or their legal guardians. The study was ethically approved by the Medical Ethics Committee of the Sichuan Provincial Maternity and Child Health Care Hospital, and stringent measures were implemented to ensure patient privacy and confidentiality.

### Genomic DNA extraction and HPV typing

HPV DNA was extracted and genotyped by Human Papillomavirus Genotyping test Kit (Hailes Bio, Ningbo, China), in accordance with the manufacturer’s instructions.

### PCR amplification

Primers specific to the HPV-35 E6, E7 gene regions were designed by PRIMER version 5.0 software and validated through the NCBI (National Center for Biotechnology Information) Primer-Blast tool, based on the reference sequences of HPV-35 (GenBank No: HQ537730.1). These primers were subsequently synthesized by TSINGKE (Chengdu, China). The primer sequences and the polymerase chain reaction (PCR) reaction mix composition are detailed in Supplementary Table S1. The PCR products were visualized by gel electrophoresis in a 2% agarose gel (Sangon Biotech Co., Ltd.), and purified PCR products were subjected to bidirectional DNA sequencing by TSINGKE at least twice (Chengdu, China) to ensure accuracy.

### HPV-35 E6, E7 polymorphisms, evolutionary and positive selection sites analysis

Nucleotide mutation and the corresponding amino-substitution of sequenced HPV-35 E6 and E7 in Sichuan were detected and analyzed by NCBI BLAST, Primer Premier 5 and DNAMAN5.2.2 (Table 1), difference in protein structure were predicted by PSIPred (http://bioinf.cs.ucl.ac.uk/psipred/).

**Table 1:** The polymorphism and protein function effect analysis of HPV-35 E6 and E7

		HPV35 E6					HPV35 E7		
Location		18	27	232	261	434	67	84	114
REF		T	T	T	G	T	C	G	C
Mutation		C	C	C	C	G	T	T	T
No./Frequency		22	22	22	9	23	5	3	22
Substitution		A	P	W78R	T	I145R	H23Y	L28F	T
Structure		C	C	C	α-helix	C	C	C	C
PolyPhen-2		/	/	benign		benign	benign	benign	/
PROVEAN		/	/	Neutral		Neutral	Neutral	Deleterious	/
I-Mutant 2.0	Stability	/	/	Decrease		Decrease	Increase	Decrease	/
	RI	/	/	8	8		4	5	/
	ΔΔG	2.3	-0.9	-1.9	-0.6	1.7	1.8	-1.1	0
RNA structure	Window	1–218	1–227	32–432	61–450	234–450	1–267	1–284	1–300
	Region	1–50	9–58	190–340	235–320	398–447	55–180	59–108	102–153
	Distance	0.0495	0.1014	0.0527	0.0162	0.0128	0.0406	0.0811	0.0059
	*P*-value	0.3392	0.1894	0.4029	0.7178	0.6848	0.5424	0.3055	0.8847

REF means nucleotide of this site in the reference sequence; Stability changes upon mutation from the protein sequence or structure; Window means Folding Window; Region means Local region; Substitution means Amino substitution

Evolutionary analysis of HPV-35 E6, E7 genes were conducted by MEGA 6.0, based on the reference sequence of HPV-35 E6 and E7 (GenBank No.: HQ537730.1-HQ537708.1) [18]. The positive selection sites of HPV-35 E6 and E7 were conducted by Maximum Likelihood 4.8 (PAML 4.8, http://abacus.gene.ucl.ac.uk/software/paml.html) through ù = dN/dS ratio calculation (dN: non-synonymous mutation rate, dS: synonymous mutation rate) [19].

### The effect of HPV-35 E6, E7 polymorphism on RNA, protein structure and function

The impact of non-synonymous mutation on protein function was assessed by the Polymorphism Phenotyping v2 (PolyPhen-2, http://genetics.bwh.harvard.edu/pph2) and the Protein Variation Effect Analyzer (PROVEAN, http://provean.jcvi.org) Online software [20, 21].

The impact of non-synonymous mutation on RNA splicing pattern and structure were investigated by Alternative Splice Site Predictor (ASSP) Online software (http://wangcomputing.com/assp/) and RNAsnp software (http://rth.dk/resources/rnasnp/) respectively [22, 23].

The influence of non-synonymous mutation on protein secondary structure was predicted by Phyre2 (http://www.sbg.bio.ic.ac.uk/~phyre2/html/page.cgi?id=index) [24]. Additionally, the impact of these non-synonymous mutation on protein hydrophobicity and stability was evaluated by ExPASy-ProtScale (https://web.expasy.org/protscale) and I-Mutant 2.0 (http://folding.biofold.org/i-mutant/i-mutant2.0.html) respectively [25, 26].

### HPV-35 E6, E7 optimal T-cell antigen epitopes selection

Based on the major histocompatibility complex database (dbMHC), HLA alleles with an average frequency of over 5% in the Chinese population were designated as high frequency allele for the selection of T-cell epitopes in Sichuan. This selection was performed by the Immunology Database and Analysis Project (IEDB) resources (http://www.iedb.org/) (Supplementary Table S1). For potential vaccine design targets, epitopes with lower peptides percentile rank (PR) were considered to have better affinity. Therefore, HLA-I epitopes with PR < 1.0 and HLA-II epitopes with PR < 5.0 were chosen for potential vaccine design target [27].

## Results

### HPV-35 prevalence and E6, E7 polymorphisms in Sichuan

Among the 406 HPV positive cell specimens, 31 HPV-35 positive samples were detected (7.63%), among that 24 E6 and 26 E7 of HPV-35 were succeed sequenced. Compare with HPV-35 reference sequence (HQ533730.1), all E6 and 24 E7 were variants, 5 nucleotide mutations were detected in E6 sequence and 3 in E7, details were shown in Table 1. In E6, T232C, T434G non-synonymous mutations made W78R and I145R, and in E7, C67T, G84T made H23Y and L28F respectively.

### Evolutionary and positive selection sites analysis of HPV-35 E6, E7

**Fig.1:**
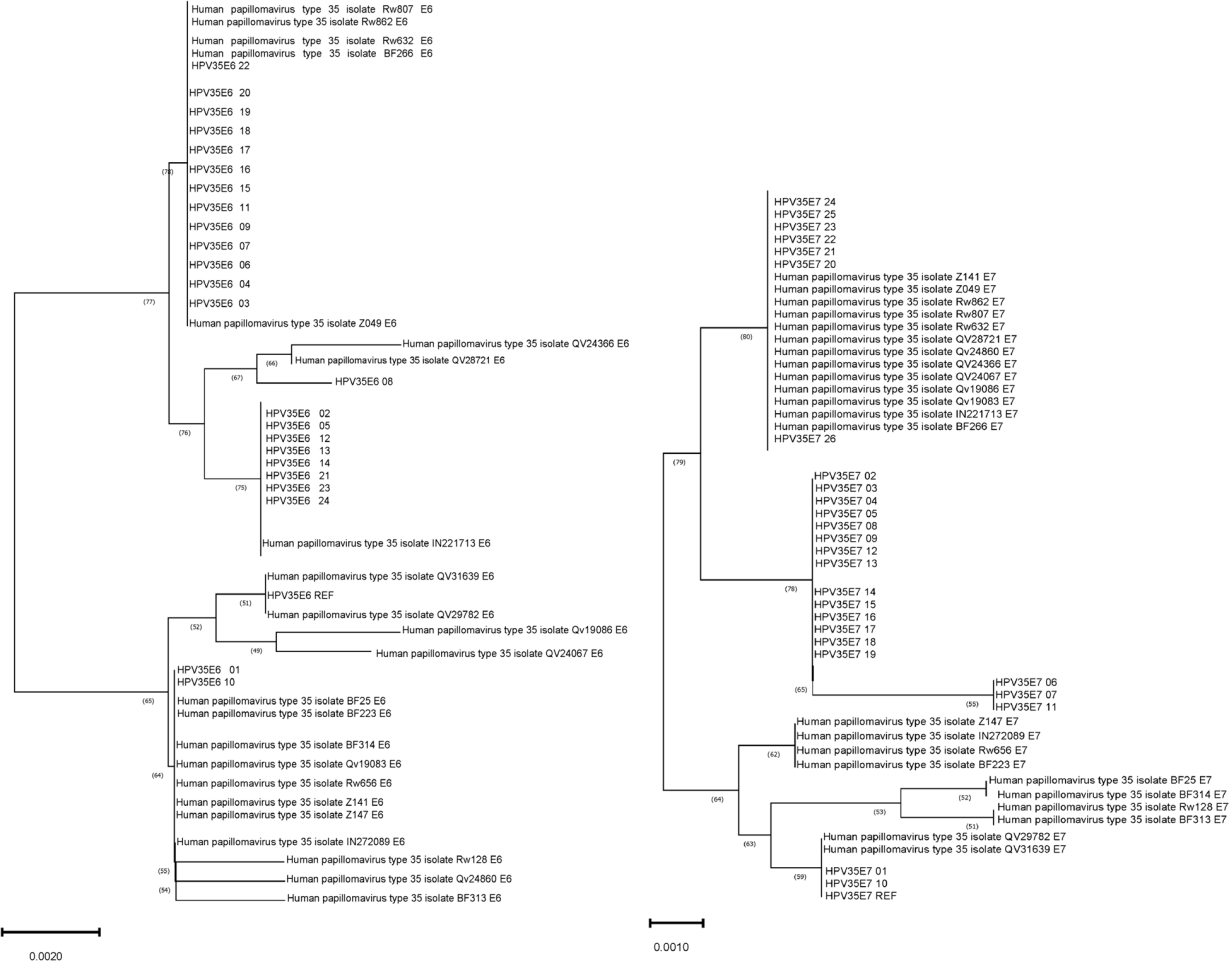
Evolutionary tree of HPV-35 E6, E7

The phylogenetic tree constructed based on the HPV-35 sample sequences, reference sequences and known sequences is presented in Fig. 1. In this study, no novel mutation emerged, and all sequenced mutant strains were identified. Notably, the sample sequences QV31639 and QV29782 exhibited the highest homology to the reference sequence. Similarly, the sequences HPV35E6/E7 01 and HPV-35 E6/E7 10 demonstrated the highest homology to the reference sequence, suggesting that these mutant strains may have originated from reference sequences. The HPV-35 A1 and A2 line variants previously identified by Chen Zigui et al. were not included in this analysis. However, the HPV E610 and HPV E620 sample were sequenced and are numbered in Fig. 1. These sequences have been deposited in Genbank under the names: HPV-35 E6 01- HPV-35 E6 24, HPV-35 E7 01- HPV-35 E7 24.

Calculated by Codeml software using Naive NEB and Bayes Empirical Bayes models, the positive selection site of HPV-35 E6 was W78R.

### Non-synonymous mutation effects on alternative splice site, structure of HPV-35 E6, E7 RNA

In the HPV-35 E6 gene, the nucleotide mutation T232C (W78R) made a different in Intron GC, Alt./Cryptic, Constitutive, Confidence of sequence splice site in 220 bp, Score, Alt./Cryptic, Constitutive, Confidence of sequence splice site in 233 bp, 244 bp. T434G (I145R) made a different in Alt./Cryptic, Constitutive, Confidence of sequence splice site in 220 bp, Constitutive, Confidence of sequence splice site in 233 bp, T232C and T434G indicated that c.T272G mutation influences the RNA splice site (Table 2). In HPV-35 E7, G84T (L28F) made 2 new sequence splice sites in 93 bp and 98 bp.

**Table 2:** Effect of non-synonymous mutation on RNA splicing pattern

Type	Position (bp)	Putative splice site	Sequence	Score^a^	Activations^b^			
					Intron GC^a^	Alt./Cryptic	Constitutive	Confidence^b^
HPV 35 E6 REF	54	Alt. isoform/cryptic donor	GTGCAACGAGgtagaagaaa	6.567	0.371	0.725	0.210	0.711
	118	Alt. isoform/cryptic donor	TTACAGCGGAgtgaggtata	5.981	0.386	0.700	0.239	0.658
	123	Alt. isoform/cryptic donor	GCGGAGTGAGgtatatgact	7.619	0.371	0.721	0.207	0.713
	220	Alt. isoform/cryptic donor	TCAAAAATAAgtgaatatag	5.083	0.314	0.912	0.063	0.931
	233	Alt. isoform/cryptic donor	AATATAGATGgtatagatat	8.326	0.271	0.864	0.099	0.885
	244	Alt. isoform/cryptic donor	TATAGATATAgtgtgtatgg	7.600	0.300	0.929	0.048	0.948
	305	Alt. isoform/cryptic donor	TATTAATTAGgtgtattaca	6.820	0.343	0.921	0.057	0.938
	306	Constitutive acceptor	tattaattagGTGTATTACA	4.984	0.271	0.382	0.595	0.358
HPV 35 E6 W78R	54	Alt. isoform/cryptic donor	GTGCAACGAGgtagaagaaa	6.567	0.371	0.725	0.210	0.711
	118	Alt. isoform/cryptic donor	TTACAGCGGAgtgaggtata	5.981	0.386	0.700	0.239	0.658
	123	Alt. isoform/cryptic donor	GCGGAGTGAGgtatatgact	7.619	0.371	0.721	0.207	0.713
	220	Alt. isoform/cryptic donor	TCAAAAATAAgtgaatatag	5.083	0.329	0.920	0.057	0.938
	233	Alt. isoform/cryptic donor	AATATAGACGgtatagatat	7.358	0.271	0.872	0.095	0.891
	244	Alt. isoform/cryptic donor	TATAGATATAgtgtgtatgg	7.682	0.300	0.927	0.049	0.947
	305	Alt. isoform/cryptic donor	TATTAATTAGgtgtattaca	6.820	0.343	0.921	0.057	0.938
	306	Constitutive acceptor	tattaattagGTGTATTACA	4.984	0.271	0.382	0.595	0.358
HPV 35 E6 I145R	54	Alt. isoform/cryptic donor	GTGCAACGAGgtagaagaaa	6.567	0.371	0.725	0.210	0.711
	118	Alt. isoform/cryptic donor	TTACAGCGGAgtgaggtata	5.981	0.386	0.700	0.239	0.658
	123	Alt. isoform/cryptic donor	GCGGAGTGAGgtatatgact	7.619	0.371	0.721	0.207	0.713
	220	Alt. isoform/cryptic donor	TCAAAAATAAgtgaatatag	5.083	0.314	0.903	0.070	0.923
	233	Alt. isoform/cryptic donor	AATATAGATGgtatagatat	8.326	0.271	0.847	0.112	0.868
	244	Alt. isoform/cryptic donor	TATAGATATAgtgtgtatgg	7.600	0.300	0.922	0.052	0.943
	305	Alt. isoform/cryptic donor	TATTAATTAGgtgtattaca	6.820	0.343	0.921	0.057	0.938
	306	Constitutive acceptor	tattaattagGTGTATTACA	4.984	0.271	0.382	0.595	0.358
HPV 35 E7 REF	184	Alt. isoform/cryptic donor	TGTTGTAAATgtgaggcgac	7.053	0.400	0.947	0.036	0.962
	203	Alt. isoform/cryptic donor	CACTACGTCTgtgtgtacag	6.532	0.371	0.801	0.146	0.817
	214	Alt. isoform/cryptic acceptor	gtgtgtacagAGCACACACA	4.825	0.414	0.715	0.272	0.620
HPV 35 E7 H23Y	184	Alt. isoform/cryptic donor	TGTTGTAAATgtgaggcgac	7.053	0.400	0.947	0.036	0.962
	203	Alt. isoform/cryptic donor	CACTACGTCTgtgtgtacag	6.532	0.371	0.801	0.146	0.817
	214	Alt. isoform/cryptic acceptor	gtgtgtacagAGCACACACA	4.825	0.414	0.715	0.272	0.620
HPV 35 E7 L28F	93	Alt. isoform/cryptic acceptor	tttgtgacagCTCAGAGGAG	2.353	0.414	0.820	0.169	0.793
	98	Alt. isoform/cryptic acceptor	gacagctcagAGGAGGAGGA	2.467	0.429	0.882	0.113	0.872
	184	Alt. isoform/cryptic donor	TGTTGTAAATgtgaggcgac	7.053	0.400	0.947	0.036	0.962
	203	Alt. isoform/cryptic donor	CACTACGTCTgtgtgtacag	6.532	0.371	0.801	0.146	0.817
	214	Alt. isoform/cryptic acceptor	gtgtgtacagAGCACACACA	4.825	0.414	0.715	0.272	0.620

^a^Scores of the preprocessing models reflecting splice site strength, i.e. a PSSM for putative acceptor sites, and an MDD model for putative donor sites. Intron GC values correspond to 70 nt of the neighboring intron

^b^Activations are output values of the backpropagation networks used for classification. High values for one class with low values of the other class imply a good classification. Confidence is a simple measure expressing the differences between output activations. Confidence ranges between zero (undecided) to one (perfect classification)

By RNAsnp web server, the wild-type, mutant T232C (distance: 0.0527, *p*-value = 0.4029, 190 to 340 nt highlighted), mutant T434G (distance: 0.0128, *p*-value = 0.6848, 398 to 447 nt highlighted) are difference in HPV E6 RNA structure, c.T232C (p.W78R) decreased the minimum free energy of RNA from -80.20 kcal/mol kcal/mol to -82.1 kcal/mol; c.U434G (p.W78R) increased the minimum free energy of RNA from -40.20 kcal/mol to -38.50 kcal/mol, T/C and T/G transition results in the RNA secondary structure change (Fig. 1)

**Fig. 2:**
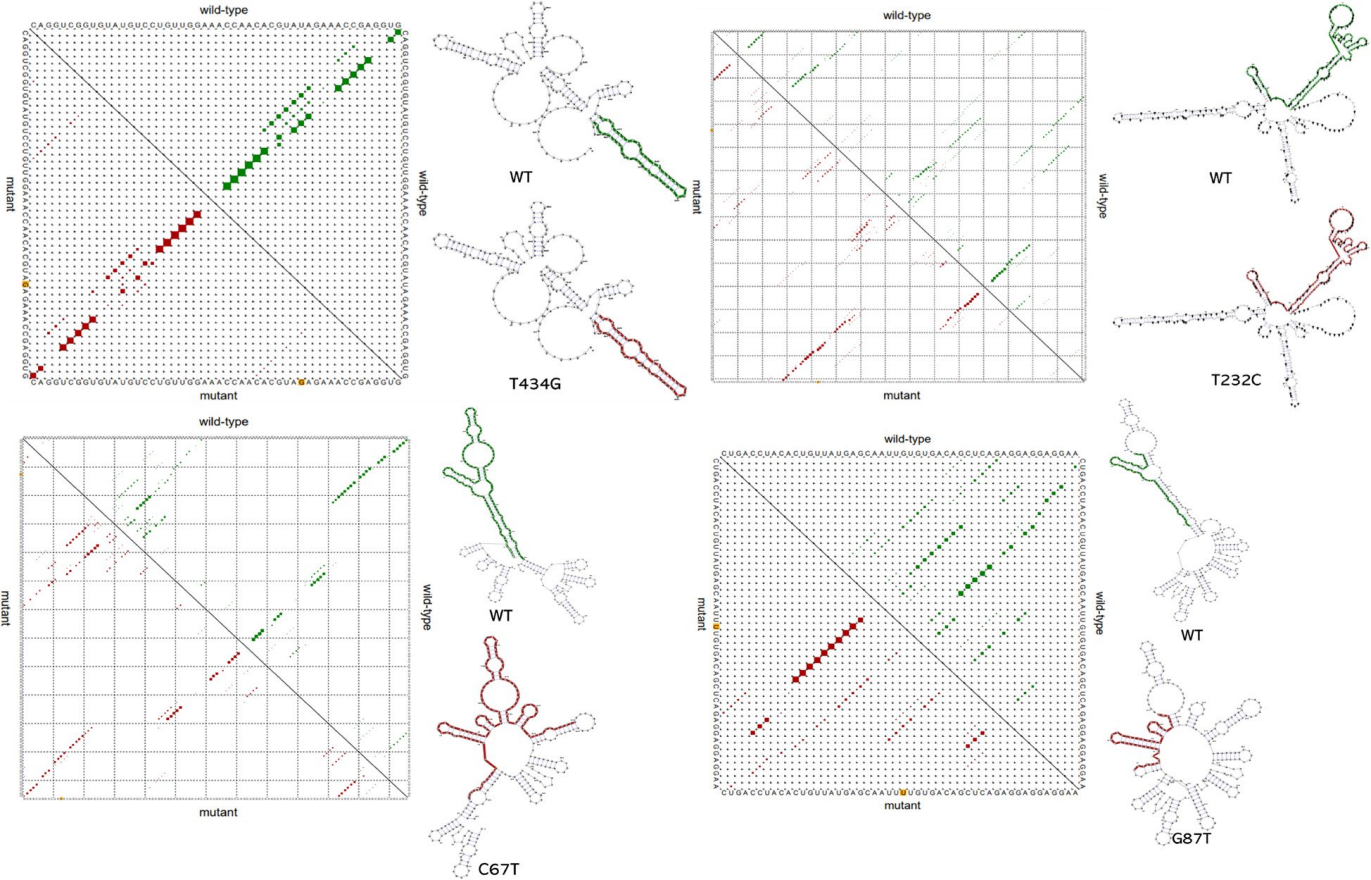
The effect of HPV-35 E6, E7 polymorphism on RNA structure

In the HPV E7 gene, the wild-type, mutant c.C67T (distance: 0.0406, *p*-value = 0.5424, 55 to 180 nt highlighted), mutant G84U (distance: 0.0811, *p*-value = 0.3055, 59 to 108 nt highlighted) are difference in RNA structure, c.C67T (p.H23Y) increased the minimum free energy of RNA from -58.40 kcal/mol to -56.60 kcal/mol; c.G84T (p.L28F) decreased the minimum free energy of RNA from -40.20 kcal/mol to -38.50 kcal/ mol, the T/C and G/T transition results in the RNA secondary structure change (Fig. 2).

### The effects of non-synonymous mutation on HPV-35 E6, E7 protein structure, structural stability, and hydrophilicity

**Fig. 3:**
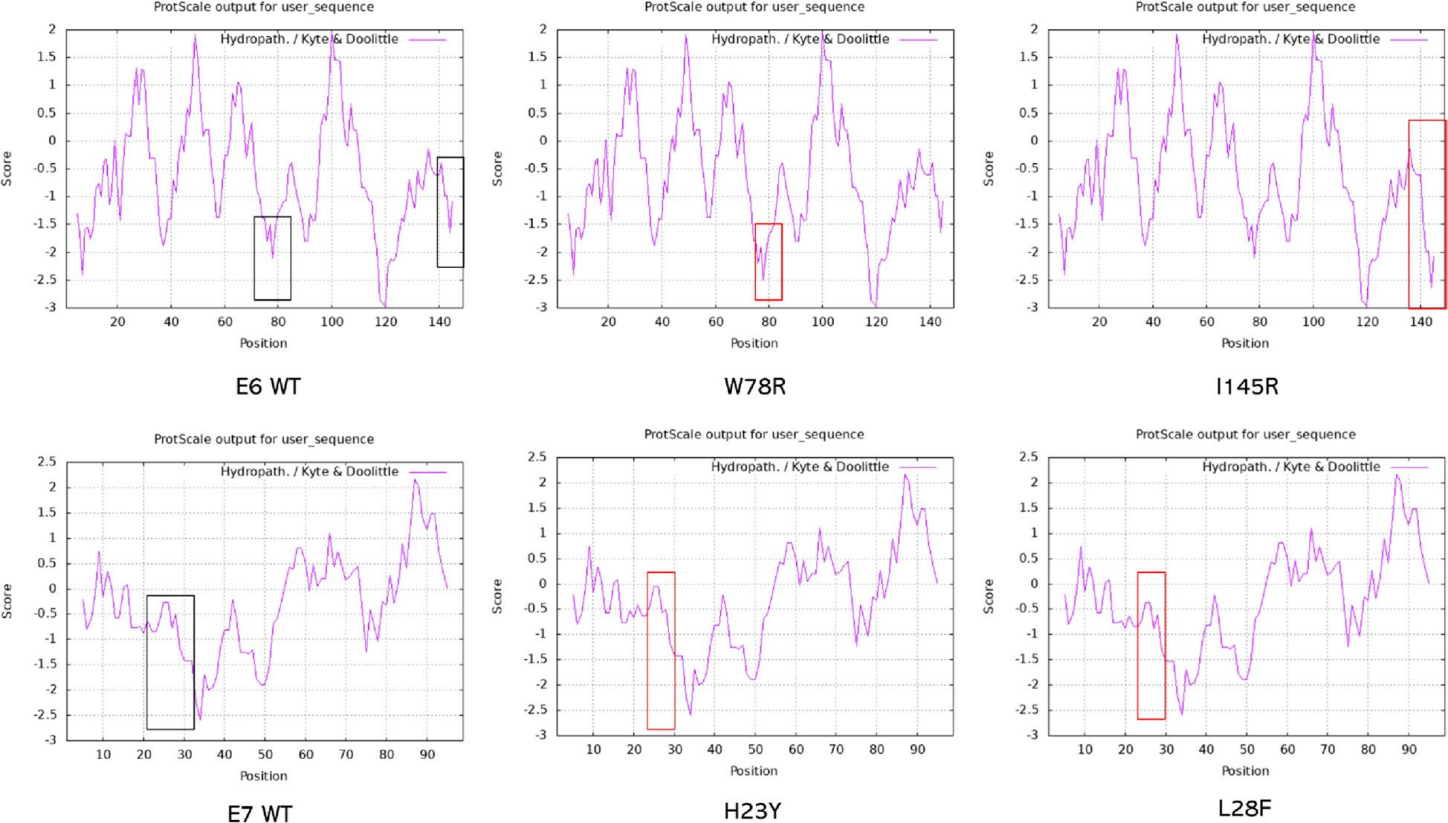
The effect of non-synonymous mutation on HPV-35 E6, E7 hydrophobicity

In the E6 protein, the mutations W78R, I145R were not situated within the alpha-helix or beta-sheet. The W78R resulted in an increase in the lowest free energy of the mutant protein increased from -80.20 to -82.10 kcal/mol, indicating enhanced hydrophilicity, but decreased protein structural stability. Similarly, the mutation I145R led to an increase in the lowest free energy from -40.20 to -38.50 kcal/mol, again suggesting enhanced hydrophilicity and reduced protein structural stability (Fig. 3 and Table 1).

In the E7 protein, the mutation H23Y caused the disappearance of the alpha-helix located between the 15th and 89th amino acids, which is remote from the site of substitution. This mutation increased the lowest free energy of the mutant protein from -58.40 to -56.60 kcal/mol, indicating decreased hydrophilicity and increased protein structural stability. Conversely, the mutation L28F resulted in a decrease in the lowest free energy of the mutant protein from -70.00 to -71.10 kcal/mol, suggesting enhanced hydrophilicity and decreased protein structural stability (Fig. 3 and Table 1).

### T-cell antigen epitopes of HPV-35 E6, E7

In HPV-35 E6 reference sequence, 92 HLA-I and 84 HLA-II epitopes were predicted. In W78R variant, 90 HLA-I and 89 HLA-II epitopes were predicted, W78R made 5 HLA-I, 19 HLA-II new epitopes appear, 7 HLA-I, 14 HLA-II epitopes disappear, and 5 HLA-I, 41 HLA-II epitopes affinity increase; amino-acid substitution made HLA-Iepitopes affinity, number decrease and HLA-II epitopes affinity, number increase. In I145R variant, 93 HLA-I and 84 HLA-II epitopes were predicted, I145R made a new HLA-I epitopes appear. Details were shown in Tables 3 and 4.

**Table 3:** The effect of HPV-35 E6, E7 polymorphism on HLA-I epitopes

HPV35 E6 WT				HPV35 E6 W78R				HPV35 E6 I145R			
Allele	Length	Peptide	Percentile rank	Allele	Length	Peptide	Percentile rank	Allele	Length	Peptide	Percentile rank
HLA-B*58:01	70–78	YSKISEYRW	0.01	HLA-A*33:03	70–78	YSKISEYRR	0.17	HLA-A*33:03	137–145	MSCWKPTRR	0.2
HLA-A*33:03	72–80	KISEYRWYR	0.05	HLA-A*11:01	78–90	RYRYSVYGETLEK	0.19				
HLA-A*24:02	69–78	FYSKISEYRW	0.12	HLA-A*33:03	72–80	KISEYRRYR	0.25				
HLA-A*33:03	71–80	SKISEYRWYR	0.21	HLA-A*33:03	69–78	FYSKISEYRR	0.29				
HLA-B*58:01	69–78	FYSKISEYRW	0.23	HLA-A*11:01	77–90	RRYRYSVYGETLEK	0.48				
HLA-B*58:01	68–78	KFYSKISEYRW	0.27	HLA-A*24:02	78–88	RYRYSVYGETL	0.61				
HLA-A*11:01	72–80	KISEYRWYR	0.28	HLA-A*33:03	71–80	SKISEYRRYR	0.63				
HLA-A*33:03	70–80	YSKISEYRWYR	0.29	HLA-A*33:03	70–80	YSKISEYRRYR	0.68				
HLA-A*24:02	68–78	KFYSKISEYRW	0.35	HLA-A*33:03	68–78	KFYSKISEYRR	0.71				
HLA-A*11:01	77–90	RWYRYSVYGETLEK	0.48	HLA-A*11:01	72–80	KISEYRRYR	0.87				
HLA-A*33:03	69–80	FYSKISEYRWYR	0.63	HLA-A*33:03	69–80	FYSKISEYRRYR	0.98				
HLA-B*58:01	71–78	SKISEYRW	0.83								
HLA-B*58:01	67–78	LKFYSKISEYRW	0.86								
HPV35 E7 WT				HPV35 E7 H23Y				HPV35 E7 L28F			
Allele	Length	Peptide	Percentile rank	Allele	Length	Peptide	Percentile rank	Allele	Length	Peptide	Percentile rank
				HLA-B*15:02	14–23	DLEPEATDLY	0.85				

Note : * is one of the constituent symbols of alleles

**Table 4:** The effect of HPV-35 E6, E7 polymorphism on HLA-II epitopes

HPV35 E6 WT							HPV35 E6 W78R					
Allele	Length		Core peptide	Peptide		Score	Allele		Length	Core peptide	Peptide	score
HLA-DPA1*01:03/DPB1*04:01	77–88		YRYSVYGET	RWYRYSVYGETL		0.42	HLA-DPA1*01:03/DPB1*04:01		77–88	YRYSVYGET	RRYRYSVYGETL	0.18
HLA-DPA1*01:03/DPB1*04:01	76–87		YRYSVYGET	YRWYRYSVYGET		0.42	HLA-DPA1*01:03/DPB1*04:01		76–87	YRYSVYGET	YRRYRYSVYGET	0.18
HLA-DPA1*01:03/DPB1*04:01	76–89		YRYSVYGET	YRWYRYSVYGETLE		0.45	HLA-DPA1*01:03/DPB1*04:01		76–89	YRYSVYGET	YRRYRYSVYGETLE	0.26
HLA-DPA1*01:03/DPB1*04:01	76–88		YRYSVYGET	YRWYRYSVYGETL		0.48	HLA-DPA1*01:03/DPB1*04:01		76–88	YRYSVYGET	YRRYRYSVYGETL	0.23
HLA-DPA1*01:03/DPB1*04:01	77–89		YRYSVYGET	RWYRYSVYGETLE		0.49	HLA-DPA1*01:03/DPB1*04:01		77–89	YRYSVYGET	RRYRYSVYGETLE	0.24
HLA-DPA1*01:03/DPB1*04:01	77–90		YRYSVYGET	RWYRYSVYGETLEK		0.5	HLA-DPA1*01:03/DPB1*04:01		77–90	YRYSVYGET	RRYRYSVYGETLEK	0.28
HLA-DPA1*01:03/DPB1*04:01	76–90		YRYSVYGET	YRWYRYSVYGETLEK		0.5	HLA-DPA1*01:03/DPB1*04:01		76–90	YRYSVYGET	YRRYRYSVYGETLEK	0.23
HLA-DPA1*01:03/DPB1*04:01	75–90		YRYSVYGET	EYRWYRYSVYGETLEK		0.55	HLA-DPA1*01:03/DPB1*04:01		75–90	YRYSVYGET	EYRRYRYSVYGETLEK	0.19
HLA-DPA1*01:03/DPB1*04:01	74–90		YRYSVYGET	SEYRWYRYSVYGETLEK		0.64	HLA-DPA1*01:03/DPB1*04:01		74–90	YRYSVYGET	SEYRRYRYSVYGETLEK	0.32
HLA-DPA1*01:03/DPB1*04:01	75–89		YRYSVYGET	EYRWYRYSVYGETLE		0.76	HLA-DPA1*01:03/DPB1*04:01		75–89	YRYSVYGET	EYRRYRYSVYGETLE	0.38
HLA-DPA1*01:03/DPB1*04:01	73–90		YRYSVYGET	ISEYRWYRYSVYGETLEK		0.78	HLA-DPA1*01:03/DPB1*04:01		73–90	YRYSVYGET	ISEYRRYRYSVYGETLEK	0.33
HLA-DPA1*01:03/DPB1*04:01	76–91		YRYSVYGET	YRWYRYSVYGETLEKQ		0.81	HLA-DPA1*01:03/DPB1*04:01		76–91	YRYSVYGET	YRRYRYSVYGETLEKQ	0.33
HLA-DPA1*01:03/DPB1*04:01	75–91		YRYSVYGET	EYRWYRYSVYGETLEKQ		0.84	HLA-DPA1*01:03/DPB1*04:01		75–91	YRYSVYGET	EYRRYRYSVYGETLEKQ	0.36
HLA-DPA1*01:03/DPB1*04:01	74–91		YRYSVYGET	SEYRWYRYSVYGETLEKQ		0.88	HLA-DPA1*01:03/DPB1*04:01		74–91	YRYSVYGET	SEYRRYRYSVYGETLEKQ	0.43
HLA-DPA1*01:03/DPB1*04:01	75–87		YRYSVYGET	EYRWYRYSVYGET		0.89	HLA-DPA1*01:03/DPB1*04:01		75–87	YRYSVYGET	EYRRYRYSVYGET	0.39
HLA-DPA1*01:03/DPB1*04:01	75–88		YRYSVYGET	EYRWYRYSVYGETL		0.94	HLA-DPA1*01:03/DPB1*04:01		75–88	YRYSVYGET	EYRRYRYSVYGETL	0.39
HLA-DPA1*01:03/DPB1*04:01	75–92		YRYSVYGET	EYRWYRYSVYGETLEKQC		1.2	HLA-DPA1*01:03/DPB1*04:01		75–92	YRYSVYGET	EYRRYRYSVYGETLEKQC	0.54
HLA-DPA1*01:03/DPB1*04:01	77–91		YRYSVYGET	RWYRYSVYGETLEKQ		1.2	HLA-DPA1*01:03/DPB1*04:01		77–91	YRYSVYGET	RRYRYSVYGETLEKQ	0.56
HLA-DPA1*01:03/DPB1*04:01	76–92		YRYSVYGET	YRWYRYSVYGETLEKQC		1.2	HLA-DPA1*01:03/DPB1*04:01		76–92	YRYSVYGET	YRRYRYSVYGETLEKQC	0.56
HLA-DPA1*01:03/DPB1*04:01	74–89		YRYSVYGET	SEYRWYRYSVYGETLE		1.2	HLA-DPA1*01:03/DPB1*04:01		74–89	YRYSVYGET	SEYRRYRYSVYGETLE	0.47
HLA-DPA1*01:03/DPB1*04:01	73–89		YRYSVYGET	ISEYRWYRYSVYGETLE		1.4	HLA-DPA1*01:03/DPB1*04:01		73–89	YRYSVYGET	ISEYRRYRYSVYGETLE	0.63
HLA-DPA1*01:03/DPB1*04:01	74–88		YRYSVYGET	SEYRWYRYSVYGETL		1.5	HLA-DPA1*01:03/DPB1*04:01		74–88	YRYSVYGET	SEYRRYRYSVYGETL	0.74
HLA-DPA1*01:03/DPB1*04:01	78–89		YRYSVYGET	WYRYSVYGETLE		1.6	HLA-DPA1*01:03/DPB1*04:01		78–89	YRYSVYGET	RYRYSVYGETLE	0.63
HLA-DPA1*01:03/DPB1*04:01	76–93		YRYSVYGET	YRWYRYSVYGETLEKQCN		1.6	HLA-DPA1*01:03/DPB1*04:01		76–93	YRYSVYGET	YRRYRYSVYGETLEKQCN	0.8
HLA-DPA1*01:03/DPB1*04:01	72–89		YRYSVYGET	KISEYRWYRYSVYGETLE		1.7	HLA-DPA1*01:03/DPB1*04:01		72–89	YRYSVYGET	KISEYRRYRYSVYGETLE	0.8
HLA-DPA1*01:03/DPB1*04:01	78–90		YRYSVYGET	WYRYSVYGETLEK		1.8	HLA-DPA1*01:03/DPB1*04:01		78–90	YRYSVYGET	RYRYSVYGETLEK	0.81
HLA-DPA1*01:03/DPB1*04:01	74–87		YRYSVYGET	SEYRWYRYSVYGET		1.8	HLA-DPA1*01:03/DPB1*04:01		74–87	YRYSVYGET	SEYRRYRYSVYGET	0.91
HLA-DPA1*01:03/DPB1*04:01	77–92		YRYSVYGET	RWYRYSVYGETLEKQC		1.8	HLA-DPA1*01:03/DPB1*04:01		77–92	YRYSVYGET	RRYRYSVYGETLEKQC	0.97
HLA-DPA1*01:03/DPB1*04:01	73–88		YRYSVYGET	ISEYRWYRYSVYGETL		2	HLA-DPA1*01:03/DPB1*04:01		73–88	YRYSVYGET	ISEYRRYRYSVYGETL	1.2
HLA-DPA1*01:03/DPB1*04:01	77–93		YRYSVYGET	RWYRYSVYGETLEKQCN		2.6	HLA-DPA1*01:03/DPB1*04:01		77–93	YRYSVYGET	RRYRYSVYGETLEKQCN	1.5
HLA-DPA1*01:03/DPB1*04:01	72–88		YRYSVYGET	KISEYRWYRYSVYGETL		2.6	HLA-DPA1*01:03/DPB1*04:01		72–88	YRYSVYGET	KISEYRRYRYSVYGETL	1.5
HLA-DPA1*01:03/DPB1*04:01	73–87		YRYSVYGET	ISEYRWYRYSVYGET		2.8	HLA-DPA1*01:03/DPB1*04:01		73–87	YRYSVYGET	ISEYRRYRYSVYGET	1.6
HLA-DPA1*01:03/DPB1*04:01	77–94		YRYSVYGET	RWYRYSVYGETLEKQCNK		3.3	HLA-DPA1*01:03/DPB1*04:01		77–94	YRYSVYGET	RRYRYSVYGETLEKQCNK	2
HLA-DPA1*01:03/DPB1*04:01	71–88		YRYSVYGET	SKISEYRWYRYSVYGETL		3.3	HLA-DPA1*01:03/DPB1*04:01		71–88	YRYSVYGET	SKISEYRRYRYSVYGETL	1.9
HLA-DRB1*15:02	74–85		YRWYRYSVY	SEYRWYRYSVYG		3.5	HLA-DRB1*15:02		74–85	YRRYRYSVY	SEYRRYRYSVYG	3.5
HLA-DPA1*01:03/DPB1*04:01	78–91		YRYSVYGET	WYRYSVYGETLEKQ		3.9	HLA-DPA1*01:03/DPB1*04:01		78–91	YRYSVYGET	RYRYSVYGETLEKQ	2
HLA-DPA1*01:03/DPB1*04:01	72–87		YRYSVYGET	KISEYRWYRYSVYGET		3.8	HLA-DPA1*01:03/DPB1*04:01		72–87	YRYSVYGET	KISEYRRYRYSVYGET	2.3
HLA-DRB1*15:02	78–90		YSVYGETLE	WYRYSVYGETLEK		3.6	HLA-DRB1*15:02		78–90	YSVYGETLE	RYRYSVYGETLEK	1.7
HLA-DRB1*15:02	78–91		YSVYGETLE	WYRYSVYGETLEKQ		3.6	HLA-DRB1*15:02		78–91	YSVYGETLE	RYRYSVYGETLEKQ	1.6
HLA-DRB1*15:02	77–91		YSVYGETLE	RWYRYSVYGETLEKQ		4.8	HLA-DRB1*15:02		77–91	YSVYGETLE	RRYRYSVYGETLEKQ	2.4
HPV35 E6 WT							HPV35 E6 W78R					
Allele	Length		Core peptide	Peptide		Score	Allele		Length	Core peptide	Peptide	Score
HLA-DRB1*15:02	76–87		WYRYSVYGE	YRWYRYSVYGET		2	HLA-DRB1*15:02		68–79	YSKISEYRR	KFYSKISEYRRY	3.1
HLA-DRB1*15:02	75–87		WYRYSVYGE	EYRWYRYSVYGET		2.2	HLA-DRB1*15:02		77–90	YSVYGETLE	RRYRYSVYGETLEK	3.1
HLA-DRB1*15:02	75–86		WYRYSVYGE	EYRWYRYSVYGE		2.4	HLA-DRB1*15:02		76–91	YSVYGETLE	YRRYRYSVYGETLEKQ	3.1
HLA-DRB1*15:02	74–87		WYRYSVYGE	SEYRWYRYSVYGET		3.5	HLA-DRB1*15:02		78–89	YSVYGETLE	RYRYSVYGETLE	3.6
HLA-DRB1*15:02	75–89		WYRYSVYGE	EYRWYRYSVYGETLE		3.8	HLA-DRB1*15:02		76–90	YSVYGETLE	YRRYRYSVYGETLEK	4.6
HLA-DRB1*15:02	74–86		YRWYRYSVY	SEYRWYRYSVYGE		4	HLA-DRB1*15:02		68–80	YSKISEYRR	KFYSKISEYRRYR	4.8
HLA-DRB1*15:02	74–89		WYRYSVYGE	SEYRWYRYSVYGETLE		4.2	HLA-DRB1*15:02		67–79	YSKISEYRR	LKFYSKISEYRRY	4.8
HLA-DRB1*15:02	75–90		WYRYSVYGE	EYRWYRYSVYGETLEK		4.8	HLA-DRB1*15:02		77–92	YSVYGETLE	RRYRYSVYGETLEKQC	4.9
HLA-DRB1*15:02	73–87		WYRYSVYGE	ISEYRWYRYSVYGET		4.9	HLA-DRB1*15:02		74–91	YSVYGETLE	SEYRRYRYSVYGETLEKQ	5
HLA-DRB1*15:02	76–88		WYRYSVYGE	YRWYRYSVYGETL		5						
HLA-DRB1*15:02	74–88		WYRYSVYGE	SEYRWYRYSVYGETL		5						
HLA-DQA1*01:01/DQB1*05:01	75–86		WYRYSVYGE	EYRWYRYSVYGE		3.9	HLA-DQA1*01:01/DQB1*05:01		78–89	YSVYGETLE	RYRYSVYGETLE	2.7
HLA-DQA1*01:01/DQB1*05:01	75–87		WYRYSVYGE	EYRWYRYSVYGET		3.9	HLA-DQA1*01:01/DQB1*05:01		78–90	YSVYGETLE	RYRYSVYGETLEK	4.1
HLA-DQA1*01:01/DQB1*05:01	78–89		YSVYGETLE	WYRYSVYGETLE		4.6	HLA-DQA1*01:01/DQB1*05:01		78–91	YSVYGETLE	RYRYSVYGETLEKQ	4.6
HLA-DPA1*01:03/DPB1*04:01	74–85		YRWYRYSVY	SEYRWYRYSVYG		4.6	HLA-DPA1*01:03/DPB1*04:01		71–87	YRYSVYGET	SKISEYRRYRYSVYGET	3
HLA-DPA1*01:03/DPB1*04:01	71–87		YRYSVYGET	SKISEYRWYRYSVYGET		5	HLA-DPA1*01:03/DPB1*04:01		70–87	YRYSVYGET	YSKISEYRRYRYSVYGET	3.8
							HLA-DPA1*01:03/DPB1*04:01		78–92	YRYSVYGET	RYRYSVYGETLEKQC	4
							HLA-DRB1*12:02		50–61	IVYREGQPY	LCIVYREGQPYG	0.95
							HLA-DRB1*12:02		71–82	ISEYRRYRY	SKISEYRRYRYS	1.9
							HLA-DRB1*12:02		70–81	ISEYRRYRY	YSKISEYRRYRY	3.7
							HLA-DRB1*12:02		70–82	ISEYRRYRY	YSKISEYRRYRYS	3.9
							HLA-DRB1*12:02		71–83	ISEYRRYRY	SKISEYRRYRYSV	4.4
							HLA-DRB1*15:02		78–92	YSVYGETLE	RYRYSVYGETLEKQC	4.4
HPV35 E7 WT							HPV35 E7 H23Y					
Allele	Length	Core peptide			Peptide	Score	Allele	Length		Core peptide	Peptide	Score
HLA-DQA1*01:01/DQB1*05:01	6–23	LQDYVLDLE			TTLQDYVLDLEPEATDLY	2.9	HLA-DQA1*01:01/DQB1*05:01	6–23		LQDYVLDLE	TTLQDYVLDLEPEATDLH	2.9

Note : * is one of the constituent symbols of alleles

**Fig. 4:**
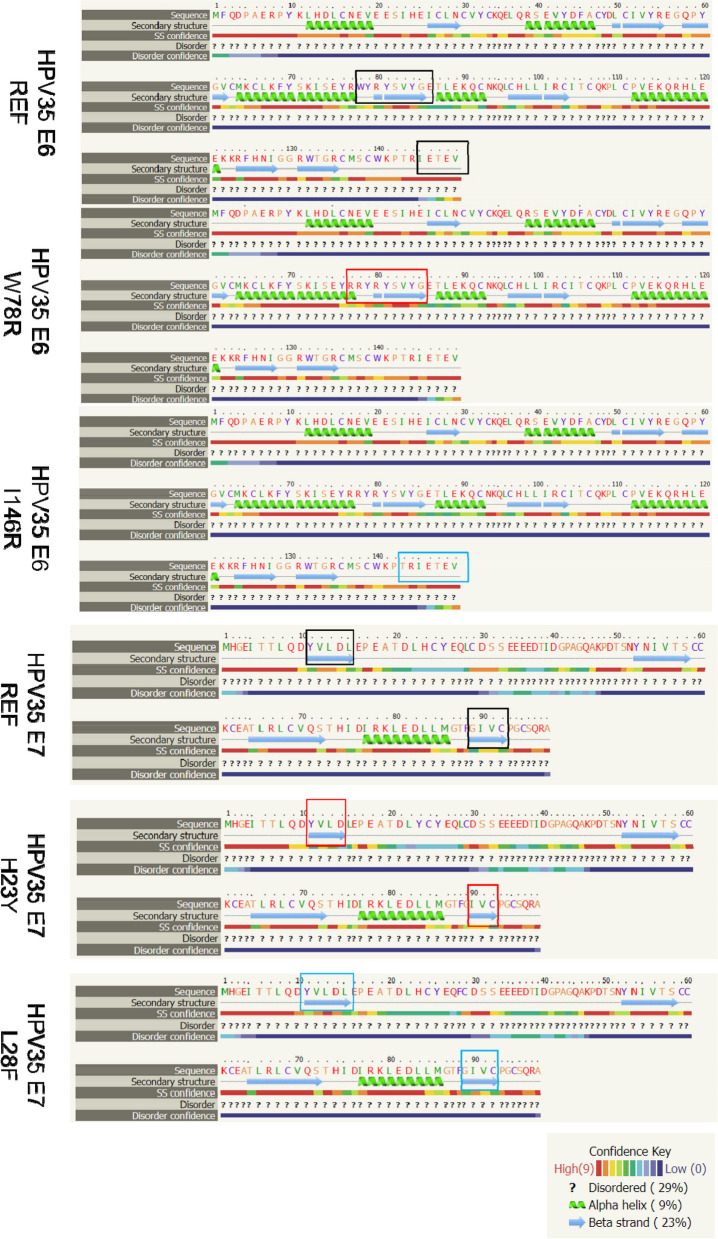
The effect of non-synonymous mutation on HPV-35 E6, E7 protein structure

In HPV-35 E7 reference sequence, 27 HLA-I and 147 HLA-II epitopes were predicted. In H23Y variant, 28 HLA-I and 147 HLA-II epitopes were predicted, H23Y made a new HLA-I epitopes. In L28F variant, 27 HLA-I and 147 HLA-II epitopes were predicted, L28F have no influence on HLA-I and HLA-II epitopes (Tables 3 and 4) (Fig. 4).

For the design of therapeutic vaccine, epitopes devoid of mutation sites were concatenated into continuous polypeptide sequences. This approach aims to preserve the native structure and immune recognition properties of the epitopes, thereby enhancing the potential efficacy of the vaccine. In HPV-35 E6, HLA-I predicted epitopes occurred in 1–19, 17–32, 35–45, 41–55, 52–64, 67–77, 79–96, 124–140 segment, and HLA-II were in 45–62, 79–91, 120–135; 81-88YSVYGETL and 52-60IVYREGQPY were the most frequency E6 HLA-I epitopes, as well as 15 potential HLA-I epitopes located in HLA-I and HLA-II common segment, details were shown in Tables 5 and 6.

**Table 5:** The HLA-I, HLA-II distribution analysis of HPV-35 E6 and E7

Target protein	Distribution of HLA-I epitopes (epitope’s number)	Distribution of HLA-II epitopes (epitope’s number)
35 E6	1–19 (18)	
	17–32 (7)	
	35–45 (6)	
	41–55 (9)	45–62 (14)
	52–64 (5)	
	67–77 (11)	
	79–96 (18)	79–91 (5)
	124–140 (5)	120–135 (8)
35 E7	3–19 (10)	1–22 (83)
	38–55 (9)	30–49 (4)
	70–80 (4)	70–91 (60)
	79–88 (3)	
	90–98 (1)

**Table 6:** The T-cell optimal epitopes of HPV-35 E6, E7

Target	Location	Epitopes	Optimal PR/ No	HLA-II Core epitopes	Target	Location	Epitopes	Optimal PR/ No	HLA-II Core epitopes
HPV 35 E6	45–54	FACYDLCIVY	0.12 /3		HPV 35 E7	3–11	GEITTLQDY	0.87/1	
	46–54	ACYDLCIVY	0.47/3			3–13	GEITTLQDYVL	0.1/1	ITTLQDYVL
	46–55	ACYDLCIVYR	1/1			5–13	ITTLQDYVL	0.75/2	ITTLQDYVL
	52–60	IVYREGQPY	0.02/4	IVYREGQPY		7–15	TLQDYVLDL	0.09//4	
	79–88	YRYSVYGETL	0.39/1	YRYSVYGET		11–19	YVLDLEPEA	0.26/2	YVLDLEPEA
	79–90	YRYSVYGETLEK	0.5/1	YRYSVYGET		38–47	TIDGPAGQAK	0.4/1	
	80–88	RYSVYGETL	0.05/2			70–79	VQSTHIDIRK	0.79/1	
	81–88	YSVYGETL	0.14/4			71–79	QSTHIDIRK	0.46/1	
	80–90	RYSVYGETLEK	0.02/1	YSVYGETLE		72–80	STHIDIRKL	0.58/2	
	81–90	YSVYGETLEK	0.09/1	YSVYGETLE		79–87	KLEDLLMGT	0.95/1	
	82–90	SVYGETLEK	0.01//1			79–88	KLEDLLMGTF	0.66/1	
	82–91	SVYGETLEKQ	0.5/1			80–88	LEDLLMGTF	0.47/1	
	124–132	RFHNIGGRW	0.33//2						
	126–135	HNIGGRWTGR	0.28/1						
	127–135	NIGGRWTGR	0.22/1						

In HPV-35 E7, HLA-I predicted epitopes occurred in 3–19, 38–55, 70–80, 79–88, 90–98 segment, and HLA-II were in 1–22, 30–49, 70–91; 45-53QAKPDTSNY was the lowest PR (0.1) and most frequently E7 HLA-I epitopes, as well as 12 HLA-I　epitopes located in HLA-I and HLA-II common segment, details were shown in Tables 5 and 6.

The HLA-II epitope binding core is a key important region for CD4^+^ T-cell recognition, binding, and activation, residing in the peptide-binding groove of HLA-II molecules. Both CD4^+^ and CD8^+^ T-cell participate in HPV-related immune response, CD8^+^ T-cell are considered as the primary effectors in eliminating HPV-infected and cervical cancer cells. Consequently, optimal epitopes that are the potential MHC-I epitopes that contain at least one core region of HLA-II-binding peptide is valuable. These epitopes can potentially activate both CD4^+^ and CD8^+^ T-cells, thereby enhancing the breadth and effectiveness of the immune response against HPV.

Finally, the optimal epitopes were selected as fellow step: 1. Selected the epitopes do not contain mutation sites. 2. Selected epitopes located in the common region of HLA-I and HLA-II predicted epitopes. 3. Selected epitopes contain at least one HLA-II binding core region peptide. 4. Epitopes that bind more HLA-I alleles and lower the percentile rank is better. 5. The integrated HLA-I epitope peptides are also excellent vaccine targets peptides in HLA-I and HLA-II predicted epitopes common region and called optimal peptides. In HPV-35 E6, 5 optimal epitopes were selected, they were 52-60IVYREGQPY, 79-88YRYSVYGETL, 79-90YRYSVYGETLEK, 80-90RYSVYGETLEK, 81-90YSVYGETLEK; the optimal peptides were 79-91YRYSVYGETLEKQ, 45-60FACYDLCIVYREGQPY, 124-135RFHNIGGRWTGR. In HPV-35 E7, optimal epitopes were 3-13GEITTLQDYVL, 5-13ITTLQDYVL, 11-19YVLDLEPEA; the optimal peptides: 3-19GEITTLQDYVLDLEPEA, 38-47TIDGPAGQA, 70-88VQSTHIDRKLEDLLMGT.

## Discussion

The paucity of data on HPV-35 hinders our understanding of this virus, highlighting the significance of conducting research in Sichuan to establish a theoretical framework for the prevention and treatment of HPV35. The HPV characteristics vary across different geographical and ethnic groups, highlighting the significance of conducting research in Sichuan to establish a theoretical framework for the prevention and treatment of HPV35. HPV-35 is the only HR α-9 genus HPV that is understudied, largely due to its historically low prevalence. International research has identified HPV-35 as a significant risk factor for cervical cancer, ranking it second only to HPV16, in terms of pathogenicity, with a threat to human health that is comparable to that of HPV16. The limited data on HPV35, coupled with its low infection rate, can lead to a tendency to overlook its risks, yet latent HPV-35 may pose an even greater threat than HPV16. In our study, only 31 HPV35-positive samples were detected among 406 positive samples (7.67%), which aligns with the 8.1% positive detection data reported by Basto Diogo Lisboa et al. The low infection rate of HPV-35 reflects the scarcity of genomic data, which may be attributed to the highly conservative nature of HPV-35 mutations, rendering them less susceptible to genomic mutations and inheritance within host cells. Additionally, HPV-35 may be less likely to cross-infect with other HPV types or variants, or it may have a longer incubation period in host cells than other HPV types, making it more difficult to detect [28]. The decades-long incubation period of HPV-35 compounds our challenges, and the lack of comprehensive data on HPV-35 impedes our awareness of its implications. Given the distinct characteristics of HPV across different geographical and ethnic groups, it is imperative to conduct systematic research in Sichuan to provide a solid theoretical foundation for HPV-35 prevention and treatment.

HPV-35 isolates are genetically related to HPV31 and HPV16, and they shared a recent common ancestor with HPV33 and HPV58 isolates [28]. In our study, 5 nucleotide-acid mutations in E6 (2 non-synonymous mutations: T232C (W78R) and T434G (I146R), 3 synonymous mutations: T18C, T27C, and G262C) were detected and 3 in E7 (2 non-synonymous mutations: C67T (H23Y) and G84T (L28F), 1 synonymous mutation: C114T), and due to the limitation of sample, some mutation sites may not have been identified. The variants of HPV-35 E6 and E7 in Sichuan, China, are distinct and do not belong to the well-known A1 and A2 series. They exhibit strong regional characteristics. The homology between these variants and the reference sequences is notably higher for the E6 variant compared to the E7 variant. Furthermore, the E7 variant appears to have a more distant evolutionary lineage. HPV 35E6/E7 01, HPV 35 E6/E7 10 was found to have the highest degree of homology with the reference sequence among the HPV-35 E6/E7 sample sequence. This isolate in question likely originated from a reference sequence, with HPV-35 E6/E7 01, 35 E6/E7 10 and the A1 line belonging to the same clade, while the remaining sequences also grouped within the same clade. As observed by Chen Zigui and Basto Diogo Lisboa et al., all variants of HPV-35 are highly conserved, displaying minimal intratype genetic diversity, and failing to meet the criteria for classification into multiple lineages. The highest infection rate for HPV-35 was found in the 56-years age bracket (42.9%) [29]. These finding suggest the prevalence pattern may be influenced by the existence of another variant lineage of HPV-35 that could be present in isolated and/or unsampled populations. Intratype mutation within host cell may be infrequent, and the virus may have a longer incubation period, making it more challenging to detect. The mutant strains in the Sichuan area of China exhibit distinct regional characteristics, which should be taken into account, when designing vaccines and probes specific to the Sichuan population. The mutation rate in the E6 gene was higher than that of E7 (78.94%/52.63%). Consequently, the more stable E7 should be prioritized, when considering the design of probe for HPV35.

HR-HPV E6, E7 are critical oncoprotein that differ from those of LR-HPV. They are capable of interacting with RB, p53 tumor suppressor proteins and several cycle regulatory proteins. *E6, E7* non-synonymous mutations can result in alterations to the amino acid composition, protein structure and function. For instance, the mutations W78R, I145R in E6 do not reside within the alpha-helix or beta-sheet region. However, I145R is located in the highly conserved carboxyl terminal, which is involved in binding to PDZ domain-containing proteins that play roles in intracellular cell adhesion, cell linkage and cell polarization. W78R is situated near the Znic-figure and within the groove formed by two Znic-blinding figures, which are crucial for p53 binding. In E7, the mutations H23Y, L28F are not located within the alpha-helix or beta-sheet regions either. The region spanning amino acids 16–37 of E7 exhibits a high affinity for RB protein and is considered the active region of E7 protein. The residues 21–29 positions constitute the pRB binding regions, with the LXCXE motif being strictly conserved as the phosphorylation site of casein kinase (CK) II. Both H23Y and L28F are positioned within this functional region of E7. Additionally, H23Y induces a change from an alpha-helix to a coil conformation at positions 15 and 89, which are distant from the site of substitution. The structural change at the 89th amino acid caused by H23Y is in close proximity to amino acids 58, 61, 91, and 94 of HPV E7, which are zinc-binding sites involved in the binding of various functional proteins. The Znic-binding figures, carboxyl terminal and PDZ domain binding motif are essential structural and functional domains of E6, E7. Substitutions in these regions can lead to differences in the binding abilities of these oncoproteins to host proteins such as p53, pRB, and others, which is considered key factors in their carcinogenic potential.

HPV E6, E7, which are early protein, are considered prime candidates for prophylactic vaccine targets. When these proteins are digested and presented to the body's immune system, they can active immune response that eliminate HPV infection and reduces the risk of HPV-related diseases. E6 and E7 T-cell epitopes have been found to locate in the C-terminal segment and Zinc-finger region, with HLA-A epitopes potentially also be in the N-terminal. Research has indicated that mutations can impact the effectiveness of HPV vaccine, particularly those designed to target specific epitopes, as the efficacy may be compromised if mutations are not considered. Non-synonymous mutation can alter epitope characteristics. For instance, the W78R mutation in E6 has been shown to increase the affinity and number of HLA-I epitopes, potentially leading to the emergence of new and improved epitopes. Similarly, the I145R mutation enhances HLA-I epitope affinity and number. H23Y in E7 increases HLA-I epitope affinity as well. The E7 protein exhibits a greater number and affinity of epitopes compared to E6, suggesting that E7 may not be as readily recognized by host cells, and its carcinogenic potential may exceed that of E6. These hypotheses require confirmation in subsequent studies. Positive selection sites play a crucial role in enhancing a species’ adaptability to its environment by increasing the frequency of mutated genes [30]. Analysis using the software package paml has revealed that the HPV-35 E6 positive selection site W78R (mutation risk:22/24) is a frequent non-synonymous mutation, indicating that W78R contributes to the adaptation of HPV-35 E6 and has become widely prevalent. The conformational and functional changes caused by this substitution may influence the virus’s pathogenicity [31]. Specific intramotypic mutations in HPV E6 can result in differences in virus infection ability and pathogenicity. For example, positive selection site D32E of HPV16, has been confirmed to be associated with the development of cervical cancer [17]; Similarly, W78R in HPV-35 E6, located in an active region, affects protein conformation, function and may reduce the immunogenicity of the peptide containing this sites. Substitutions at positive selection sites can impact protein structure, decrease epitope affinity, make HPV-infected cells more difficult to detect by the immune system, enhance HPV’s adaptability to the environment, improve the efficiency of virus infection, and potentially promote the development of cervical cancer.

This study represents the first investigation into the interplay between the protein structure, positive selection sites, epitopes, and pathogenicity of HPV-35 E6 and E7 in Sichuan Province. It aims to elucidate the relationship between HPV E6, E7 and the varying abilities of HPV to infect, with the ultimate goal of enhancing the development of therapeutic vaccine targeting HPV-35 E6 and E7 in Sichuan Province.

## Conclusion

HPV-35 variants exhibit high conservation and can remain latent in host cells for extended periods. Consequently, the design of detection probes for HPV-35 should be tailored to enhance the detection rate. Early detection and treatment of HPV-35 infections carry significant clinical and social importance. The enrichment of HPV-35 polymorphism data will provide a theoretical foundation for the development of effective prevention and treatment strategies.

Among the 406 HPV-positive cell specimens, 31 HPV-35 positive samples were detected (7.63%), 2 non-synonymous mutations were detected in E6, E7 respectively,. These mutations are in active region, and have the potential to affect protein conformation, function and immunogenicity of those peptide containing these sites. The potential epitopes of HPV-35 E6 were: 52-60IVYREGQPY, 79-88YRYSVY GETL, 79-90YRYSVYGETLEK, 80-90RYSVYGETLEK, 81-90YSVYGETLEK, optimal peptides were 79–91 YRYSVYGETLEKQ, 45-60FACYDLCIVYREGQPY, 124-135RFHNIGGRWTGR; potential epitopes of HPV-35 E7 were: 3-13GEITTL QDYVL, 5-13ITTLQDYVL, 11-19YVLDLEPEA, optimal peptides were 3-19GEITT LQDYVLDLEPEA, 38-47TIDGPAGQA, 70-88VQSTHIDRKLEDLLMGT.

## Supplementary Information

**Additional file 1: Table S1.** The selected HLA-I and HLA-IIalleles with average frequency over 5% in Chinese. **Table S2.** The HLA-I predicted epitopes of HPV-35 E6. **Table S3.** The HLA-I predicted epitopes of HPV-35 E7. **Table S4.** The HLA-II predicted epitopes of HPV-35 E6. **Table S5.** The HLA-II predicted epitopes of HPV-35 E7.

## Data Availability

No datasets were generated or analysed during the current study.
