# Supplementary material for: The polymorphism analysis and therapy vaccine target epitopes screening of HPV-35 E6 E7 among the threaten α-9 HPV in Sichuan area
Source: Virol J. 2024 Sep 9;21:213. doi: 10.1186/s12985-024-02357-3 (PMC11384679; doi:10.1186/s12985-024-02357-3)
Supplement: Supplementary file 1 — Additional file 1: Table S1. The selected HLA-I and HLA-IIalleles with average frequency over 5% in Chinese. Table S2. The HLA-I predicted epitopes of HPV-35 E6. Table S3. The HLA-I predicted epitopes of HPV-35 E7. Table S4. The HLA-II predicted epitopes of HPV-35 E6. Table S5. The HLA-II predicted epitopes of HPV-35 E7. [file 12985_2024_2357_MOESM1_ESM.docx]

The polymorphism analysis and therapy vaccine target epitopes screening of HPV-35 E6 E7 among the threaten α-9 HPV in Sichuan area

Jiaoyu He^1,2^, Tianjun Li^1,2^, Chunlan Cheng^2^, Ning Li^2^, Peng Gao^2^, Dan Lei^1,2,3^, Rong Liang^3^, Xianping Ding^1,2^.

1 Key Laboratory of Bio-Resources and Eco-Environment of Ministry of Education, College of Life Sciences, Sichuan University, Chengdu 610065, Sichuan, P.R.China.

2 Department of Clinical laboratory, Sichuan Provincial Maternity and Child Health Care Hospital, chengdu, P.R.China;

3 Department of Ultrasound, Sichuan Provincial Maternity and Child Health Care Hospital, chengdu, P.R.China.

* Corresponding author:

Key Laboratory of Bio-Resource and Eco-Environment of Ministry of Education, College of Life Sciences, Sichuan University, Chengdu 610065, Sichuan, PR China.

E-mail: brainding@scu.edu.cn

Telephone: 86-028-85413096

Fax: 86-028-85415895

Email address:

Jiaoyu He: [1061355567@qq.com](mailto:1061355567@qq.com); Tianjun Li: [403873142@qq.com](mailto:403873142@qq.com)；

Chunlan Cheng: [350680397@qq.com](mailto:350680397@qq.com); Ning Li: [369432050@163.com](mailto:369432050@163.com);

Peng Gao: [gaopeng180@qq.com](mailto:gaopeng180@qq.com)； Dan Lei: [1875473985@qq.com](mailto:1875473985@qq.com);

Rong Liang: [68155388@qq.com](mailto:68155388@qq.com); Xianping Ding: brainding@scu.edu.cn.

Table S1. The selected HLA-Ⅰ and HLA-Ⅱ alleles with average frequency over 5% in Chinese population

| HLA-I alleles | | | | HLA-II alleles | |
| --- | --- | --- | --- | --- | --- |
| Alleles | Average frequency | Alleles | Average frequency | Alleles | Average frequency |
| HLA-A*11:01 | 27.7% | HLA-A*24:02 | 17.2% | DPB1*04:01 | 17.1% |
| HLA-C*01:02 | 16.9% | HLA-B*40:01 | 14.9% | DRB1*14:01 | 13.4% |
| HLA-C*03:04 | 12.8% | HLA-C*08:01 | 12.6% | DRB1*12:02 | 11.4% |
| HLA-B*46:01 | 11.5% | HLA-A*33:03 | 11.5% | DQB1*05:01 | 10.9% |
| HLA-B*58:01 | 8.9% | HLA-C*03:02 | 8.7% | DQB1*02:01 | 9.3% |
| HLA-B*13:01 | 8.2% | HLA-B*15:02 | 7.1% | DRB1*15:02 | 5.6% |
| HLA-A*02:01 | 5.3% |  |  |  |  |

Note: Lacking information about the frequency in Chinese individuals, the data from Asian individuals were used.

Table S2. The HLA-Ⅰ predicted epitopes of HPV-35 E6

| HPV35E6 REF | | | | HPV35E6 W78R | | | | HPV35E6 I145R | | | |
| --- | --- | --- | --- | --- | --- | --- | --- | --- | --- | --- | --- |
| Allele | Location | Peptide | PR | Allele | Location | Peptide | PR | Allele | Location | Peptide | PR |
| HLA-A*11:01 | 82-90 | SVYGETLEK | 0.01 | HLA-A*11:01 | 82-90 | SVYGETLEK | 0.01 | HLA-A*11:01 | 82-90 | SVYGETLEK | 0.01 |
| HLA-B*58:01 | 70-78 | YSKISEYRW | 0.01 | HLA-A*11:01 | 80-90 | RYSVYGETLEK | 0.02 | HLA-B*58:01 | 70-78 | YSKISEYRW | 0.01 |
| HLA-A*11:01 | 80-90 | RYSVYGETLEK | 0.02 | HLA-B*15:02 | 52-60 | IVYREGQPY | 0.02 | HLA-A*11:01 | 80-90 | RYSVYGETLEK | 0.02 |
| HLA-B*15:02 | 52-60 | IVYREGQPY | 0.02 | HLA-B*46:01 | 52-60 | IVYREGQPY | 0.03 | HLA-B*15:02 | 52-60 | IVYREGQPY | 0.02 |
| HLA-B*46:01 | 52-60 | IVYREGQPY | 0.03 | HLA-B*15:02 | 2-10 | FQDPAERPY | 0.04 | HLA-B*46:01 | 52-60 | IVYREGQPY | 0.03 |
| HLA-B*15:02 | 2-10 | FQDPAERPY | 0.04 | HLA-A*24:02 | 80-88 | RYSVYGETL | 0.05 | HLA-B*15:02 | 2-10 | FQDPAERPY | 0.04 |
| HLA-A*24:02 | 80-88 | RYSVYGETL | 0.05 | HLA-C*03:02 | 2-10 | FQDPAERPY | 0.05 | HLA-A*24:02 | 80-88 | RYSVYGETL | 0.05 |
| HLA-C*03:02 | 2-10 | FQDPAERPY | 0.05 | HLA-A*02:01 | 11-19 | KLHDLCNEV | 0.06 | HLA-C*03:02 | 2-10 | FQDPAERPY | 0.05 |
| HLA-A*33:03 | 72-80 | KISEYRWYR | 0.05 | HLA-C*03:02 | 52-60 | IVYREGQPY | 0.06 | HLA-A*33:03 | 72-80 | KISEYRWYR | 0.05 |
| HLA-A*02:01 | 11-19 | KLHDLCNEV | 0.06 | HLA-A*33:03 | 69-77 | FYSKISEYR | 0.06 | HLA-A*02:01 | 11-19 | KLHDLCNEV | 0.06 |
| HLA-C*03:02 | 52-60 | IVYREGQPY | 0.06 | HLA-A*11:01 | 81-90 | YSVYGETLEK | 0.09 | HLA-C*03:02 | 52-60 | IVYREGQPY | 0.06 |
| HLA-A*33:03 | 69-77 | FYSKISEYR | 0.06 | HLA-B*46:01 | 45-54 | FACYDLCIVY | 0.12 | HLA-A*33:03 | 69-77 | FYSKISEYR | 0.06 |
| HLA-A*11:01 | 81-90 | YSVYGETLEK | 0.09 | HLA-B*46:01 | 2-10 | FQDPAERPY | 0.12 | HLA-A*11:01 | 81-90 | YSVYGETLEK | 0.09 |
| HLA-A*24:02 | 69-78 | FYSKISEYRW | 0.12 | HLA-B*13:01 | 2-12 | FQDPAERPYKL | 0.12 | HLA-A*24:02 | 69-78 | FYSKISEYRW | 0.12 |
| HLA-B*46:01 | 45-54 | FACYDLCIVY | 0.12 | HLA-C*03:04 | 81-88 | YSVYGETL | 0.14 | HLA-B*46:01 | 45-54 | FACYDLCIVY | 0.12 |
| HLA-B*46:01 | 2-10 | FQDPAERPY | 0.12 | HLA-A*11:01 | 82-94 | SVYGETLEKQCNK | 0.16 | HLA-B*46:01 | 2-10 | FQDPAERPY | 0.12 |
| HLA-B*13:01 | 2-12 | FQDPAERPYKL | 0.12 | HLA-A*33:03 | 70-78 | YSKISEYRR | 0.17 | HLA-B*13:01 | 2-12 | FQDPAERPYKL | 0.12 |
| HLA-C*03:04 | 81-88 | YSVYGETL | 0.14 | HLA-C*08:01 | 2-12 | FQDPAERPYKL | 0.17 | HLA-C*03:04 | 81-88 | YSVYGETL | 0.14 |
| HLA-A*11:01 | 82-94 | SVYGETLEKQCNK | 0.16 | HLA-A*11:01 | 78-90 | RYRYSVYGETLEK | 0.19 | HLA-A*11:01 | 82-94 | SVYGETLEKQCNK | 0.16 |
| HLA-C*08:01 | 2-12 | FQDPAERPYKL | 0.17 | HLA-B*15:02 | 1-10 | MFQDPAERPY | 0.2 | HLA-C*08:01 | 2-12 | FQDPAERPYKL | 0.17 |
| HLA-B*15:02 | 1-10 | MFQDPAERPY | 0.2 | HLA-B*15:02 | 68-76 | KFYSKISEY | 0.21 | HLA-A*33:03 | 137-145 | MSCWKPTRR | 0.2 |
| HLA-A*33:03 | 71-80 | SKISEYRWYR | 0.21 | HLA-A*33:03 | 127-135 | NIGGRWTGR | 0.22 | HLA-B*15:02 | 1-10 | MFQDPAERPY | 0.2 |
| HLA-B*15:02 | 68-76 | KFYSKISEY | 0.21 | HLA-B*40:01 | 19-28 | VEESIHEICL | 0.24 | HLA-A*33:03 | 71-80 | SKISEYRWYR | 0.21 |
| HLA-A*33:03 | 127-135 | NIGGRWTGR | 0.22 | HLA-C*08:01 | 2-10 | FQDPAERPY | 0.24 | HLA-B*15:02 | 68-76 | KFYSKISEY | 0.21 |
| HLA-B*58:01 | 69-78 | FYSKISEYRW | 0.23 | HLA-A*33:03 | 72-80 | KISEYRRYR | 0.25 | HLA-A*33:03 | 127-135 | NIGGRWTGR | 0.22 |
| HLA-B*40:01 | 19-28 | VEESIHEICL | 0.24 | HLA-B*46:01 | 68-76 | KFYSKISEY | 0.26 | HLA-B*58:01 | 69-78 | FYSKISEYRW | 0.23 |
| HLA-C*08:01 | 2-10 | FQDPAERPY | 0.24 | HLA-C*08:01 | 81-88 | YSVYGETL | 0.27 | HLA-B*40:01 | 19-28 | VEESIHEICL | 0.24 |
| HLA-B*46:01 | 68-76 | KFYSKISEY | 0.26 | HLA-A*33:03 | 126-135 | HNIGGRWTGR | 0.28 | HLA-C*08:01 | 2-10 | FQDPAERPY | 0.24 |
| HLA-B*58:01 | 68-78 | KFYSKISEYRW | 0.27 | HLA-A*33:03 | 69-78 | FYSKISEYRR | 0.29 | HLA-B*46:01 | 68-76 | KFYSKISEY | 0.26 |
| HLA-C*08:01 | 81-88 | YSVYGETL | 0.27 | HLA-C*03:02 | 45-54 | FACYDLCIVY | 0.29 | HLA-B*58:01 | 68-78 | KFYSKISEYRW | 0.27 |
| HLA-A*33:03 | 126-135 | HNIGGRWTGR | 0.28 | HLA-C*03:02 | 68-76 | KFYSKISEY | 0.3 | HLA-C*08:01 | 81-88 | YSVYGETL | 0.27 |
| HLA-A*11:01 | 72-80 | KISEYRWYR | 0.28 | HLA-A*33:03 | 68-77 | KFYSKISEYR | 0.31 | HLA-A*33:03 | 126-135 | HNIGGRWTGR | 0.28 |
| HLA-A*33:03 | 70-80 | YSKISEYRWYR | 0.29 | HLA-B*40:01 | 20-28 | EESIHEICL | 0.31 | HLA-A*11:01 | 72-80 | KISEYRWYR | 0.28 |
| HLA-C*03:02 | 45-54 | FACYDLCIVY | 0.29 | HLA-A*24:02 | 124-132 | RFHNIGGRW | 0.33 | HLA-A*33:03 | 70-80 | YSKISEYRWYR | 0.29 |
| HLA-C*03:02 | 68-76 | KFYSKISEY | 0.3 | HLA-B*40:01 | 88-96 | LEKQCNKQL | 0.35 | HLA-C*03:02 | 45-54 | FACYDLCIVY | 0.29 |
| HLA-A*33:03 | 68-77 | KFYSKISEYR | 0.31 | HLA-B*15:02 | 36-43 | ELQRSEVY | 0.38 | HLA-C*03:02 | 68-76 | KFYSKISEY | 0.3 |
| HLA-B*40:01 | 20-28 | EESIHEICL | 0.31 | HLA-A*24:02 | 79-88 | YRYSVYGETL | 0.39 | HLA-A*33:03 | 68-77 | KFYSKISEYR | 0.31 |
| HLA-A*24:02 | 124-132 | RFHNIGGRW | 0.33 | HLA-B*40:01 | 55-64 | REGQPYGVCM | 0.43 | HLA-B*40:01 | 20-28 | EESIHEICL | 0.31 |
| HLA-B*40:01 | 88-96 | LEKQCNKQL | 0.35 | HLA-B*15:02 | 41-48 | EVYDFACY | 0.47 | HLA-A*24:02 | 124-132 | RFHNIGGRW | 0.33 |
| HLA-A*24:02 | 68-78 | KFYSKISEYRW | 0.35 | HLA-B*46:01 | 46-54 | ACYDLCIVY | 0.47 | HLA-B*40:01 | 88-96 | LEKQCNKQL | 0.35 |
| HLA-B*15:02 | 36-43 | ELQRSEVY | 0.38 | HLA-A*11:01 | 77-90 | RRYRYSVYGETLEK | 0.48 | HLA-A*24:02 | 68-78 | KFYSKISEYRW | 0.35 |
| HLA-A*24:02 | 79-88 | YRYSVYGETL | 0.39 | HLA-B*13:01 | 37-45 | LQRSEVYDF | 0.48 | HLA-B*15:02 | 36-43 | ELQRSEVY | 0.38 |
| HLA-B*40:01 | 55-64 | REGQPYGVCM | 0.43 | HLA-B*40:01 | 17-26 | NEVEESIHEI | 0.48 | HLA-A*24:02 | 79-88 | YRYSVYGETL | 0.39 |
| HLA-B*15:02 | 41-48 | EVYDFACY | 0.47 | HLA-A*11:01 | 79-90 | YRYSVYGETLEK | 0.5 | HLA-B*40:01 | 55-64 | REGQPYGVCM | 0.43 |
| HLA-B*46:01 | 46-54 | ACYDLCIVY | 0.47 | HLA-A*11:01 | 82-91 | SVYGETLEKQ | 0.5 | HLA-B*15:02 | 41-48 | EVYDFACY | 0.47 |
| HLA-A*11:01 | 77-90 | RWYRYSVYGETLEK | 0.48 | HLA-B*15:02 | 46-54 | ACYDLCIVY | 0.5 | HLA-B*46:01 | 46-54 | ACYDLCIVY | 0.47 |
| HLA-B*13:01 | 37-45 | LQRSEVYDF | 0.48 | HLA-B*15:02 | 37-45 | LQRSEVYDF | 0.51 | HLA-A*11:01 | 77-90 | RWYRYSVYGETLEK | 0.48 |
| HLA-B*40:01 | 17-26 | NEVEESIHEI | 0.48 | HLA-C*03:02 | 69-76 | FYSKISEY | 0.51 | HLA-B*13:01 | 37-45 | LQRSEVYDF | 0.48 |
| HLA-A*11:01 | 79-90 | YRYSVYGETLEK | 0.5 | HLA-A*11:01 | 85-94 | GETLEKQCNK | 0.52 | HLA-B*40:01 | 17-26 | NEVEESIHEI | 0.48 |
| HLA-A*11:01 | 82-91 | SVYGETLEKQ | 0.5 | HLA-B*15:02 | 45-54 | FACYDLCIVY | 0.53 | HLA-A*11:01 | 79-90 | YRYSVYGETLEK | 0.5 |
| HLA-B*15:02 | 46-54 | ACYDLCIVY | 0.5 | HLA-B*15:02 | 69-76 | FYSKISEY | 0.55 | HLA-A*11:01 | 82-91 | SVYGETLEKQ | 0.5 |
| HLA-B*15:02 | 37-45 | LQRSEVYDF | 0.51 | HLA-A*02:01 | 2-12 | FQDPAERPYKL | 0.55 | HLA-B*15:02 | 46-54 | ACYDLCIVY | 0.5 |
| HLA-C*03:02 | 69-76 | FYSKISEY | 0.51 | HLA-A*24:02 | 68-76 | KFYSKISEY | 0.55 | HLA-B*15:02 | 37-45 | LQRSEVYDF | 0.51 |
| HLA-A*11:01 | 85-94 | GETLEKQCNK | 0.52 | HLA-B*46:01 | 69-76 | FYSKISEY | 0.56 | HLA-C*03:02 | 69-76 | FYSKISEY | 0.51 |
| HLA-B*15:02 | 45-54 | FACYDLCIVY | 0.53 | HLA-C*08:01 | 18-26 | EVEESIHEI | 0.56 | HLA-A*11:01 | 85-94 | GETLEKQCNK | 0.52 |
| HLA-B*15:02 | 69-76 | FYSKISEY | 0.55 | HLA-C*03:04 | 2-10 | FQDPAERPY | 0.57 | HLA-B*15:02 | 45-54 | FACYDLCIVY | 0.53 |
| HLA-A*02:01 | 2-12 | FQDPAERPYKL | 0.55 | HLA-B*15:02 | 35-43 | QELQRSEVY | 0.59 | HLA-B*15:02 | 69-76 | FYSKISEY | 0.55 |
| HLA-A*24:02 | 68-76 | KFYSKISEY | 0.55 | HLA-B*40:01 | 19-26 | VEESIHEI | 0.59 | HLA-A*02:01 | 2-12 | FQDPAERPYKL | 0.55 |
| HLA-B*46:01 | 69-76 | FYSKISEY | 0.56 | HLA-B*40:01 | 35-43 | QELQRSEVY | 0.6 | HLA-A*24:02 | 68-76 | KFYSKISEY | 0.55 |
| HLA-C*08:01 | 18-26 | EVEESIHEI | 0.56 | HLA-C*03:02 | 81-88 | YSVYGETL | 0.61 | HLA-B*46:01 | 69-76 | FYSKISEY | 0.56 |
| HLA-C*03:04 | 2-10 | FQDPAERPY | 0.57 | HLA-A*24:02 | 78-88 | RYRYSVYGETL | 0.61 | HLA-C*08:01 | 18-26 | EVEESIHEI | 0.56 |
| HLA-B*15:02 | 35-43 | QELQRSEVY | 0.59 | HLA-B*13:01 | 2-10 | FQDPAERPY | 0.62 | HLA-C*03:04 | 2-10 | FQDPAERPY | 0.57 |
| HLA-B*40:01 | 19-26 | VEESIHEI | 0.59 | HLA-C*03:02 | 46-54 | ACYDLCIVY | 0.62 | HLA-B*15:02 | 35-43 | QELQRSEVY | 0.59 |
| HLA-B*40:01 | 35-43 | QELQRSEVY | 0.6 | HLA-A*33:03 | 71-80 | SKISEYRRYR | 0.63 | HLA-B*40:01 | 19-26 | VEESIHEI | 0.59 |
| HLA-C*03:02 | 81-88 | YSVYGETL | 0.61 | HLA-B*40:01 | 6-15 | AERPYKLHDL | 0.65 | HLA-B*40:01 | 35-43 | QELQRSEVY | 0.6 |
| HLA-B*13:01 | 2-10 | FQDPAERPY | 0.62 | HLA-C*01:02 | 81-88 | YSVYGETL | 0.67 | HLA-C*03:02 | 81-88 | YSVYGETL | 0.61 |

Continued 1 Table S2. The HLA-Ⅰ predicted epitopes of HPV-35 E6

| HPV35E6 REF | | | | HPV35E6 W78R | | | | HPV35E6 I145R | | | |
| --- | --- | --- | --- | --- | --- | --- | --- | --- | --- | --- | --- |
| Allele | Location | Peptide | PR | Allele | Location | Peptide | PR | Allele | Location | Peptide | PR |
| HLA-C*03:02 | 46-54 | ACYDLCIVY | 0.62 | HLA-A*33:03 | 70-80 | YSKISEYRRYR | 0.68 | HLA-B*13:01 | 2-10 | FQDPAERPY | 0.62 |
| HLA-A*33:03 | 69-80 | FYSKISEYRWYR | 0.63 | HLA-B*58:01 | 124-132 | RFHNIGGRW | 0.69 | HLA-C*03:02 | 46-54 | ACYDLCIVY | 0.62 |
| HLA-B*40:01 | 6-15 | AERPYKLHDL | 0.65 | HLA-A*33:03 | 68-78 | KFYSKISEYRR | 0.71 | HLA-A*33:03 | 69-80 | FYSKISEYRWYR | 0.63 |
| HLA-C*01:02 | 81-88 | YSVYGETL | 0.67 | HLA-A*02:01 | 10-19 | YKLHDLCNEV | 0.71 | HLA-B*40:01 | 6-15 | AERPYKLHDL | 0.65 |
| HLA-B*58:01 | 124-132 | RFHNIGGRW | 0.69 | HLA-A*24:02 | 42-50 | VYDFACYDL | 0.71 | HLA-C*01:02 | 81-88 | YSVYGETL | 0.67 |
| HLA-A*02:01 | 10-19 | YKLHDLCNEV | 0.71 | HLA-C*01:02 | 80-88 | RYSVYGETL | 0.72 | HLA-B*58:01 | 124-132 | RFHNIGGRW | 0.69 |
| HLA-A*24:02 | 42-50 | VYDFACYDL | 0.71 | HLA-A*11:01 | 86-94 | ETLEKQCNK | 0.74 | HLA-A*02:01 | 10-19 | YKLHDLCNEV | 0.71 |
| HLA-C*01:02 | 80-88 | RYSVYGETL | 0.72 | HLA-A*11:01 | 52-60 | IVYREGQPY | 0.75 | HLA-A*24:02 | 42-50 | VYDFACYDL | 0.71 |
| HLA-A*11:01 | 86-94 | ETLEKQCNK | 0.74 | HLA-A*33:03 | 1-8 | MFQDPAER | 0.79 | HLA-C*01:02 | 80-88 | RYSVYGETL | 0.72 |
| HLA-A*11:01 | 52-60 | IVYREGQPY | 0.75 | HLA-B*15:02 | 67-76 | LKFYSKISEY | 0.83 | HLA-A*11:01 | 86-94 | ETLEKQCNK | 0.74 |
| HLA-A*33:03 | 1-8 | MFQDPAER | 0.79 | HLA-B*15:02 | 22-32 | SIHEICLNCVY | 0.85 | HLA-A*11:01 | 52-60 | IVYREGQPY | 0.75 |
| HLA-B*15:02 | 67-76 | LKFYSKISEY | 0.83 | HLA-B*13:01 | 11-19 | KLHDLCNEV | 0.86 | HLA-A*33:03 | 1-8 | MFQDPAER | 0.79 |
| HLA-B*58:01 | 71-78 | SKISEYRW | 0.83 | HLA-B*40:01 | 17-28 | NEVEESIHEICL | 0.86 | HLA-B*15:02 | 67-76 | LKFYSKISEY | 0.83 |
| HLA-B*15:02 | 22-32 | SIHEICLNCVY | 0.85 | HLA-A*11:01 | 72-80 | KISEYRRYR | 0.87 | HLA-B*58:01 | 71-78 | SKISEYRW | 0.83 |
| HLA-B*13:01 | 11-19 | KLHDLCNEV | 0.86 | HLA-A*33:03 | 86-94 | ETLEKQCNK | 0.88 | HLA-B*15:02 | 22-32 | SIHEICLNCVY | 0.85 |
| HLA-B*58:01 | 67-78 | LKFYSKISEYRW | 0.86 | HLA-A*33:03 | 1-11 | MFQDPAERPYK | 0.89 | HLA-B*13:01 | 11-19 | KLHDLCNEV | 0.86 |
| HLA-B*40:01 | 17-28 | NEVEESIHEICL | 0.86 | HLA-B*58:01 | 132-140 | WTGRCMSCW | 0.92 | HLA-B*58:01 | 67-78 | LKFYSKISEYRW | 0.86 |
| HLA-A*33:03 | 86-94 | ETLEKQCNK | 0.88 | HLA-B*40:01 | 85-96 | GETLEKQCNKQL | 0.92 | HLA-B*40:01 | 17-28 | NEVEESIHEICL | 0.86 |
| HLA-A*33:03 | 1-11 | MFQDPAERPYK | 0.89 | HLA-A*33:03 | 70-77 | YSKISEYR | 0.95 | HLA-A*33:03 | 86-94 | ETLEKQCNK | 0.88 |
| HLA-B*58:01 | 132-140 | WTGRCMSCW | 0.92 | HLA-A*11:01 | 2-11 | FQDPAERPYK | 0.95 | HLA-A*33:03 | 1-11 | MFQDPAERPYK | 0.89 |
| HLA-B*40:01 | 85-96 | GETLEKQCNKQL | 0.92 | HLA-A*33:03 | 69-80 | FYSKISEYRRYR | 0.98 | HLA-B*58:01 | 132-140 | WTGRCMSCW | 0.92 |
| HLA-A*33:03 | 70-77 | YSKISEYR | 0.95 | HLA-B*46:01 | 37-45 | LQRSEVYDF | 0.98 | HLA-B*40:01 | 85-96 | GETLEKQCNKQL | 0.92 |
| HLA-A*11:01 | 2-11 | FQDPAERPYK | 0.95 | HLA-A*24:02 | 1-12 | MFQDPAERPYKL | 0.98 | HLA-A*33:03 | 70-77 | YSKISEYR | 0.95 |
| HLA-B*46:01 | 37-45 | LQRSEVYDF | 0.98 | HLA-A*33:03 | 46-55 | ACYDLCIVYR | 1 | HLA-A*11:01 | 2-11 | FQDPAERPYK | 0.95 |
| HLA-A*24:02 | 1-12 | MFQDPAERPYKL | 0.98 |  |  |  |  | HLA-B*46:01 | 37-45 | LQRSEVYDF | 0.98 |
| HLA-A*33:03 | 46-55 | ACYDLCIVYR | 1 |  |  |  |  | HLA-A*24:02 | 1-12 | MFQDPAERPYKL | 0.98 |
|  |  |  |  |  |  |  |  | HLA-A*33:03 | 46-55 | ACYDLCIVYR | 1 |

Table S3. The HLA-Ⅰ predicted epitopes of HPV-35 E7

| HPV35E7 REF | | | | HPV35E7 H23Y | | | | HPV35E7 L28F | | | |
| --- | --- | --- | --- | --- | --- | --- | --- | --- | --- | --- | --- |
| allele | Location | peptide | PR | allele | Location | peptide | PR | allele | Location | peptide | PR |
| HLA-B*15:02 | 45-53 | QAKPDTSNY | 0.03 | HLA-B*15:02 | 45-53 | QAKPDTSNY | 0.03 | HLA-B*15:02 | 45-53 | QAKPDTSNY | 0.03 |
| HLA-B*46:01 | 45-53 | QAKPDTSNY | 0.05 | HLA-B*46:01 | 45-53 | QAKPDTSNY | 0.05 | HLA-B*46:01 | 45-53 | QAKPDTSNY | 0.05 |
| HLA-C*03:02 | 45-53 | QAKPDTSNY | 0.06 | HLA-C*03:02 | 45-53 | QAKPDTSNY | 0.06 | HLA-C*03:02 | 45-53 | QAKPDTSNY | 0.06 |
| HLA-A*02:01 | 7-15 | TLQDYVLDL | 0.09 | HLA-A*02:01 | 7-15 | TLQDYVLDL | 0.09 | HLA-A*02:01 | 7-15 | TLQDYVLDL | 0.09 |
| HLA-B*40:01 | 3-13 | GEITTLQDYVL | 0.1 | HLA-B*40:01 | 3-13 | GEITTLQDYVL | 0.1 | HLA-B*40:01 | 3-13 | GEITTLQDYVL | 0.1 |
| HLA-A*02:01 | 11-19 | YVLDLEPEA | 0.26 | HLA-A*02:01 | 11-19 | YVLDLEPEA | 0.26 | HLA-A*02:01 | 11-19 | YVLDLEPEA | 0.26 |
| HLA-B*15:02 | 44-53 | GQAKPDTSNY | 0.29 | HLA-B*15:02 | 44-53 | GQAKPDTSNY | 0.29 | HLA-B*15:02 | 44-53 | GQAKPDTSNY | 0.29 |
| HLA-A*33:03 | 90-98 | IVCPGCSQR | 0.32 | HLA-A*33:03 | 90-98 | IVCPGCSQR | 0.32 | HLA-A*33:03 | 90-98 | IVCPGCSQR | 0.32 |
| HLA-A*11:01 | 38-47 | TIDGPAGQAK | 0.4 | HLA-A*11:01 | 38-47 | TIDGPAGQAK | 0.4 | HLA-A*11:01 | 38-47 | TIDGPAGQAK | 0.4 |
| HLA-A*11:01 | 71-79 | QSTHIDIRK | 0.46 | HLA-A*11:01 | 71-79 | QSTHIDIRK | 0.46 | HLA-A*11:01 | 71-79 | QSTHIDIRK | 0.46 |
| HLA-B*46:01 | 44-53 | GQAKPDTSNY | 0.46 | HLA-B*46:01 | 44-53 | GQAKPDTSNY | 0.46 | HLA-B*46:01 | 44-53 | GQAKPDTSNY | 0.46 |
| HLA-B*40:01 | 80-88 | LEDLLMGTF | 0.47 | HLA-B*40:01 | 80-88 | LEDLLMGTF | 0.47 | HLA-B*40:01 | 80-88 | LEDLLMGTF | 0.47 |
| HLA-B*46:01 | 43-53 | AGQAKPDTSNY | 0.5 | HLA-B*46:01 | 43-53 | AGQAKPDTSNY | 0.5 | HLA-B*46:01 | 43-53 | AGQAKPDTSNY | 0.5 |
| HLA-B*58:01 | 45-53 | QAKPDTSNY | 0.58 | HLA-B*58:01 | 45-53 | QAKPDTSNY | 0.58 | HLA-B*58:01 | 45-53 | QAKPDTSNY | 0.58 |
| HLA-C*03:04 | 72-80 | STHIDIRKL | 0.58 | HLA-C*03:04 | 72-80 | STHIDIRKL | 0.58 | HLA-C*03:04 | 72-80 | STHIDIRKL | 0.58 |
| HLA-B*13:01 | 79-88 | KLEDLLMGTF | 0.66 | HLA-B*13:01 | 79-88 | KLEDLLMGTF | 0.66 | HLA-B*13:01 | 79-88 | KLEDLLMGTF | 0.66 |
| HLA-C*08:01 | 7-15 | TLQDYVLDL | 0.69 | HLA-C*08:01 | 7-15 | TLQDYVLDL | 0.69 | HLA-C*08:01 | 7-15 | TLQDYVLDL | 0.69 |
| HLA-C*03:04 | 5-13 | ITTLQDYVL | 0.75 | HLA-C*03:04 | 5-13 | ITTLQDYVL | 0.75 | HLA-C*03:04 | 5-13 | ITTLQDYVL | 0.75 |
| HLA-B*13:01 | 7-15 | TLQDYVLDL | 0.76 | HLA-B*13:01 | 7-15 | TLQDYVLDL | 0.76 | HLA-B*13:01 | 7-15 | TLQDYVLDL | 0.76 |
| HLA-C*08:01 | 11-19 | YVLDLEPEA | 0.76 | HLA-C*08:01 | 11-19 | YVLDLEPEA | 0.76 | HLA-C*08:01 | 11-19 | YVLDLEPEA | 0.76 |
| HLA-A*11:01 | 70-79 | VQSTHIDIRK | 0.79 | HLA-A*11:01 | 70-79 | VQSTHIDIRK | 0.79 | HLA-A*11:01 | 70-79 | VQSTHIDIRK | 0.79 |
| HLA-C*08:01 | 47-55 | KPDTSNYNI | 0.86 | HLA-B*15:02 | 14-23 | DLEPEATDLY | 0.85 | HLA-C*08:01 | 47-55 | KPDTSNYNI | 0.86 |
| HLA-B*40:01 | 3-11 | GEITTLQDY | 0.87 | HLA-C*08:01 | 47-55 | KPDTSNYNI | 0.86 | HLA-B*40:01 | 3-11 | GEITTLQDY | 0.87 |
| HLA-C*08:01 | 5-13 | ITTLQDYVL | 0.91 | HLA-B*40:01 | 3-11 | GEITTLQDY | 0.87 | HLA-C*08:01 | 5-13 | ITTLQDYVL | 0.91 |
| HLA-C*03:02 | 72-80 | STHIDIRKL | 0.94 | HLA-C*08:01 | 5-13 | ITTLQDYVL | 0.91 | HLA-C*03:02 | 72-80 | STHIDIRKL | 0.94 |
| HLA-A*02:01 | 79-87 | KLEDLLMGT | 0.95 | HLA-C*03:02 | 72-80 | STHIDIRKL | 0.94 | HLA-A*02:01 | 79-87 | KLEDLLMGT | 0.95 |
| HLA-C*01:02 | 7-15 | TLQDYVLDL | 1 | HLA-A*02:01 | 79-87 | KLEDLLMGT | 0.95 | HLA-C*01:02 | 7-15 | TLQDYVLDL | 1 |
|  |  |  |  | HLA-C*01:02 | 7-15 | TLQDYVLDL | 1 |  |  |  |  |

Table S4. The HLA-Ⅱ predicted epitopes of HPV-35 E6

| HPV35E6 REF | | | | | HPV35E6 W78R | | | | | HPV35E6 I145R | | | | |
| --- | --- | --- | --- | --- | --- | --- | --- | --- | --- | --- | --- | --- | --- | --- |
| Allele | Location | Core_peptide | Peptide | Rank | Allele | Location | Core_peptide | Peptide | Rank | Allele | Location | Core_peptide | Peptide | Rank |
| HLA-DPA1*01:03/DPB1*04:01 | 77-88 | YRYSVYGET | RWYRYSVYGETL | 0.42 | HLA-DPA1*01:03/DPB1*04:01 | 77-88 | YRYSVYGET | RRYRYSVYGETL | 0.18 | HLA-DPA1*01:03/DPB1*04:01 | 77-88 | YRYSVYGET | RWYRYSVYGETL | 0.42 |
| HLA-DPA1*01:03/DPB1*04:01 | 76-87 | YRYSVYGET | YRWYRYSVYGET | 0.42 | HLA-DPA1*01:03/DPB1*04:01 | 76-87 | YRYSVYGET | YRRYRYSVYGET | 0.18 | HLA-DPA1*01:03/DPB1*04:01 | 76-87 | YRYSVYGET | YRWYRYSVYGET | 0.42 |
| HLA-DPA1*01:03/DPB1*04:01 | 76-89 | YRYSVYGET | YRWYRYSVYGETLE | 0.45 | HLA-DPA1*01:03/DPB1*04:01 | 75-90 | YRYSVYGET | EYRRYRYSVYGETLEK | 0.19 | HLA-DPA1*01:03/DPB1*04:01 | 76-89 | YRYSVYGET | YRWYRYSVYGETLE | 0.45 |
| HLA-DPA1*01:03/DPB1*04:01 | 76-88 | YRYSVYGET | YRWYRYSVYGETL | 0.48 | HLA-DPA1*01:03/DPB1*04:01 | 76-88 | YRYSVYGET | YRRYRYSVYGETL | 0.23 | HLA-DPA1*01:03/DPB1*04:01 | 76-88 | YRYSVYGET | YRWYRYSVYGETL | 0.48 |
| HLA-DPA1*01:03/DPB1*04:01 | 77-89 | YRYSVYGET | RWYRYSVYGETLE | 0.49 | HLA-DPA1*01:03/DPB1*04:01 | 76-90 | YRYSVYGET | YRRYRYSVYGETLEK | 0.23 | HLA-DPA1*01:03/DPB1*04:01 | 77-89 | YRYSVYGET | RWYRYSVYGETLE | 0.49 |
| HLA-DPA1*01:03/DPB1*04:01 | 77-90 | YRYSVYGET | RWYRYSVYGETLEK | 0.5 | HLA-DPA1*01:03/DPB1*04:01 | 77-89 | YRYSVYGET | RRYRYSVYGETLE | 0.24 | HLA-DPA1*01:03/DPB1*04:01 | 77-90 | YRYSVYGET | RWYRYSVYGETLEK | 0.5 |
| HLA-DPA1*01:03/DPB1*04:01 | 76-90 | YRYSVYGET | YRWYRYSVYGETLEK | 0.5 | HLA-DPA1*01:03/DPB1*04:01 | 76-89 | YRYSVYGET | YRRYRYSVYGETLE | 0.26 | HLA-DPA1*01:03/DPB1*04:01 | 76-90 | YRYSVYGET | YRWYRYSVYGETLEK | 0.5 |
| HLA-DPA1*01:03/DPB1*04:01 | 75-90 | YRYSVYGET | EYRWYRYSVYGETLEK | 0.55 | HLA-DPA1*01:03/DPB1*04:01 | 77-90 | YRYSVYGET | RRYRYSVYGETLEK | 0.28 | HLA-DPA1*01:03/DPB1*04:01 | 75-90 | YRYSVYGET | EYRWYRYSVYGETLEK | 0.55 |
| HLA-DPA1*01:03/DPB1*04:01 | 74-90 | YRYSVYGET | SEYRWYRYSVYGETLEK | 0.64 | HLA-DPA1*01:03/DPB1*04:01 | 74-90 | YRYSVYGET | SEYRRYRYSVYGETLEK | 0.32 | HLA-DPA1*01:03/DPB1*04:01 | 74-90 | YRYSVYGET | SEYRWYRYSVYGETLEK | 0.64 |
| HLA-DPA1*01:03/DPB1*04:01 | 75-89 | YRYSVYGET | EYRWYRYSVYGETLE | 0.76 | HLA-DPA1*01:03/DPB1*04:01 | 73-90 | YRYSVYGET | ISEYRRYRYSVYGETLEK | 0.33 | HLA-DPA1*01:03/DPB1*04:01 | 75-89 | YRYSVYGET | EYRWYRYSVYGETLE | 0.76 |
| HLA-DPA1*01:03/DPB1*04:01 | 73-90 | YRYSVYGET | ISEYRWYRYSVYGETLEK | 0.78 | HLA-DPA1*01:03/DPB1*04:01 | 76-91 | YRYSVYGET | YRRYRYSVYGETLEKQ | 0.33 | HLA-DPA1*01:03/DPB1*04:01 | 73-90 | YRYSVYGET | ISEYRWYRYSVYGETLEK | 0.78 |
| HLA-DPA1*01:03/DPB1*04:01 | 76-91 | YRYSVYGET | YRWYRYSVYGETLEKQ | 0.81 | HLA-DPA1*01:03/DPB1*04:01 | 75-91 | YRYSVYGET | EYRRYRYSVYGETLEKQ | 0.36 | HLA-DPA1*01:03/DPB1*04:01 | 76-91 | YRYSVYGET | YRWYRYSVYGETLEKQ | 0.81 |
| HLA-DPA1*01:03/DPB1*04:01 | 75-91 | YRYSVYGET | EYRWYRYSVYGETLEKQ | 0.84 | HLA-DPA1*01:03/DPB1*04:01 | 75-89 | YRYSVYGET | EYRRYRYSVYGETLE | 0.38 | HLA-DPA1*01:03/DPB1*04:01 | 75-91 | YRYSVYGET | EYRWYRYSVYGETLEKQ | 0.84 |
| HLA-DPA1*01:03/DPB1*04:01 | 74-91 | YRYSVYGET | SEYRWYRYSVYGETLEKQ | 0.88 | HLA-DPA1*01:03/DPB1*04:01 | 75-87 | YRYSVYGET | EYRRYRYSVYGET | 0.39 | HLA-DPA1*01:03/DPB1*04:01 | 74-91 | YRYSVYGET | SEYRWYRYSVYGETLEKQ | 0.88 |
| HLA-DPA1*01:03/DPB1*04:01 | 75-87 | YRYSVYGET | EYRWYRYSVYGET | 0.89 | HLA-DPA1*01:03/DPB1*04:01 | 75-88 | YRYSVYGET | EYRRYRYSVYGETL | 0.39 | HLA-DPA1*01:03/DPB1*04:01 | 75-87 | YRYSVYGET | EYRWYRYSVYGET | 0.89 |
| HLA-DPA1*01:03/DPB1*04:01 | 75-88 | YRYSVYGET | EYRWYRYSVYGETL | 0.94 | HLA-DPA1*01:03/DPB1*04:01 | 74-91 | YRYSVYGET | SEYRRYRYSVYGETLEKQ | 0.43 | HLA-DPA1*01:03/DPB1*04:01 | 75-88 | YRYSVYGET | EYRWYRYSVYGETL | 0.94 |
| HLA-DRB1*12:02 | 50-61 | IVYREGQPY | LCIVYREGQPYG | 0.95 | HLA-DPA1*01:03/DPB1*04:01 | 74-89 | YRYSVYGET | SEYRRYRYSVYGETLE | 0.47 | HLA-DRB1*12:02 | 50-61 | IVYREGQPY | LCIVYREGQPYG | 0.95 |
| HLA-DPA1*01:03/DPB1*04:01 | 75-92 | YRYSVYGET | EYRWYRYSVYGETLEKQC | 1.2 | HLA-DPA1*01:03/DPB1*04:01 | 75-92 | YRYSVYGET | EYRRYRYSVYGETLEKQC | 0.54 | HLA-DPA1*01:03/DPB1*04:01 | 75-92 | YRYSVYGET | EYRWYRYSVYGETLEKQC | 1.2 |
| HLA-DPA1*01:03/DPB1*04:01 | 77-91 | YRYSVYGET | RWYRYSVYGETLEKQ | 1.2 | HLA-DPA1*01:03/DPB1*04:01 | 77-91 | YRYSVYGET | RRYRYSVYGETLEKQ | 0.56 | HLA-DPA1*01:03/DPB1*04:01 | 77-91 | YRYSVYGET | RWYRYSVYGETLEKQ | 1.2 |
| HLA-DPA1*01:03/DPB1*04:01 | 76-92 | YRYSVYGET | YRWYRYSVYGETLEKQC | 1.2 | HLA-DPA1*01:03/DPB1*04:01 | 76-92 | YRYSVYGET | YRRYRYSVYGETLEKQC | 0.56 | HLA-DPA1*01:03/DPB1*04:01 | 76-92 | YRYSVYGET | YRWYRYSVYGETLEKQC | 1.2 |
| HLA-DPA1*01:03/DPB1*04:01 | 74-89 | YRYSVYGET | SEYRWYRYSVYGETLE | 1.2 | HLA-DPA1*01:03/DPB1*04:01 | 78-89 | YRYSVYGET | RYRYSVYGETLE | 0.63 | HLA-DPA1*01:03/DPB1*04:01 | 74-89 | YRYSVYGET | SEYRWYRYSVYGETLE | 1.2 |
| HLA-DRB1*12:02 | 49-61 | IVYREGQPY | DLCIVYREGQPYG | 1.3 | HLA-DPA1*01:03/DPB1*04:01 | 73-89 | YRYSVYGET | ISEYRRYRYSVYGETLE | 0.63 | HLA-DRB1*12:02 | 49-61 | IVYREGQPY | DLCIVYREGQPYG | 1.3 |
| HLA-DPA1*01:03/DPB1*04:01 | 73-89 | YRYSVYGET | ISEYRWYRYSVYGETLE | 1.4 | HLA-DPA1*01:03/DPB1*04:01 | 74-88 | YRYSVYGET | SEYRRYRYSVYGETL | 0.74 | HLA-DPA1*01:03/DPB1*04:01 | 73-89 | YRYSVYGET | ISEYRWYRYSVYGETLE | 1.4 |
| HLA-DRB1*12:02 | 49-60 | IVYREGQPY | DLCIVYREGQPY | 1.5 | HLA-DPA1*01:03/DPB1*04:01 | 72-89 | YRYSVYGET | KISEYRRYRYSVYGETLE | 0.8 | HLA-DRB1*12:02 | 49-60 | IVYREGQPY | DLCIVYREGQPY | 1.5 |
| HLA-DPA1*01:03/DPB1*04:01 | 74-88 | YRYSVYGET | SEYRWYRYSVYGETL | 1.5 | HLA-DPA1*01:03/DPB1*04:01 | 76-93 | YRYSVYGET | YRRYRYSVYGETLEKQCN | 0.8 | HLA-DPA1*01:03/DPB1*04:01 | 74-88 | YRYSVYGET | SEYRWYRYSVYGETL | 1.5 |
| HLA-DPA1*01:03/DPB1*04:01 | 78-89 | YRYSVYGET | WYRYSVYGETLE | 1.6 | HLA-DPA1*01:03/DPB1*04:01 | 78-90 | YRYSVYGET | RYRYSVYGETLEK | 0.81 | HLA-DPA1*01:03/DPB1*04:01 | 78-89 | YRYSVYGET | WYRYSVYGETLE | 1.6 |
| HLA-DRB1*15:02 | 79-90 | YSVYGETLE | YRYSVYGETLEK | 1.6 | HLA-DPA1*01:03/DPB1*04:01 | 74-87 | YRYSVYGET | SEYRRYRYSVYGET | 0.91 | HLA-DRB1*15:02 | 79-90 | YSVYGETLE | YRYSVYGETLEK | 1.6 |
| HLA-DPA1*01:03/DPB1*04:01 | 76-93 | YRYSVYGET | YRWYRYSVYGETLEKQCN | 1.6 | HLA-DRB1*12:02 | 50-61 | IVYREGQPY | LCIVYREGQPYG | 0.95 | HLA-DPA1*01:03/DPB1*04:01 | 76-93 | YRYSVYGET | YRWYRYSVYGETLEKQCN | 1.6 |
| HLA-DPA1*01:03/DPB1*04:01 | 72-89 | YRYSVYGET | KISEYRWYRYSVYGETLE | 1.7 | HLA-DPA1*01:03/DPB1*04:01 | 77-92 | YRYSVYGET | RRYRYSVYGETLEKQC | 0.97 | HLA-DPA1*01:03/DPB1*04:01 | 72-89 | YRYSVYGET | KISEYRWYRYSVYGETLE | 1.7 |
| HLA-DRB1*12:02 | 49-62 | IVYREGQPY | DLCIVYREGQPYGV | 1.7 | HLA-DPA1*01:03/DPB1*04:01 | 73-88 | YRYSVYGET | ISEYRRYRYSVYGETL | 1.2 | HLA-DRB1*12:02 | 49-62 | IVYREGQPY | DLCIVYREGQPYGV | 1.7 |
| HLA-DPA1*01:03/DPB1*04:01 | 78-90 | YRYSVYGET | WYRYSVYGETLEK | 1.8 | HLA-DRB1*12:02 | 49-61 | IVYREGQPY | DLCIVYREGQPYG | 1.3 | HLA-DPA1*01:03/DPB1*04:01 | 78-90 | YRYSVYGET | WYRYSVYGETLEK | 1.8 |
| HLA-DPA1*01:03/DPB1*04:01 | 74-87 | YRYSVYGET | SEYRWYRYSVYGET | 1.8 | HLA-DRB1*12:02 | 49-60 | IVYREGQPY | DLCIVYREGQPY | 1.5 | HLA-DPA1*01:03/DPB1*04:01 | 74-87 | YRYSVYGET | SEYRWYRYSVYGET | 1.8 |
| HLA-DPA1*01:03/DPB1*04:01 | 77-92 | YRYSVYGET | RWYRYSVYGETLEKQC | 1.8 | HLA-DPA1*01:03/DPB1*04:01 | 77-93 | YRYSVYGET | RRYRYSVYGETLEKQCN | 1.5 | HLA-DPA1*01:03/DPB1*04:01 | 77-92 | YRYSVYGET | RWYRYSVYGETLEKQC | 1.8 |
| HLA-DRB1*15:02 | 76-87 | WYRYSVYGE | YRWYRYSVYGET | 2 | HLA-DPA1*01:03/DPB1*04:01 | 72-88 | YRYSVYGET | KISEYRRYRYSVYGETL | 1.5 | HLA-DRB1*15:02 | 76-87 | WYRYSVYGE | YRWYRYSVYGET | 2 |
| HLA-DPA1*01:03/DPB1*04:01 | 73-88 | YRYSVYGET | ISEYRWYRYSVYGETL | 2 | HLA-DRB1*15:02 | 79-90 | YSVYGETLE | YRYSVYGETLEK | 1.6 | HLA-DPA1*01:03/DPB1*04:01 | 73-88 | YRYSVYGET | ISEYRWYRYSVYGETL | 2 |
| HLA-DRB1*12:02 | 51-62 | IVYREGQPY | CIVYREGQPYGV | 2.1 | HLA-DPA1*01:03/DPB1*04:01 | 73-87 | YRYSVYGET | ISEYRRYRYSVYGET | 1.6 | HLA-DRB1*12:02 | 51-62 | IVYREGQPY | CIVYREGQPYGV | 2.1 |
| HLA-DRB1*12:02 | 50-62 | IVYREGQPY | LCIVYREGQPYGV | 2.1 | HLA-DRB1*15:02 | 78-91 | YSVYGETLE | RYRYSVYGETLEKQ | 1.6 | HLA-DRB1*12:02 | 50-62 | IVYREGQPY | LCIVYREGQPYGV | 2.1 |
| HLA-DRB1*12:02 | 48-61 | IVYREGQPY | YDLCIVYREGQPYG | 2.1 | HLA-DRB1*15:02 | 78-90 | YSVYGETLE | RYRYSVYGETLEK | 1.7 | HLA-DRB1*12:02 | 48-61 | IVYREGQPY | YDLCIVYREGQPYG | 2.1 |
| HLA-DRB1*15:02 | 79-91 | YSVYGETLE | YRYSVYGETLEKQ | 2.2 | HLA-DRB1*12:02 | 49-62 | IVYREGQPY | DLCIVYREGQPYGV | 1.7 | HLA-DRB1*15:02 | 79-91 | YSVYGETLE | YRYSVYGETLEKQ | 2.2 |
| HLA-DRB1*15:02 | 75-87 | WYRYSVYGE | EYRWYRYSVYGET | 2.2 | HLA-DPA1*01:03/DPB1*04:01 | 71-88 | YRYSVYGET | SKISEYRRYRYSVYGETL | 1.9 | HLA-DRB1*15:02 | 75-87 | WYRYSVYGE | EYRWYRYSVYGET | 2.2 |
| HLA-DRB1*15:02 | 122-134 | FHNIGGRWT | KKRFHNIGGRWTG | 2.2 | HLA-DRB1*12:02 | 71-82 | ISEYRRYRY | SKISEYRRYRYS | 1.9 | HLA-DRB1*15:02 | 122-134 | FHNIGGRWT | KKRFHNIGGRWTG | 2.2 |
| HLA-DRB1*15:02 | 122-135 | FHNIGGRWT | KKRFHNIGGRWTGR | 2.2 | HLA-DPA1*01:03/DPB1*04:01 | 77-94 | YRYSVYGET | RRYRYSVYGETLEKQCNK | 2 | HLA-DRB1*15:02 | 122-135 | FHNIGGRWT | KKRFHNIGGRWTGR | 2.2 |
| HLA-DQA1*01:01/DQB1*05:01 | 79-90 | YSVYGETLE | YRYSVYGETLEK | 2.3 | HLA-DPA1*01:03/DPB1*04:01 | 78-91 | YRYSVYGET | RYRYSVYGETLEKQ | 2 | HLA-DQA1*01:01/DQB1*05:01 | 79-90 | YSVYGETLE | YRYSVYGETLEK | 2.3 |
| HLA-DRB1*15:02 | 75-86 | WYRYSVYGE | EYRWYRYSVYGE | 2.4 | HLA-DRB1*12:02 | 51-62 | IVYREGQPY | CIVYREGQPYGV | 2.1 | HLA-DRB1*15:02 | 75-86 | WYRYSVYGE | EYRWYRYSVYGE | 2.4 |
| HLA-DRB1*12:02 | 48-62 | IVYREGQPY | YDLCIVYREGQPYGV | 2.5 | HLA-DRB1*12:02 | 50-62 | IVYREGQPY | LCIVYREGQPYGV | 2.1 | HLA-DRB1*12:02 | 48-62 | IVYREGQPY | YDLCIVYREGQPYGV | 2.5 |
| HLA-DPA1*01:03/DPB1*04:01 | 77-93 | YRYSVYGET | RWYRYSVYGETLEKQCN | 2.6 | HLA-DRB1*12:02 | 48-61 | IVYREGQPY | YDLCIVYREGQPYG | 2.1 | HLA-DPA1*01:03/DPB1*04:01 | 77-93 | YRYSVYGET | RWYRYSVYGETLEKQCN | 2.6 |
| HLA-DPA1*01:03/DPB1*04:01 | 72-88 | YRYSVYGET | KISEYRWYRYSVYGETL | 2.6 | HLA-DRB1*15:02 | 79-91 | YSVYGETLE | YRYSVYGETLEKQ | 2.2 | HLA-DPA1*01:03/DPB1*04:01 | 72-88 | YRYSVYGET | KISEYRWYRYSVYGETL | 2.6 |
| HLA-DRB1*15:02 | 80-91 | YSVYGETLE | RYSVYGETLEKQ | 2.7 | HLA-DRB1*15:02 | 122-134 | FHNIGGRWT | KKRFHNIGGRWTG | 2.2 | HLA-DRB1*15:02 | 80-91 | YSVYGETLE | RYSVYGETLEKQ | 2.7 |
| HLA-DRB1*15:02 | 123-134 | FHNIGGRWT | KRFHNIGGRWTG | 2.8 | HLA-DRB1*15:02 | 122-135 | FHNIGGRWT | KKRFHNIGGRWTGR | 2.2 | HLA-DRB1*15:02 | 123-134 | FHNIGGRWT | KRFHNIGGRWTG | 2.8 |
| HLA-DPA1*01:03/DPB1*04:01 | 73-87 | YRYSVYGET | ISEYRWYRYSVYGET | 2.8 | HLA-DQA1*01:01/DQB1*05:01 | 79-90 | YSVYGETLE | YRYSVYGETLEK | 2.3 | HLA-DPA1*01:03/DPB1*04:01 | 73-87 | YRYSVYGET | ISEYRWYRYSVYGET |  |
| HLA-DRB1*15:02 | 121-135 | FHNIGGRWT | EKKRFHNIGGRWTGR | 2.9 | HLA-DPA1*01:03/DPB1*04:01 | 72-87 | YRYSVYGET | KISEYRRYRYSVYGET | 2.3 | HLA-DRB1*15:02 | 121-135 | FHNIGGRWT | EKKRFHNIGGRWTGR |  |
| HLA-DPA1*01:03/DPB1*04:01 | 77-94 | YRYSVYGET | RWYRYSVYGETLEKQCNK | 3.3 | HLA-DRB1*15:02 | 77-91 | YSVYGETLE | RRYRYSVYGETLEKQ | 2.4 | HLA-DPA1*01:03/DPB1*04:01 | 77-94 | YRYSVYGET | RWYRYSVYGETLEKQCNK |  |
| HLA-DPA1*01:03/DPB1*04:01 | 71-88 | YRYSVYGET | SKISEYRWYRYSVYGETL | 3.3 | HLA-DRB1*12:02 | 48-62 | IVYREGQPY | YDLCIVYREGQPYGV | 2.5 | HLA-DPA1*01:03/DPB1*04:01 | 71-88 | YRYSVYGET | SKISEYRWYRYSVYGETL |  |
| HLA-DRB1*12:02 | 47-62 | IVYREGQPY | CYDLCIVYREGQPYGV | 3.3 | HLA-DQA1*01:01/DQB1*05:01 | 78-89 | YSVYGETLE | RYRYSVYGETLE | 2.7 | HLA-DRB1*12:02 | 47-62 | IVYREGQPY | CYDLCIVYREGQPYGV |  |
| HLA-DRB1*15:02 | 74-85 | YRWYRYSVY | SEYRWYRYSVYG | 3.5 | HLA-DRB1*15:02 | 80-91 | YSVYGETLE | RYSVYGETLEKQ | 2.7 | HLA-DRB1*15:02 | 74-85 | YRWYRYSVY | SEYRWYRYSVYG |  |
| HLA-DRB1*15:02 | 123-135 | FHNIGGRWT | KRFHNIGGRWTGR | 3.5 | HLA-DRB1*15:02 | 123-134 | FHNIGGRWT | KRFHNIGGRWTG | 2.8 | HLA-DRB1*15:02 | 123-135 | FHNIGGRWT | KRFHNIGGRWTGR |  |
| HLA-DRB1*15:02 | 74-87 | WYRYSVYGE | SEYRWYRYSVYGET | 3.5 | HLA-DRB1*15:02 | 121-135 | FHNIGGRWT | EKKRFHNIGGRWTGR | 2.9 | HLA-DRB1*15:02 | 74-87 | WYRYSVYGE | SEYRWYRYSVYGET |  |
| HLA-DRB1*15:02 | 78-90 | YSVYGETLE | WYRYSVYGETLEK | 3.6 | HLA-DPA1*01:03/DPB1*04:01 | 71-87 | YRYSVYGET | SKISEYRRYRYSVYGET | 3 | HLA-DRB1*15:02 | 78-90 | YSVYGETLE | WYRYSVYGETLEK |  |
| HLA-DRB1*15:02 | 78-91 | YSVYGETLE | WYRYSVYGETLEKQ | 3.6 | HLA-DRB1*15:02 | 68-79 | YSKISEYRR | KFYSKISEYRRY | 3.1 | HLA-DRB1*15:02 | 78-91 | YSVYGETLE | WYRYSVYGETLEKQ |  |
| HLA-DRB1*12:02 | 47-61 | IVYREGQPY | CYDLCIVYREGQPYG | 3.7 | HLA-DRB1*15:02 | 77-90 | YSVYGETLE | RRYRYSVYGETLEK | 3.1 | HLA-DRB1*12:02 | 47-61 | IVYREGQPY | CYDLCIVYREGQPYG |  |
| HLA-DPA1*01:03/DPB1*04:01 | 72-87 | YRYSVYGET | KISEYRWYRYSVYGET | 3.8 | HLA-DRB1*15:02 | 76-91 | YSVYGETLE | YRRYRYSVYGETLEKQ | 3.1 | HLA-DPA1*01:03/DPB1*04:01 | 72-87 | YRYSVYGET | KISEYRWYRYSVYGET |  |
| HLA-DRB1*12:02 | 48-60 | IVYREGQPY | YDLCIVYREGQPY | 3.8 | HLA-DRB1*12:02 | 47-62 | IVYREGQPY | CYDLCIVYREGQPYGV | 3.3 | HLA-DRB1*12:02 | 48-60 | IVYREGQPY | YDLCIVYREGQPY |  |
| HLA-DRB1*15:02 | 75-88 | WYRYSVYGE | EYRWYRYSVYGETL | 3.8 | HLA-DRB1*15:02 | 67-78 | FYSKISEYR | LKFYSKISEYRR | 3.5 | HLA-DRB1*15:02 | 75-88 | WYRYSVYGE | EYRWYRYSVYGETL |  |
| HLA-DRB1*15:02 | 75-89 | WYRYSVYGE | EYRWYRYSVYGETLE | 3.8 | HLA-DRB1*15:02 | 74-85 | YRRYRYSVY | SEYRRYRYSVYG | 3.5 | HLA-DRB1*15:02 | 75-89 | WYRYSVYGE | EYRWYRYSVYGETLE |  |
| HLA-DQA1*01:01/DQB1*05:01 | 75-86 | WYRYSVYGE | EYRWYRYSVYGE | 3.9 | HLA-DRB1*15:02 | 123-135 | FHNIGGRWT | KRFHNIGGRWTGR | 3.5 | HLA-DQA1*01:01/DQB1*05:01 | 75-86 | WYRYSVYGE | EYRWYRYSVYGE |  |
| HLA-DQA1*01:01/DQB1*05:01 | 75-87 | WYRYSVYGE | EYRWYRYSVYGET | 3.9 | HLA-DRB1*15:02 | 78-89 | YSVYGETLE | RYRYSVYGETLE | 3.6 | HLA-DQA1*01:01/DQB1*05:01 | 75-87 | WYRYSVYGE | EYRWYRYSVYGET |  |
| HLA-DPA1*01:03/DPB1*04:01 | 78-91 | YRYSVYGET | WYRYSVYGETLEKQ | 3.9 | HLA-DRB1*12:02 | 70-81 | ISEYRRYRY | YSKISEYRRYRY | 3.7 | HLA-DPA1*01:03/DPB1*04:01 | 78-91 | YRYSVYGET | WYRYSVYGETLEKQ |  |

Continued 1 Table S4. The HLA-Ⅱ predicted epitopes of HPV-35 E6

| HPV35E6 REF | | | | | HPV35E6 W78R | | | | | HPV35E6 I145R | | | | |
| --- | --- | --- | --- | --- | --- | --- | --- | --- | --- | --- | --- | --- | --- | --- |
| Allele | Location | Core_peptide | Peptide | Rank | Allele | Location | Core_peptide | Peptide | Rank | Allele | Location | Core_peptide | Peptide | Rank |
| HLA-DRB1*15:02 | 120-135 | FHNIGGRWT | EEKKRFHNIGGRWTGR | 3.9 | HLA-DRB1*12:02 | 47-61 | IVYREGQPY | CYDLCIVYREGQPYG | 3.7 | HLA-DRB1*15:02 | 120-135 | FHNIGGRWT | EEKKRFHNIGGRWTGR | 3.9 |
| HLA-DRB1*15:02 | 74-86 | YRWYRYSVY | SEYRWYRYSVYGE | 4 | HLA-DPA1*01:03/DPB1*04:01 | 70-87 | YRYSVYGET | YSKISEYRRYRYSVYGET | 3.8 | HLA-DRB1*15:02 | 74-86 | YRWYRYSVY | SEYRWYRYSVYGE | 4 |
| HLA-DRB1*15:02 | 122-133 | FHNIGGRWT | KKRFHNIGGRWT | 4.1 | HLA-DRB1*12:02 | 48-60 | IVYREGQPY | YDLCIVYREGQPY | 3.8 | HLA-DRB1*15:02 | 122-133 | FHNIGGRWT | KKRFHNIGGRWT | 4.1 |
| HLA-DRB1*15:02 | 121-134 | FHNIGGRWT | EKKRFHNIGGRWTG | 4.1 | HLA-DRB1*12:02 | 70-82 | ISEYRRYRY | YSKISEYRRYRYS | 3.9 | HLA-DRB1*15:02 | 121-134 | FHNIGGRWT | EKKRFHNIGGRWTG | 4.1 |
| HLA-DRB1*12:02 | 46-62 | IVYREGQPY | ACYDLCIVYREGQPYGV | 4.1 | HLA-DRB1*15:02 | 120-135 | FHNIGGRWT | EEKKRFHNIGGRWTGR | 3.9 | HLA-DRB1*12:02 | 46-62 | IVYREGQPY | ACYDLCIVYREGQPYGV | 4.1 |
| HLA-DRB1*15:02 | 74-89 | WYRYSVYGE | SEYRWYRYSVYGETLE | 4.2 | HLA-DPA1*01:03/DPB1*04:01 | 78-92 | YRYSVYGET | RYRYSVYGETLEKQC | 4 | HLA-DRB1*15:02 | 74-89 | WYRYSVYGE | SEYRWYRYSVYGETLE | 4.2 |
| HLA-DQA1*01:01/DQB1*05:01 | 78-89 | YSVYGETLE | WYRYSVYGETLE | 4.6 | HLA-DRB1*15:02 | 75-91 | YSVYGETLE | EYRRYRYSVYGETLEKQ | 4 | HLA-DQA1*01:01/DQB1*05:01 | 78-89 | YSVYGETLE | WYRYSVYGETLE | 4.6 |
| HLA-DPA1*01:03/DPB1*04:01 | 74-85 | YRWYRYSVY | SEYRWYRYSVYG | 4.6 | HLA-DQA1*01:01/DQB1*05:01 | 78-90 | YSVYGETLE | RYRYSVYGETLEK | 4.1 | HLA-DPA1*01:03/DPB1*04:01 | 74-85 | YRWYRYSVY | SEYRWYRYSVYG | 4.6 |
| HLA-DQA1*01:01/DQB1*05:01 | 79-91 | YSVYGETLE | YRYSVYGETLEKQ | 4.8 | HLA-DRB1*15:02 | 122-133 | FHNIGGRWT | KKRFHNIGGRWT | 4.1 | HLA-DQA1*01:01/DQB1*05:01 | 79-91 | YSVYGETLE | YRYSVYGETLEKQ | 4.8 |
| HLA-DRB1*15:02 | 77-91 | YSVYGETLE | RWYRYSVYGETLEKQ | 4.8 | HLA-DRB1*15:02 | 121-134 | FHNIGGRWT | EKKRFHNIGGRWTG | 4.1 | HLA-DRB1*15:02 | 77-91 | YSVYGETLE | RWYRYSVYGETLEKQ | 4.8 |
| HLA-DRB1*15:02 | 75-90 | WYRYSVYGE | EYRWYRYSVYGETLEK | 4.8 | HLA-DRB1*12:02 | 46-62 | IVYREGQPY | ACYDLCIVYREGQPYGV | 4.1 | HLA-DRB1*15:02 | 75-90 | WYRYSVYGE | EYRWYRYSVYGETLEK | 4.8 |
| HLA-DRB1*15:02 | 73-87 | WYRYSVYGE | ISEYRWYRYSVYGET | 4.9 | HLA-DRB1*12:02 | 71-83 | ISEYRRYRY | SKISEYRRYRYSV | 4.4 | HLA-DRB1*15:02 | 73-87 | WYRYSVYGE | ISEYRWYRYSVYGET | 4.9 |
| HLA-DRB1*12:02 | 45-62 | IVYREGQPY | FACYDLCIVYREGQPYGV | 4.9 | HLA-DRB1*15:02 | 78-92 | YSVYGETLE | RYRYSVYGETLEKQC | 4.4 | HLA-DRB1*12:02 | 45-62 | IVYREGQPY | FACYDLCIVYREGQPYGV | 4.9 |
| HLA-DQA1*01:01/DQB1*05:01 | 80-91 | YSVYGETLE | RYSVYGETLEKQ | 5 | HLA-DQA1*01:01/DQB1*05:01 | 78-91 | YSVYGETLE | RYRYSVYGETLEKQ | 4.6 | HLA-DQA1*01:01/DQB1*05:01 | 80-91 | YSVYGETLE | RYSVYGETLEKQ | 5 |
| HLA-DPA1*01:03/DPB1*04:01 | 71-87 | YRYSVYGET | SKISEYRWYRYSVYGET | 5 | HLA-DRB1*15:02 | 76-90 | YSVYGETLE | YRRYRYSVYGETLEK | 4.6 | HLA-DPA1*01:03/DPB1*04:01 | 71-87 | YRYSVYGET | SKISEYRWYRYSVYGET | 5 |
| HLA-DRB1*15:02 | 76-88 | WYRYSVYGE | YRWYRYSVYGETL | 5 | HLA-DQA1*01:01/DQB1*05:01 | 79-91 | YSVYGETLE | YRYSVYGETLEKQ | 4.8 | HLA-DRB1*15:02 | 76-88 | WYRYSVYGE | YRWYRYSVYGETL | 5 |
| HLA-DRB1*15:02 | 74-88 | WYRYSVYGE | SEYRWYRYSVYGETL | 5 | HLA-DRB1*15:02 | 68-80 | YSKISEYRR | KFYSKISEYRRYR | 4.8 | HLA-DRB1*15:02 | 74-88 | WYRYSVYGE | SEYRWYRYSVYGETL | 5 |
|  |  |  |  |  | HLA-DRB1*15:02 | 67-79 | YSKISEYRR | LKFYSKISEYRRY | 4.8 |  |  |  |  |  |
|  |  |  |  |  | HLA-DRB1*15:02 | 77-92 | YSVYGETLE | RRYRYSVYGETLEKQC | 4.9 |  |  |  |  |  |
|  |  |  |  |  | HLA-DRB1*12:02 | 45-62 | IVYREGQPY | FACYDLCIVYREGQPYGV | 4.9 |  |  |  |  |  |
|  |  |  |  |  | HLA-DQA1*01:01/DQB1*05:01 | 80-91 | YSVYGETLE | RYSVYGETLEKQ | 5 |  |  |  |  |  |
|  |  |  |  |  | HLA-DRB1*15:02 | 74-91 | YSVYGETLE | SEYRRYRYSVYGETLEKQ | 5 |  |  |  |  |  |

Table S5. The HLA-Ⅱ predicted epitopes of HPV-35 E7

| HPV35E7 REF | | | | | HPV35E7 H23Y | | | | | HPV35E7 L28F | | | | |
| --- | --- | --- | --- | --- | --- | --- | --- | --- | --- | --- | --- | --- | --- | --- |
| Allele | Location | Core_peptide | Peptide | Rank | Allele | Location | Core_peptide | Peptide | Rank | Allele | Location | Core_peptide | Peptide | Rank |
| HLA-DQA1*01:01/DQB1*05:01 | 2-13 | ITTLQDYVL | HGEITTLQDYVL | 0.01 | HLA-DQA1*01:01/DQB1*05:01 | 2-13 | ITTLQDYVL | HGEITTLQDYVL | 0.01 | HLA-DQA1*01:01/DQB1*05:01 | 2-13 | ITTLQDYVL | HGEITTLQDYVL | 0.01 |
| HLA-DQA1*01:01/DQB1*05:01 | 3-14 | ITTLQDYVL | GEITTLQDYVLD | 0.01 | HLA-DQA1*01:01/DQB1*05:01 | 3-14 | ITTLQDYVL | GEITTLQDYVLD | 0.01 | HLA-DQA1*01:01/DQB1*05:01 | 3-14 | ITTLQDYVL | GEITTLQDYVLD | 0.01 |
| HLA-DQA1*01:01/DQB1*05:01 | 2-15 | ITTLQDYVL | HGEITTLQDYVLDL | 0.01 | HLA-DQA1*01:01/DQB1*05:01 | 2-15 | ITTLQDYVL | HGEITTLQDYVLDL | 0.01 | HLA-DQA1*01:01/DQB1*05:01 | 2-15 | ITTLQDYVL | HGEITTLQDYVLDL | 0.01 |
| HLA-DQA1*01:01/DQB1*05:01 | 1-14 | ITTLQDYVL | MHGEITTLQDYVLD | 0.01 | HLA-DQA1*01:01/DQB1*05:01 | 1-14 | ITTLQDYVL | MHGEITTLQDYVLD | 0.01 | HLA-DQA1*01:01/DQB1*05:01 | 1-14 | ITTLQDYVL | MHGEITTLQDYVLD | 0.01 |
| HLA-DQA1*01:01/DQB1*05:01 | 2-16 | ITTLQDYVL | HGEITTLQDYVLDLE | 0.01 | HLA-DQA1*01:01/DQB1*05:01 | 2-16 | ITTLQDYVL | HGEITTLQDYVLDLE | 0.01 | HLA-DQA1*01:01/DQB1*05:01 | 2-16 | ITTLQDYVL | HGEITTLQDYVLDLE | 0.01 |
| HLA-DQA1*01:01/DQB1*05:01 | 2-14 | ITTLQDYVL | HGEITTLQDYVLD | 0.01 | HLA-DQA1*01:01/DQB1*05:01 | 2-14 | ITTLQDYVL | HGEITTLQDYVLD | 0.01 | HLA-DQA1*01:01/DQB1*05:01 | 2-14 | ITTLQDYVL | HGEITTLQDYVLD | 0.01 |
| HLA-DQA1*01:01/DQB1*05:01 | 1-16 | ITTLQDYVL | MHGEITTLQDYVLDLE | 0.03 | HLA-DQA1*01:01/DQB1*05:01 | 1-16 | ITTLQDYVL | MHGEITTLQDYVLDLE | 0.03 | HLA-DQA1*01:01/DQB1*05:01 | 1-16 | ITTLQDYVL | MHGEITTLQDYVLDLE | 0.03 |
| HLA-DQA1*01:01/DQB1*05:01 | 3-15 | ITTLQDYVL | GEITTLQDYVLDL | 0.04 | HLA-DQA1*01:01/DQB1*05:01 | 3-15 | ITTLQDYVL | GEITTLQDYVLDL | 0.04 | HLA-DQA1*01:01/DQB1*05:01 | 3-15 | ITTLQDYVL | GEITTLQDYVLDL | 0.04 |
| HLA-DQA1*01:01/DQB1*05:01 | 2-17 | ITTLQDYVL | HGEITTLQDYVLDLEP | 0.04 | HLA-DQA1*01:01/DQB1*05:01 | 2-17 | ITTLQDYVL | HGEITTLQDYVLDLEP | 0.04 | HLA-DQA1*01:01/DQB1*05:01 | 2-17 | ITTLQDYVL | HGEITTLQDYVLDLEP | 0.04 |
| HLA-DQA1*01:01/DQB1*05:01 | 2-19 | ITTLQDYVL | HGEITTLQDYVLDLEPEA | 0.05 | HLA-DQA1*01:01/DQB1*05:01 | 2-19 | ITTLQDYVL | HGEITTLQDYVLDLEPEA | 0.05 | HLA-DQA1*01:01/DQB1*05:01 | 2-19 | ITTLQDYVL | HGEITTLQDYVLDLEPEA | 0.05 |
| HLA-DQA1*01:01/DQB1*05:01 | 1-18 | ITTLQDYVL | MHGEITTLQDYVLDLEPE | 0.05 | HLA-DQA1*01:01/DQB1*05:01 | 1-18 | ITTLQDYVL | MHGEITTLQDYVLDLEPE | 0.05 | HLA-DQA1*01:01/DQB1*05:01 | 1-18 | ITTLQDYVL | MHGEITTLQDYVLDLEPE | 0.05 |
| HLA-DQA1*01:01/DQB1*05:01 | 1-17 | ITTLQDYVL | MHGEITTLQDYVLDLEP | 0.05 | HLA-DQA1*01:01/DQB1*05:01 | 1-17 | ITTLQDYVL | MHGEITTLQDYVLDLEP | 0.05 | HLA-DQA1*01:01/DQB1*05:01 | 1-17 | ITTLQDYVL | MHGEITTLQDYVLDLEP | 0.05 |
| HLA-DQA1*01:01/DQB1*05:01 | 1-15 | ITTLQDYVL | MHGEITTLQDYVLDL | 0.05 | HLA-DQA1*01:01/DQB1*05:01 | 1-15 | ITTLQDYVL | MHGEITTLQDYVLDL | 0.05 | HLA-DQA1*01:01/DQB1*05:01 | 1-15 | ITTLQDYVL | MHGEITTLQDYVLDL | 0.05 |
| HLA-DQA1*01:01/DQB1*05:01 | 3-16 | ITTLQDYVL | GEITTLQDYVLDLE | 0.05 | HLA-DQA1*01:01/DQB1*05:01 | 3-16 | ITTLQDYVL | GEITTLQDYVLDLE | 0.05 | HLA-DQA1*01:01/DQB1*05:01 | 3-16 | ITTLQDYVL | GEITTLQDYVLDLE | 0.05 |
| HLA-DQA1*01:01/DQB1*02:01 | 3-14 | ITTLQDYVL | GEITTLQDYVLD | 0.06 | HLA-DQA1*01:01/DQB1*02:01 | 3-14 | ITTLQDYVL | GEITTLQDYVLD | 0.06 | HLA-DQA1*01:01/DQB1*02:01 | 3-14 | ITTLQDYVL | GEITTLQDYVLD | 0.06 |
| HLA-DQA1*01:01/DQB1*02:01 | 2-14 | ITTLQDYVL | HGEITTLQDYVLD | 0.06 | HLA-DQA1*01:01/DQB1*02:01 | 2-14 | ITTLQDYVL | HGEITTLQDYVLD | 0.06 | HLA-DQA1*01:01/DQB1*02:01 | 2-14 | ITTLQDYVL | HGEITTLQDYVLD | 0.06 |
| HLA-DQA1*01:01/DQB1*05:01 | 2-18 | ITTLQDYVL | HGEITTLQDYVLDLEPE | 0.06 | HLA-DQA1*01:01/DQB1*05:01 | 2-18 | ITTLQDYVL | HGEITTLQDYVLDLEPE | 0.06 | HLA-DQA1*01:01/DQB1*05:01 | 2-18 | ITTLQDYVL | HGEITTLQDYVLDLEPE | 0.06 |
| HLA-DQA1*01:01/DQB1*05:01 | 3-17 | ITTLQDYVL | GEITTLQDYVLDLEP | 0.12 | HLA-DQA1*01:01/DQB1*05:01 | 3-17 | ITTLQDYVL | GEITTLQDYVLDLEP | 0.12 | HLA-DQA1*01:01/DQB1*05:01 | 3-17 | ITTLQDYVL | GEITTLQDYVLDLEP | 0.12 |
| HLA-DQA1*01:01/DQB1*05:01 | 3-19 | ITTLQDYVL | GEITTLQDYVLDLEPEA | 0.14 | HLA-DQA1*01:01/DQB1*05:01 | 3-19 | ITTLQDYVL | GEITTLQDYVLDLEPEA | 0.14 | HLA-DQA1*01:01/DQB1*05:01 | 3-19 | ITTLQDYVL | GEITTLQDYVLDLEPEA | 0.14 |
| HLA-DQA1*01:01/DQB1*05:01 | 3-20 | LQDYVLDLE | GEITTLQDYVLDLEPEAT | 0.15 | HLA-DQA1*01:01/DQB1*05:01 | 3-20 | LQDYVLDLE | GEITTLQDYVLDLEPEAT | 0.15 | HLA-DQA1*01:01/DQB1*05:01 | 3-20 | LQDYVLDLE | GEITTLQDYVLDLEPEAT | 0.15 |
| HLA-DQA1*01:01/DQB1*02:01 | 2-16 | ITTLQDYVL | HGEITTLQDYVLDLE | 0.15 | HLA-DQA1*01:01/DQB1*02:01 | 2-16 | ITTLQDYVL | HGEITTLQDYVLDLE | 0.15 | HLA-DQA1*01:01/DQB1*02:01 | 2-16 | ITTLQDYVL | HGEITTLQDYVLDLE | 0.15 |
| HLA-DQA1*01:01/DQB1*05:01 | 3-18 | ITTLQDYVL | GEITTLQDYVLDLEPE | 0.16 | HLA-DQA1*01:01/DQB1*05:01 | 3-18 | ITTLQDYVL | GEITTLQDYVLDLEPE | 0.16 | HLA-DQA1*01:01/DQB1*05:01 | 3-18 | ITTLQDYVL | GEITTLQDYVLDLEPE | 0.16 |
| HLA-DQA1*01:01/DQB1*02:01 | 1-14 | ITTLQDYVL | MHGEITTLQDYVLD | 0.17 | HLA-DQA1*01:01/DQB1*02:01 | 1-14 | ITTLQDYVL | MHGEITTLQDYVLD | 0.17 | HLA-DQA1*01:01/DQB1*02:01 | 1-14 | ITTLQDYVL | MHGEITTLQDYVLD | 0.17 |
| HLA-DQA1*01:01/DQB1*05:01 | 1-13 | ITTLQDYVL | MHGEITTLQDYVL | 0.18 | HLA-DQA1*01:01/DQB1*05:01 | 1-13 | ITTLQDYVL | MHGEITTLQDYVL | 0.18 | HLA-DQA1*01:01/DQB1*05:01 | 1-13 | ITTLQDYVL | MHGEITTLQDYVL | 0.18 |
| HLA-DQA1*01:01/DQB1*05:01 | 4-16 | ITTLQDYVL | EITTLQDYVLDLE | 0.21 | HLA-DQA1*01:01/DQB1*05:01 | 4-16 | ITTLQDYVL | EITTLQDYVLDLE | 0.21 | HLA-DQA1*01:01/DQB1*05:01 | 4-16 | ITTLQDYVL | EITTLQDYVLDLE | 0.21 |
| HLA-DQA1*01:01/DQB1*05:01 | 4-15 | ITTLQDYVL | EITTLQDYVLDL | 0.22 | HLA-DQA1*01:01/DQB1*05:01 | 4-15 | ITTLQDYVL | EITTLQDYVLDL | 0.22 | HLA-DQA1*01:01/DQB1*05:01 | 4-15 | ITTLQDYVL | EITTLQDYVLDL | 0.22 |
| HLA-DQA1*01:01/DQB1*05:01 | 6-17 | LQDYVLDLE | TTLQDYVLDLEP | 0.23 | HLA-DQA1*01:01/DQB1*05:01 | 6-17 | LQDYVLDLE | TTLQDYVLDLEP | 0.23 | HLA-DQA1*01:01/DQB1*05:01 | 6-17 | LQDYVLDLE | TTLQDYVLDLEP | 0.23 |
| HLA-DQA1*01:01/DQB1*02:01 | 2-15 | ITTLQDYVL | HGEITTLQDYVLDL | 0.26 | HLA-DQA1*01:01/DQB1*02:01 | 2-15 | ITTLQDYVL | HGEITTLQDYVLDL | 0.26 | HLA-DQA1*01:01/DQB1*02:01 | 2-15 | ITTLQDYVL | HGEITTLQDYVLDL | 0.26 |
| HLA-DQA1*01:01/DQB1*05:01 | 4-19 | LQDYVLDLE | EITTLQDYVLDLEPEA | 0.3 | HLA-DQA1*01:01/DQB1*05:01 | 4-19 | LQDYVLDLE | EITTLQDYVLDLEPEA | 0.3 | HLA-DQA1*01:01/DQB1*05:01 | 4-19 | LQDYVLDLE | EITTLQDYVLDLEPEA | 0.3 |
| HLA-DQA1*01:01/DQB1*05:01 | 4-17 | ITTLQDYVL | EITTLQDYVLDLEP | 0.31 | HLA-DQA1*01:01/DQB1*05:01 | 4-17 | ITTLQDYVL | EITTLQDYVLDLEP | 0.31 | HLA-DQA1*01:01/DQB1*05:01 | 4-17 | ITTLQDYVL | EITTLQDYVLDLEP | 0.31 |
| HLA-DQA1*01:01/DQB1*02:01 | 1-16 | ITTLQDYVL | MHGEITTLQDYVLDLE | 0.33 | HLA-DQA1*01:01/DQB1*02:01 | 1-16 | ITTLQDYVL | MHGEITTLQDYVLDLE | 0.33 | HLA-DQA1*01:01/DQB1*02:01 | 1-16 | ITTLQDYVL | MHGEITTLQDYVLDLE | 0.33 |
| HLA-DQA1*01:01/DQB1*05:01 | 4-18 | LQDYVLDLE | EITTLQDYVLDLEPE | 0.33 | HLA-DQA1*01:01/DQB1*05:01 | 4-18 | LQDYVLDLE | EITTLQDYVLDLEPE | 0.33 | HLA-DQA1*01:01/DQB1*05:01 | 4-18 | LQDYVLDLE | EITTLQDYVLDLEPE | 0.33 |
| HLA-DQA1*01:01/DQB1*02:01 | 2-13 | ITTLQDYVL | HGEITTLQDYVL | 0.34 | HLA-DQA1*01:01/DQB1*02:01 | 2-13 | ITTLQDYVL | HGEITTLQDYVL | 0.34 | HLA-DQA1*01:01/DQB1*02:01 | 2-13 | ITTLQDYVL | HGEITTLQDYVL | 0.34 |
| HLA-DQA1*01:01/DQB1*05:01 | 74-86 | IRKLEDLLM | HIDIRKLEDLLMG | 0.36 | HLA-DQA1*01:01/DQB1*05:01 | 74-86 | IRKLEDLLM | HIDIRKLEDLLMG | 0.36 | HLA-DQA1*01:01/DQB1*05:01 | 74-86 | IRKLEDLLM | HIDIRKLEDLLMG | 0.36 |
| HLA-DQA1*01:01/DQB1*02:01 | 1-17 | ITTLQDYVL | MHGEITTLQDYVLDLEP | 0.43 | HLA-DQA1*01:01/DQB1*02:01 | 1-17 | ITTLQDYVL | MHGEITTLQDYVLDLEP | 0.43 | HLA-DQA1*01:01/DQB1*02:01 | 1-17 | ITTLQDYVL | MHGEITTLQDYVLDLEP | 0.43 |
| HLA-DQA1*01:01/DQB1*05:01 | 4-20 | LQDYVLDLE | EITTLQDYVLDLEPEAT | 0.44 | HLA-DQA1*01:01/DQB1*05:01 | 4-20 | LQDYVLDLE | EITTLQDYVLDLEPEAT | 0.44 | HLA-DQA1*01:01/DQB1*05:01 | 4-20 | LQDYVLDLE | EITTLQDYVLDLEPEAT | 0.44 |
| HLA-DQA1*01:01/DQB1*05:01 | 5-19 | LQDYVLDLE | ITTLQDYVLDLEPEA | 0.45 | HLA-DQA1*01:01/DQB1*05:01 | 5-19 | LQDYVLDLE | ITTLQDYVLDLEPEA | 0.45 | HLA-DQA1*01:01/DQB1*05:01 | 5-19 | LQDYVLDLE | ITTLQDYVLDLEPEA | 0.45 |
| HLA-DQA1*01:01/DQB1*05:01 | 6-18 | LQDYVLDLE | TTLQDYVLDLEPE | 0.46 | HLA-DQA1*01:01/DQB1*05:01 | 6-18 | LQDYVLDLE | TTLQDYVLDLEPE | 0.46 | HLA-DQA1*01:01/DQB1*05:01 | 6-18 | LQDYVLDLE | TTLQDYVLDLEPE | 0.46 |
| HLA-DQA1*01:01/DQB1*02:01 | 1-15 | ITTLQDYVL | MHGEITTLQDYVLDL | 0.49 | HLA-DQA1*01:01/DQB1*02:01 | 1-15 | ITTLQDYVL | MHGEITTLQDYVLDL | 0.49 | HLA-DQA1*01:01/DQB1*02:01 | 1-15 | ITTLQDYVL | MHGEITTLQDYVLDL | 0.49 |
| HLA-DQA1*01:01/DQB1*02:01 | 2-17 | ITTLQDYVL | HGEITTLQDYVLDLEP | 0.52 | HLA-DQA1*01:01/DQB1*02:01 | 2-17 | ITTLQDYVL | HGEITTLQDYVLDLEP | 0.52 | HLA-DQA1*01:01/DQB1*02:01 | 2-17 | ITTLQDYVL | HGEITTLQDYVLDLEP | 0.52 |
| HLA-DQA1*01:01/DQB1*05:01 | 74-87 | IRKLEDLLM | HIDIRKLEDLLMGT | 0.53 | HLA-DQA1*01:01/DQB1*05:01 | 74-87 | IRKLEDLLM | HIDIRKLEDLLMGT | 0.53 | HLA-DQA1*01:01/DQB1*05:01 | 74-87 | IRKLEDLLM | HIDIRKLEDLLMGT | 0.53 |
| HLA-DQA1*01:01/DQB1*05:01 | 5-18 | LQDYVLDLE | ITTLQDYVLDLEPE | 0.53 | HLA-DQA1*01:01/DQB1*05:01 | 5-18 | LQDYVLDLE | ITTLQDYVLDLEPE | 0.53 | HLA-DQA1*01:01/DQB1*05:01 | 5-18 | LQDYVLDLE | ITTLQDYVLDLEPE | 0.53 |
| HLA-DQA1*01:01/DQB1*02:01 | 3-15 | ITTLQDYVL | GEITTLQDYVLDL | 0.55 | HLA-DQA1*01:01/DQB1*02:01 | 3-15 | ITTLQDYVL | GEITTLQDYVLDL | 0.55 | HLA-DQA1*01:01/DQB1*02:01 | 3-15 | ITTLQDYVL | GEITTLQDYVLDL | 0.55 |
| HLA-DQA1*01:01/DQB1*02:01 | 1-18 | ITTLQDYVL | MHGEITTLQDYVLDLEPE | 0.57 | HLA-DQA1*01:01/DQB1*02:01 | 1-18 | ITTLQDYVL | MHGEITTLQDYVLDLEPE | 0.57 | HLA-DQA1*01:01/DQB1*02:01 | 1-18 | ITTLQDYVL | MHGEITTLQDYVLDLEPE | 0.57 |
| HLA-DQA1*01:01/DQB1*05:01 | 7-18 | LQDYVLDLE | TLQDYVLDLEPE | 0.59 | HLA-DQA1*01:01/DQB1*05:01 | 7-18 | LQDYVLDLE | TLQDYVLDLEPE | 0.59 | HLA-DQA1*01:01/DQB1*05:01 | 7-18 | LQDYVLDLE | TLQDYVLDLEPE | 0.59 |
| HLA-DQA1*01:01/DQB1*05:01 | 5-17 | LQDYVLDLE | ITTLQDYVLDLEP | 0.61 | HLA-DQA1*01:01/DQB1*05:01 | 5-17 | LQDYVLDLE | ITTLQDYVLDLEP | 0.61 | HLA-DQA1*01:01/DQB1*05:01 | 5-17 | LQDYVLDLE | ITTLQDYVLDLEP | 0.61 |
| HLA-DQA1*01:01/DQB1*05:01 | 6-19 | LQDYVLDLE | TTLQDYVLDLEPEA | 0.61 | HLA-DQA1*01:01/DQB1*05:01 | 6-19 | LQDYVLDLE | TTLQDYVLDLEPEA | 0.61 | HLA-DQA1*01:01/DQB1*05:01 | 6-19 | LQDYVLDLE | TTLQDYVLDLEPEA | 0.61 |
| HLA-DQA1*01:01/DQB1*05:01 | 4-21 | LQDYVLDLE | EITTLQDYVLDLEPEATD | 0.63 | HLA-DQA1*01:01/DQB1*05:01 | 4-21 | LQDYVLDLE | EITTLQDYVLDLEPEATD | 0.63 | HLA-DQA1*01:01/DQB1*05:01 | 4-21 | LQDYVLDLE | EITTLQDYVLDLEPEATD | 0.63 |
| HLA-DQA1*01:01/DQB1*02:01 | 3-16 | ITTLQDYVL | GEITTLQDYVLDLE | 0.63 | HLA-DQA1*01:01/DQB1*02:01 | 3-16 | ITTLQDYVL | GEITTLQDYVLDLE | 0.63 | HLA-DQA1*01:01/DQB1*02:01 | 3-16 | ITTLQDYVL | GEITTLQDYVLDLE | 0.63 |

Continued 1 Table S5. The HLA-Ⅱ predicted epitopes of HPV-35 E7

| HPV35E7 REF | | | | | HPV35E7 H23Y | | | | | HPV35E7 L28F | | | | |
| --- | --- | --- | --- | --- | --- | --- | --- | --- | --- | --- | --- | --- | --- | --- |
| Allele | Location | Core_peptide | Peptide | Rank | Allele | Location | Core_peptide | Peptide | Rank | Allele | Location | Core_peptide | Peptide | Rank |
| HLA-DQA1*01:01/DQB1*02:01 | 2-18 | ITTLQDYVL | HGEITTLQDYVLDLEPE | 0.66 | HLA-DQA1*01:01/DQB1*02:01 | 2-18 | ITTLQDYVL | HGEITTLQDYVLDLEPE | 0.66 | HLA-DQA1*01:01/DQB1*02:01 | 2-18 | ITTLQDYVL | HGEITTLQDYVLDLEPE | 0.66 |
| HLA-DQA1*01:01/DQB1*05:01 | 75-86 | IRKLEDLLM | IDIRKLEDLLMG | 0.69 | HLA-DQA1*01:01/DQB1*05:01 | 75-86 | IRKLEDLLM | IDIRKLEDLLMG | 0.69 | HLA-DQA1*01:01/DQB1*05:01 | 75-86 | IRKLEDLLM | IDIRKLEDLLMG | 0.69 |
| HLA-DRB1*12:02 | 74-86 | IRKLEDLLM | HIDIRKLEDLLMG | 0.69 | HLA-DRB1*12:02 | 74-86 | IRKLEDLLM | HIDIRKLEDLLMG | 0.69 | HLA-DRB1*12:02 | 74-86 | IRKLEDLLM | HIDIRKLEDLLMG | 0.69 |
| HLA-DRB1*12:02 | 75-86 | IRKLEDLLM | IDIRKLEDLLMG | 0.7 | HLA-DRB1*12:02 | 75-86 | IRKLEDLLM | IDIRKLEDLLMG | 0.7 | HLA-DRB1*12:02 | 75-86 | IRKLEDLLM | IDIRKLEDLLMG | 0.7 |
| HLA-DQA1*01:01/DQB1*05:01 | 5-20 | LQDYVLDLE | ITTLQDYVLDLEPEAT | 0.74 | HLA-DQA1*01:01/DQB1*05:01 | 5-20 | LQDYVLDLE | ITTLQDYVLDLEPEAT | 0.74 | HLA-DQA1*01:01/DQB1*05:01 | 5-20 | LQDYVLDLE | ITTLQDYVLDLEPEAT | 0.74 |
| HLA-DQA1*01:01/DQB1*05:01 | 73-87 | IRKLEDLLM | THIDIRKLEDLLMGT | 0.79 | HLA-DQA1*01:01/DQB1*05:01 | 73-87 | IRKLEDLLM | THIDIRKLEDLLMGT | 0.79 | HLA-DQA1*01:01/DQB1*05:01 | 73-87 | IRKLEDLLM | THIDIRKLEDLLMGT | 0.79 |
| HLA-DRB1*12:02 | 74-87 | IRKLEDLLM | HIDIRKLEDLLMGT | 0.8 | HLA-DRB1*12:02 | 74-87 | IRKLEDLLM | HIDIRKLEDLLMGT | 0.8 | HLA-DRB1*12:02 | 74-87 | IRKLEDLLM | HIDIRKLEDLLMGT | 0.8 |
| HLA-DQA1*01:01/DQB1*05:01 | 73-86 | IRKLEDLLM | THIDIRKLEDLLMG | 0.87 | HLA-DQA1*01:01/DQB1*05:01 | 73-86 | IRKLEDLLM | THIDIRKLEDLLMG | 0.87 | HLA-DQA1*01:01/DQB1*05:01 | 73-86 | IRKLEDLLM | THIDIRKLEDLLMG | 0.87 |
| HLA-DQA1*01:01/DQB1*05:01 | 74-85 | IRKLEDLLM | HIDIRKLEDLLM | 0.88 | HLA-DQA1*01:01/DQB1*05:01 | 74-85 | IRKLEDLLM | HIDIRKLEDLLM | 0.88 | HLA-DQA1*01:01/DQB1*05:01 | 74-85 | IRKLEDLLM | HIDIRKLEDLLM | 0.88 |
| HLA-DQA1*01:01/DQB1*05:01 | 5-21 | LQDYVLDLE | ITTLQDYVLDLEPEATD | 0.9 | HLA-DQA1*01:01/DQB1*05:01 | 5-21 | LQDYVLDLE | ITTLQDYVLDLEPEATD | 0.9 | HLA-DQA1*01:01/DQB1*05:01 | 5-21 | LQDYVLDLE | ITTLQDYVLDLEPEATD | 0.9 |
| HLA-DQA1*01:01/DQB1*05:01 | 5-16 | LQDYVLDLE | ITTLQDYVLDLE | 0.96 | HLA-DQA1*01:01/DQB1*05:01 | 5-16 | LQDYVLDLE | ITTLQDYVLDLE | 0.96 | HLA-DQA1*01:01/DQB1*05:01 | 5-16 | LQDYVLDLE | ITTLQDYVLDLE | 0.96 |
| HLA-DQA1*01:01/DQB1*05:01 | 75-87 | IRKLEDLLM | IDIRKLEDLLMGT | 0.97 | HLA-DQA1*01:01/DQB1*05:01 | 75-87 | IRKLEDLLM | IDIRKLEDLLMGT | 0.97 | HLA-DQA1*01:01/DQB1*05:01 | 75-87 | IRKLEDLLM | IDIRKLEDLLMGT | 0.97 |
| HLA-DQA1*01:01/DQB1*05:01 | 74-88 | IRKLEDLLM | HIDIRKLEDLLMGTF | 0.98 | HLA-DQA1*01:01/DQB1*05:01 | 74-88 | IRKLEDLLM | HIDIRKLEDLLMGTF | 0.98 | HLA-DQA1*01:01/DQB1*05:01 | 74-88 | IRKLEDLLM | HIDIRKLEDLLMGTF | 0.98 |
| HLA-DQA1*01:01/DQB1*02:01 | 2-19 | ITTLQDYVL | HGEITTLQDYVLDLEPEA | 1 | HLA-DQA1*01:01/DQB1*02:01 | 2-19 | ITTLQDYVL | HGEITTLQDYVLDLEPEA | 1 | HLA-DQA1*01:01/DQB1*02:01 | 2-19 | ITTLQDYVL | HGEITTLQDYVLDLEPEA | 1 |
| HLA-DQA1*01:01/DQB1*05:01 | 6-20 | LQDYVLDLE | TTLQDYVLDLEPEAT | 1.1 | HLA-DQA1*01:01/DQB1*05:01 | 6-20 | LQDYVLDLE | TTLQDYVLDLEPEAT | 1.1 | HLA-DQA1*01:01/DQB1*05:01 | 6-20 | LQDYVLDLE | TTLQDYVLDLEPEAT | 1.1 |
| HLA-DRB1*12:02 | 75-87 | IRKLEDLLM | IDIRKLEDLLMGT | 1.1 | HLA-DRB1*12:02 | 75-87 | IRKLEDLLM | IDIRKLEDLLMGT | 1.1 | HLA-DRB1*12:02 | 75-87 | IRKLEDLLM | IDIRKLEDLLMGT | 1.1 |
| HLA-DRB1*12:02 | 73-87 | IRKLEDLLM | THIDIRKLEDLLMGT | 1.1 | HLA-DRB1*12:02 | 73-87 | IRKLEDLLM | THIDIRKLEDLLMGT | 1.1 | HLA-DRB1*12:02 | 73-87 | IRKLEDLLM | THIDIRKLEDLLMGT | 1.1 |
| HLA-DQA1*01:01/DQB1*05:01 | 5-22 | LQDYVLDLE | ITTLQDYVLDLEPEATDL | 1.2 | HLA-DQA1*01:01/DQB1*05:01 | 5-22 | LQDYVLDLE | ITTLQDYVLDLEPEATDL | 1.2 | HLA-DQA1*01:01/DQB1*05:01 | 5-22 | LQDYVLDLE | ITTLQDYVLDLEPEATDL | 1.2 |
| HLA-DQA1*01:01/DQB1*05:01 | 73-88 | IRKLEDLLM | THIDIRKLEDLLMGTF | 1.2 | HLA-DQA1*01:01/DQB1*05:01 | 73-88 | IRKLEDLLM | THIDIRKLEDLLMGTF | 1.2 | HLA-DQA1*01:01/DQB1*05:01 | 73-88 | IRKLEDLLM | THIDIRKLEDLLMGTF | 1.2 |
| HLA-DQA1*01:01/DQB1*05:01 | 7-19 | LQDYVLDLE | TLQDYVLDLEPEA | 1.3 | HLA-DQA1*01:01/DQB1*05:01 | 7-19 | LQDYVLDLE | TLQDYVLDLEPEA | 1.3 | HLA-DQA1*01:01/DQB1*05:01 | 7-19 | LQDYVLDLE | TLQDYVLDLEPEA | 1.3 |
| HLA-DQA1*01:01/DQB1*05:01 | 72-87 | IRKLEDLLM | STHIDIRKLEDLLMGT | 1.3 | HLA-DQA1*01:01/DQB1*05:01 | 72-87 | IRKLEDLLM | STHIDIRKLEDLLMGT | 1.3 | HLA-DQA1*01:01/DQB1*05:01 | 72-87 | IRKLEDLLM | STHIDIRKLEDLLMGT | 1.3 |
| HLA-DRB1*12:02 | 73-86 | IRKLEDLLM | THIDIRKLEDLLMG | 1.3 | HLA-DRB1*12:02 | 73-86 | IRKLEDLLM | THIDIRKLEDLLMG | 1.3 | HLA-DRB1*12:02 | 73-86 | IRKLEDLLM | THIDIRKLEDLLMG | 1.3 |
| HLA-DRB1*12:02 | 74-88 | IRKLEDLLM | HIDIRKLEDLLMGTF | 1.4 | HLA-DRB1*12:02 | 74-88 | IRKLEDLLM | HIDIRKLEDLLMGTF | 1.4 | HLA-DRB1*12:02 | 74-88 | IRKLEDLLM | HIDIRKLEDLLMGTF | 1.4 |
| HLA-DQA1*01:01/DQB1*02:01 | 1-13 | ITTLQDYVL | MHGEITTLQDYVL | 1.5 | HLA-DQA1*01:01/DQB1*02:01 | 1-13 | ITTLQDYVL | MHGEITTLQDYVL | 1.5 | HLA-DQA1*01:01/DQB1*02:01 | 1-13 | ITTLQDYVL | MHGEITTLQDYVL | 1.5 |
| HLA-DQA1*01:01/DQB1*05:01 | 6-21 | LQDYVLDLE | TTLQDYVLDLEPEATD | 1.5 | HLA-DQA1*01:01/DQB1*05:01 | 6-21 | LQDYVLDLE | TTLQDYVLDLEPEATD | 1.5 | HLA-DQA1*01:01/DQB1*05:01 | 6-21 | LQDYVLDLE | TTLQDYVLDLEPEATD | 1.5 |
| HLA-DRB1*12:02 | 74-85 | IRKLEDLLM | HIDIRKLEDLLM | 1.5 | HLA-DRB1*12:02 | 74-85 | IRKLEDLLM | HIDIRKLEDLLM | 1.5 | HLA-DRB1*12:02 | 74-85 | IRKLEDLLM | HIDIRKLEDLLM | 1.5 |
| HLA-DRB1*12:02 | 72-87 | IRKLEDLLM | STHIDIRKLEDLLMGT | 1.5 | HLA-DRB1*12:02 | 72-87 | IRKLEDLLM | STHIDIRKLEDLLMGT | 1.5 | HLA-DRB1*12:02 | 72-87 | IRKLEDLLM | STHIDIRKLEDLLMGT | 1.5 |
| HLA-DRB1*12:02 | 73-88 | IRKLEDLLM | THIDIRKLEDLLMGTF | 1.5 | HLA-DRB1*12:02 | 73-88 | IRKLEDLLM | THIDIRKLEDLLMGTF | 1.5 | HLA-DRB1*12:02 | 73-88 | IRKLEDLLM | THIDIRKLEDLLMGTF | 1.5 |
| HLA-DQA1*01:01/DQB1*05:01 | 72-88 | IRKLEDLLM | STHIDIRKLEDLLMGTF | 1.6 | HLA-DQA1*01:01/DQB1*05:01 | 72-88 | IRKLEDLLM | STHIDIRKLEDLLMGTF | 1.6 | HLA-DQA1*01:01/DQB1*05:01 | 72-88 | IRKLEDLLM | STHIDIRKLEDLLMGTF | 1.6 |
| HLA-DQA1*01:01/DQB1*02:01 | 9-21 | YVLDLEPEA | QDYVLDLEPEATD | 1.7 | HLA-DQA1*01:01/DQB1*02:01 | 9-21 | YVLDLEPEA | QDYVLDLEPEATD | 1.7 | HLA-DQA1*01:01/DQB1*02:01 | 9-21 | YVLDLEPEA | QDYVLDLEPEATD | 1.7 |
| HLA-DQA1*01:01/DQB1*02:01 | 3-17 | ITTLQDYVL | GEITTLQDYVLDLEP | 1.7 | HLA-DQA1*01:01/DQB1*02:01 | 3-17 | ITTLQDYVL | GEITTLQDYVLDLEP | 1.7 | HLA-DQA1*01:01/DQB1*02:01 | 3-17 | ITTLQDYVL | GEITTLQDYVLDLEP | 1.7 |
| HLA-DQA1*01:01/DQB1*02:01 | 74-87 | IRKLEDLLM | HIDIRKLEDLLMGT | 1.8 | HLA-DQA1*01:01/DQB1*02:01 | 74-87 | IRKLEDLLM | HIDIRKLEDLLMGT | 1.8 | HLA-DQA1*01:01/DQB1*02:01 | 74-87 | IRKLEDLLM | HIDIRKLEDLLMGT | 1.8 |
| HLA-DRB1*12:02 | 76-87 | IRKLEDLLM | DIRKLEDLLMGT | 1.8 | HLA-DRB1*12:02 | 76-87 | IRKLEDLLM | DIRKLEDLLMGT | 1.8 | HLA-DRB1*12:02 | 76-87 | IRKLEDLLM | DIRKLEDLLMGT | 1.8 |
| HLA-DQA1*01:01/DQB1*02:01 | 75-86 | IRKLEDLLM | IDIRKLEDLLMG | 1.9 | HLA-DQA1*01:01/DQB1*02:01 | 75-86 | IRKLEDLLM | IDIRKLEDLLMG | 1.9 | HLA-DQA1*01:01/DQB1*02:01 | 75-86 | IRKLEDLLM | IDIRKLEDLLMG | 1.9 |
| HLA-DQA1*01:01/DQB1*05:01 | 72-86 | IRKLEDLLM | STHIDIRKLEDLLMG | 1.9 | HLA-DQA1*01:01/DQB1*05:01 | 72-86 | IRKLEDLLM | STHIDIRKLEDLLMG | 1.9 | HLA-DQA1*01:01/DQB1*05:01 | 72-86 | IRKLEDLLM | STHIDIRKLEDLLMG | 1.9 |
| HLA-DRB1*12:02 | 2-14 | ITTLQDYVL | HGEITTLQDYVLD | 1.9 | HLA-DRB1*12:02 | 2-14 | ITTLQDYVL | HGEITTLQDYVLD | 1.9 | HLA-DRB1*12:02 | 2-14 | ITTLQDYVL | HGEITTLQDYVLD | 1.9 |
| HLA-DRB1*12:02 | 72-88 | IRKLEDLLM | STHIDIRKLEDLLMGTF | 1.9 | HLA-DRB1*12:02 | 72-88 | IRKLEDLLM | STHIDIRKLEDLLMGTF | 1.9 | HLA-DRB1*12:02 | 72-88 | IRKLEDLLM | STHIDIRKLEDLLMGTF | 1.9 |
| HLA-DQA1*01:01/DQB1*05:01 | 71-88 | IRKLEDLLM | QSTHIDIRKLEDLLMGTF | 2 | HLA-DQA1*01:01/DQB1*05:01 | 71-88 | IRKLEDLLM | QSTHIDIRKLEDLLMGTF | 2 | HLA-DQA1*01:01/DQB1*05:01 | 71-88 | IRKLEDLLM | QSTHIDIRKLEDLLMGTF | 2 |
| HLA-DQA1*01:01/DQB1*02:01 | 74-86 | IRKLEDLLM | HIDIRKLEDLLMG | 2 | HLA-DQA1*01:01/DQB1*02:01 | 74-86 | IRKLEDLLM | HIDIRKLEDLLMG | 2 | HLA-DQA1*01:01/DQB1*02:01 | 74-86 | IRKLEDLLM | HIDIRKLEDLLMG | 2 |
| HLA-DRB1*12:02 | 3-14 | ITTLQDYVL | GEITTLQDYVLD | 2 | HLA-DRB1*12:02 | 3-14 | ITTLQDYVL | GEITTLQDYVLD | 2 | HLA-DRB1*12:02 | 3-14 | ITTLQDYVL | GEITTLQDYVLD | 2 |
| HLA-DQA1*01:01/DQB1*05:01 | 71-87 | IRKLEDLLM | QSTHIDIRKLEDLLMGT | 2.1 | HLA-DQA1*01:01/DQB1*05:01 | 71-87 | IRKLEDLLM | QSTHIDIRKLEDLLMGT | 2.1 | HLA-DQA1*01:01/DQB1*05:01 | 71-87 | IRKLEDLLM | QSTHIDIRKLEDLLMGT | 2.1 |
| HLA-DQA1*01:01/DQB1*05:01 | 73-85 | IRKLEDLLM | THIDIRKLEDLLM | 2.2 | HLA-DQA1*01:01/DQB1*05:01 | 73-85 | IRKLEDLLM | THIDIRKLEDLLM | 2.2 | HLA-DQA1*01:01/DQB1*05:01 | 73-85 | IRKLEDLLM | THIDIRKLEDLLM | 2.2 |
| HLA-DQA1*01:01/DQB1*05:01 | 74-89 | IRKLEDLLM | HIDIRKLEDLLMGTFG | 2.2 | HLA-DQA1*01:01/DQB1*05:01 | 74-89 | IRKLEDLLM | HIDIRKLEDLLMGTFG | 2.2 | HLA-DQA1*01:01/DQB1*05:01 | 74-89 | IRKLEDLLM | HIDIRKLEDLLMGTFG | 2.2 |
| HLA-DRB1*12:02 | 71-87 | IRKLEDLLM | QSTHIDIRKLEDLLMGT | 2.2 | HLA-DRB1*12:02 | 71-87 | IRKLEDLLM | QSTHIDIRKLEDLLMGT | 2.2 | HLA-DRB1*12:02 | 71-87 | IRKLEDLLM | QSTHIDIRKLEDLLMGT | 2.2 |
| HLA-DQA1*01:01/DQB1*05:01 | 73-89 | IRKLEDLLM | THIDIRKLEDLLMGTFG | 2.3 | HLA-DQA1*01:01/DQB1*05:01 | 73-89 | IRKLEDLLM | THIDIRKLEDLLMGTFG | 2.3 | HLA-DQA1*01:01/DQB1*05:01 | 73-89 | IRKLEDLLM | THIDIRKLEDLLMGTFG | 2.3 |
| HLA-DQA1*01:01/DQB1*05:01 | 6-22 | LQDYVLDLE | TTLQDYVLDLEPEATDL | 2.3 | HLA-DQA1*01:01/DQB1*05:01 | 6-22 | LQDYVLDLE | TTLQDYVLDLEPEATDL | 2.3 | HLA-DQA1*01:01/DQB1*05:01 | 6-22 | LQDYVLDLE | TTLQDYVLDLEPEATDL | 2.3 |
| HLA-DRB1*12:02 | 75-88 | IRKLEDLLM | IDIRKLEDLLMGTF | 2.3 | HLA-DRB1*12:02 | 75-88 | IRKLEDLLM | IDIRKLEDLLMGTF | 2.3 | HLA-DRB1*12:02 | 75-88 | IRKLEDLLM | IDIRKLEDLLMGTF | 2.3 |
| HLA-DRB1*12:02 | 71-88 | IRKLEDLLM | QSTHIDIRKLEDLLMGTF | 2.3 | HLA-DRB1*12:02 | 71-88 | IRKLEDLLM | QSTHIDIRKLEDLLMGTF | 2.3 | HLA-DRB1*12:02 | 71-88 | IRKLEDLLM | QSTHIDIRKLEDLLMGTF | 2.3 |
| HLA-DRB1*12:02 | 74-89 | IRKLEDLLM | HIDIRKLEDLLMGTFG | 2.3 | HLA-DRB1*12:02 | 74-89 | IRKLEDLLM | HIDIRKLEDLLMGTFG | 2.3 | HLA-DRB1*12:02 | 74-89 | IRKLEDLLM | HIDIRKLEDLLMGTFG | 2.3 |
| HLA-DQA1*01:01/DQB1*05:01 | 72-89 | IRKLEDLLM | STHIDIRKLEDLLMGTFG | 2.4 | HLA-DQA1*01:01/DQB1*05:01 | 72-89 | IRKLEDLLM | STHIDIRKLEDLLMGTFG | 2.4 | HLA-DQA1*01:01/DQB1*05:01 | 72-89 | IRKLEDLLM | STHIDIRKLEDLLMGTFG | 2.4 |
| HLA-DRB1*12:02 | 72-86 | IRKLEDLLM | STHIDIRKLEDLLMG | 2.4 | HLA-DRB1*12:02 | 72-86 | IRKLEDLLM | STHIDIRKLEDLLMG | 2.4 | HLA-DRB1*12:02 | 72-86 | IRKLEDLLM | STHIDIRKLEDLLMG | 2.4 |
| HLA-DRB1*12:02 | 73-89 | IRKLEDLLM | THIDIRKLEDLLMGTFG | 2.4 | HLA-DRB1*12:02 | 73-89 | IRKLEDLLM | THIDIRKLEDLLMGTFG | 2.4 | HLA-DRB1*12:02 | 73-89 | IRKLEDLLM | THIDIRKLEDLLMGTFG | 2.4 |
| HLA-DRB1*12:02 | 72-89 | IRKLEDLLM | STHIDIRKLEDLLMGTFG | 2.5 | HLA-DRB1*12:02 | 72-89 | IRKLEDLLM | STHIDIRKLEDLLMGTFG | 2.5 | HLA-DRB1*12:02 | 72-89 | IRKLEDLLM | STHIDIRKLEDLLMGTFG | 2.5 |
| HLA-DQA1*01:01/DQB1*02:01 | 9-20 | YVLDLEPEA | QDYVLDLEPEAT | 2.6 | HLA-DQA1*01:01/DQB1*02:01 | 9-20 | YVLDLEPEA | QDYVLDLEPEAT | 2.6 | HLA-DQA1*01:01/DQB1*02:01 | 9-20 | YVLDLEPEA | QDYVLDLEPEAT | 2.6 |
| HLA-DQA1*01:01/DQB1*02:01 | 8-21 | YVLDLEPEA | LQDYVLDLEPEATD | 2.6 | HLA-DQA1*01:01/DQB1*02:01 | 8-21 | YVLDLEPEA | LQDYVLDLEPEATD | 2.6 | HLA-DQA1*01:01/DQB1*02:01 | 8-21 | YVLDLEPEA | LQDYVLDLEPEATD | 2.6 |
| HLA-DQA1*01:01/DQB1*02:01 | 7-21 | YVLDLEPEA | TLQDYVLDLEPEATD | 2.6 | HLA-DQA1*01:01/DQB1*02:01 | 7-21 | YVLDLEPEA | TLQDYVLDLEPEATD | 2.6 | HLA-DQA1*01:01/DQB1*02:01 | 7-21 | YVLDLEPEA | TLQDYVLDLEPEATD | 2.6 |
| HLA-DQA1*01:01/DQB1*05:01 | 75-88 | IRKLEDLLM | IDIRKLEDLLMGTF | 2.7 | HLA-DQA1*01:01/DQB1*05:01 | 75-88 | IRKLEDLLM | IDIRKLEDLLMGTF | 2.7 | HLA-DQA1*01:01/DQB1*05:01 | 75-88 | IRKLEDLLM | IDIRKLEDLLMGTF | 2.7 |
| HLA-DRB1*12:02 | 70-87 | IRKLEDLLM | VQSTHIDIRKLEDLLMGT | 2.8 | HLA-DRB1*12:02 | 70-87 | IRKLEDLLM | VQSTHIDIRKLEDLLMGT | 2.8 | HLA-DRB1*12:02 | 70-87 | IRKLEDLLM | VQSTHIDIRKLEDLLMGT | 2.8 |
| HLA-DRB1*12:02 | 2-16 | ITTLQDYVL | HGEITTLQDYVLDLE | 2.8 | HLA-DRB1*12:02 | 2-16 | ITTLQDYVL | HGEITTLQDYVLDLE | 2.8 | HLA-DRB1*12:02 | 2-16 | ITTLQDYVL | HGEITTLQDYVLDLE | 2.8 |
| HLA-DQA1*01:01/DQB1*05:01 | 6-23 | LQDYVLDLE | TTLQDYVLDLEPEATDLY | 2.9 | HLA-DQA1*01:01/DQB1*05:01 | 6-23 | LQDYVLDLE | TTLQDYVLDLEPEATDLH | 2.9 | HLA-DQA1*01:01/DQB1*05:01 | 6-23 | LQDYVLDLE | TTLQDYVLDLEPEATDLH | 2.9 |
| HLA-DQA1*01:01/DQB1*05:01 | 70-87 | IRKLEDLLM | VQSTHIDIRKLEDLLMGT | 2.9 | HLA-DQA1*01:01/DQB1*05:01 | 70-87 | IRKLEDLLM | VQSTHIDIRKLEDLLMGT | 2.9 | HLA-DQA1*01:01/DQB1*05:01 | 70-87 | IRKLEDLLM | VQSTHIDIRKLEDLLMGT | 2.9 |
| HLA-DQA1*01:01/DQB1*05:01 | 7-20 | LQDYVLDLE | TLQDYVLDLEPEAT | 2.9 | HLA-DQA1*01:01/DQB1*05:01 | 7-20 | LQDYVLDLE | TLQDYVLDLEPEAT | 2.9 | HLA-DQA1*01:01/DQB1*05:01 | 7-20 | LQDYVLDLE | TLQDYVLDLEPEAT | 2.9 |
| HLA-DQA1*01:01/DQB1*05:01 | 71-86 | IRKLEDLLM | QSTHIDIRKLEDLLMG | 3.2 | HLA-DQA1*01:01/DQB1*05:01 | 71-86 | IRKLEDLLM | QSTHIDIRKLEDLLMG | 3.2 | HLA-DQA1*01:01/DQB1*05:01 | 71-86 | IRKLEDLLM | QSTHIDIRKLEDLLMG | 3.2 |
| HLA-DRB1*12:02 | 73-90 | IRKLEDLLM | THIDIRKLEDLLMGTFGI | 3.2 | HLA-DRB1*12:02 | 73-90 | IRKLEDLLM | THIDIRKLEDLLMGTFGI | 3.2 | HLA-DRB1*12:02 | 73-90 | IRKLEDLLM | THIDIRKLEDLLMGTFGI | 3.2 |
| HLA-DQA1*01:01/DQB1*02:01 | 6-21 | YVLDLEPEA | TTLQDYVLDLEPEATD | 3.3 | HLA-DQA1*01:01/DQB1*02:01 | 6-21 | YVLDLEPEA | TTLQDYVLDLEPEATD | 3.3 | HLA-DQA1*01:01/DQB1*02:01 | 6-21 | YVLDLEPEA | TTLQDYVLDLEPEATD | 3.3 |
| HLA-DRB1*12:02 | 1-14 | ITTLQDYVL | MHGEITTLQDYVLD | 3.3 | HLA-DRB1*12:02 | 1-14 | ITTLQDYVL | MHGEITTLQDYVLD | 3.3 | HLA-DRB1*12:02 | 1-14 | ITTLQDYVL | MHGEITTLQDYVLD | 3.3 |
| HLA-DQA1*01:01/DQB1*05:01 | 73-90 | IRKLEDLLM | THIDIRKLEDLLMGTFGI | 3.4 | HLA-DQA1*01:01/DQB1*05:01 | 73-90 | IRKLEDLLM | THIDIRKLEDLLMGTFGI | 3.4 | HLA-DQA1*01:01/DQB1*05:01 | 73-90 | IRKLEDLLM | THIDIRKLEDLLMGTFGI | 3.4 |
| HLA-DQA1*01:01/DQB1*02:01 | 3-18 | ITTLQDYVL | GEITTLQDYVLDLEPE | 3.4 | HLA-DQA1*01:01/DQB1*02:01 | 3-18 | ITTLQDYVL | GEITTLQDYVLDLEPE | 3.4 | HLA-DQA1*01:01/DQB1*02:01 | 3-18 | ITTLQDYVL | GEITTLQDYVLDLEPE | 3.4 |
| HLA-DQA1*01:01/DQB1*02:01 | 73-87 | IRKLEDLLM | THIDIRKLEDLLMGT | 3.4 | HLA-DQA1*01:01/DQB1*02:01 | 73-87 | IRKLEDLLM | THIDIRKLEDLLMGT | 3.4 | HLA-DQA1*01:01/DQB1*02:01 | 73-87 | IRKLEDLLM | THIDIRKLEDLLMGT | 3.4 |
| HLA-DQA1*01:01/DQB1*02:01 | 31-48 | DTIDGPAGQ | SSEEEEDTIDGPAGQAKP | 3.5 | HLA-DQA1*01:01/DQB1*02:01 | 31-48 | DTIDGPAGQ | SSEEEEDTIDGPAGQAKP | 3.5 | HLA-DQA1*01:01/DQB1*02:01 | 31-48 | DTIDGPAGQ | SSEEEEDTIDGPAGQAKP | 3.5 |
| HLA-DQA1*01:01/DQB1*02:01 | 74-85 | IRKLEDLLM | HIDIRKLEDLLM | 3.6 | HLA-DQA1*01:01/DQB1*02:01 | 74-85 | IRKLEDLLM | HIDIRKLEDLLM | 3.6 | HLA-DQA1*01:01/DQB1*02:01 | 74-85 | IRKLEDLLM | HIDIRKLEDLLM | 3.6 |
| HLA-DQA1*01:01/DQB1*05:01 | 7-21 | LQDYVLDLE | TLQDYVLDLEPEATD | 3.6 | HLA-DQA1*01:01/DQB1*05:01 | 7-21 | LQDYVLDLE | TLQDYVLDLEPEATD | 3.6 | HLA-DQA1*01:01/DQB1*05:01 | 7-21 | LQDYVLDLE | TLQDYVLDLEPEATD | 3.6 |
| HLA-DQA1*01:01/DQB1*02:01 | 5-21 | YVLDLEPEA | ITTLQDYVLDLEPEATD | 3.6 | HLA-DQA1*01:01/DQB1*02:01 | 5-21 | YVLDLEPEA | ITTLQDYVLDLEPEATD | 3.6 | HLA-DQA1*01:01/DQB1*02:01 | 5-21 | YVLDLEPEA | ITTLQDYVLDLEPEATD | 3.6 |
| HLA-DRB1*15:02 | 3-14 | ITTLQDYVL | GEITTLQDYVLD | 3.6 | HLA-DRB1*15:02 | 3-14 | ITTLQDYVL | GEITTLQDYVLD | 3.6 | HLA-DRB1*15:02 | 3-14 | ITTLQDYVL | GEITTLQDYVLD | 3.6 |
| HLA-DQA1*01:01/DQB1*05:01 | 76-87 | IRKLEDLLM | DIRKLEDLLMGT | 3.7 | HLA-DQA1*01:01/DQB1*05:01 | 76-87 | IRKLEDLLM | DIRKLEDLLMGT | 3.7 | HLA-DQA1*01:01/DQB1*05:01 | 76-87 | IRKLEDLLM | DIRKLEDLLMGT | 3.7 |
| HLA-DRB1*12:02 | 2-15 | ITTLQDYVL | HGEITTLQDYVLDL | 3.7 | HLA-DRB1*12:02 | 2-15 | ITTLQDYVL | HGEITTLQDYVLDL | 3.7 | HLA-DRB1*12:02 | 2-15 | ITTLQDYVL | HGEITTLQDYVLDL | 3.7 |

Continued 2 Table S5. The HLA-Ⅱ predicted epitopes of HPV-35 E7

| HPV35E7 REF | | | | | HPV35E7 H23Y | | | | | HPV35E7 L28F | | | | |
| --- | --- | --- | --- | --- | --- | --- | --- | --- | --- | --- | --- | --- | --- | --- |
| Allele | Location | Core_peptide | Peptide | Rank | Allele | Location | Core_peptide | Peptide | Rank | Allele | Location | Core_peptide | Peptide | Rank |
| HLA-DRB1*12:02 | 71-86 | IRKLEDLLM | QSTHIDIRKLEDLLMG | 3.7 | HLA-DRB1*12:02 | 71-86 | IRKLEDLLM | QSTHIDIRKLEDLLMG | 3.7 | HLA-DRB1*12:02 | 71-86 | IRKLEDLLM | QSTHIDIRKLEDLLMG | 3.7 |
| HLA-DRB1*12:02 | 74-90 | IRKLEDLLM | HIDIRKLEDLLMGTFGI | 3.7 | HLA-DRB1*12:02 | 74-90 | IRKLEDLLM | HIDIRKLEDLLMGTFGI | 3.7 | HLA-DRB1*12:02 | 74-90 | IRKLEDLLM | HIDIRKLEDLLMGTFGI | 3.7 |
| HLA-DRB1*12:02 | 73-85 | IRKLEDLLM | THIDIRKLEDLLM | 3.8 | HLA-DRB1*12:02 | 73-85 | IRKLEDLLM | THIDIRKLEDLLM | 3.8 | HLA-DRB1*12:02 | 73-85 | IRKLEDLLM | THIDIRKLEDLLM | 3.8 |
| HLA-DQA1*01:01/DQB1*02:01 | 32-49 | DTIDGPAGQ | SEEEEDTIDGPAGQAKPD | 3.9 | HLA-DQA1*01:01/DQB1*02:01 | 32-49 | DTIDGPAGQ | SEEEEDTIDGPAGQAKPD | 3.9 | HLA-DQA1*01:01/DQB1*02:01 | 32-49 | DTIDGPAGQ | SEEEEDTIDGPAGQAKPD | 3.9 |
| HLA-DQA1*01:01/DQB1*02:01 | 75-87 | IRKLEDLLM | IDIRKLEDLLMGT | 4 | HLA-DQA1*01:01/DQB1*02:01 | 75-87 | IRKLEDLLM | IDIRKLEDLLMGT | 4 | HLA-DQA1*01:01/DQB1*02:01 | 75-87 | IRKLEDLLM | IDIRKLEDLLMGT | 4 |
| HLA-DRB1*12:02 | 1-16 | ITTLQDYVL | MHGEITTLQDYVLDLE | 4 | HLA-DRB1*12:02 | 1-16 | ITTLQDYVL | MHGEITTLQDYVLDLE | 4 | HLA-DRB1*12:02 | 1-16 | ITTLQDYVL | MHGEITTLQDYVLDLE | 4 |
| HLA-DQA1*01:01/DQB1*05:01 | 74-90 | IRKLEDLLM | HIDIRKLEDLLMGTFGI | 4.1 | HLA-DQA1*01:01/DQB1*05:01 | 74-90 | IRKLEDLLM | HIDIRKLEDLLMGTFGI | 4.1 | HLA-DQA1*01:01/DQB1*05:01 | 74-90 | IRKLEDLLM | HIDIRKLEDLLMGTFGI | 4.1 |
| HLA-DRB1*15:02 | 2-14 | ITTLQDYVL | HGEITTLQDYVLD | 4.2 | HLA-DRB1*15:02 | 2-14 | ITTLQDYVL | HGEITTLQDYVLD | 4.2 | HLA-DRB1*15:02 | 2-14 | ITTLQDYVL | HGEITTLQDYVLD | 4.2 |
| HLA-DQA1*01:01/DQB1*02:01 | 3-19 | ITTLQDYVL | GEITTLQDYVLDLEPEA | 4.3 | HLA-DQA1*01:01/DQB1*02:01 | 3-19 | ITTLQDYVL | GEITTLQDYVLDLEPEA | 4.3 | HLA-DQA1*01:01/DQB1*02:01 | 3-19 | ITTLQDYVL | GEITTLQDYVLDLEPEA | 4.3 |
| HLA-DQA1*01:01/DQB1*02:01 | 73-86 | IRKLEDLLM | THIDIRKLEDLLMG | 4.3 | HLA-DQA1*01:01/DQB1*02:01 | 73-86 | IRKLEDLLM | THIDIRKLEDLLMG | 4.3 | HLA-DQA1*01:01/DQB1*02:01 | 73-86 | IRKLEDLLM | THIDIRKLEDLLMG | 4.3 |
| HLA-DRB1*12:02 | 2-13 | ITTLQDYVL | HGEITTLQDYVL | 4.3 | HLA-DRB1*12:02 | 2-13 | ITTLQDYVL | HGEITTLQDYVL | 4.3 | HLA-DRB1*12:02 | 2-13 | ITTLQDYVL | HGEITTLQDYVL | 4.3 |
| HLA-DQA1*01:01/DQB1*02:01 | 30-47 | DTIDGPAGQ | DSSEEEEDTIDGPAGQAK | 4.5 | HLA-DQA1*01:01/DQB1*02:01 | 30-47 | DTIDGPAGQ | DSSEEEEDTIDGPAGQAK | 4.5 | HLA-DQA1*01:01/DQB1*02:01 | 30-47 | DTIDGPAGQ | DSSEEEEDTIDGPAGQAK | 4.5 |
| HLA-DQA1*01:01/DQB1*02:01 | 4-21 | LQDYVLDLE | EITTLQDYVLDLEPEATD | 4.5 | HLA-DQA1*01:01/DQB1*02:01 | 4-21 | LQDYVLDLE | EITTLQDYVLDLEPEATD | 4.5 | HLA-DQA1*01:01/DQB1*02:01 | 4-21 | LQDYVLDLE | EITTLQDYVLDLEPEATD | 4.5 |
| HLA-DQA1*01:01/DQB1*02:01 | 74-88 | IRKLEDLLM | HIDIRKLEDLLMGTF | 4.5 | HLA-DQA1*01:01/DQB1*02:01 | 74-88 | IRKLEDLLM | HIDIRKLEDLLMGTF | 4.5 | HLA-DQA1*01:01/DQB1*02:01 | 74-88 | IRKLEDLLM | HIDIRKLEDLLMGTF | 4.5 |
| HLA-DQA1*01:01/DQB1*02:01 | 3-20 | LQDYVLDLE | GEITTLQDYVLDLEPEAT | 4.7 | HLA-DQA1*01:01/DQB1*02:01 | 3-20 | LQDYVLDLE | GEITTLQDYVLDLEPEAT | 4.7 | HLA-DQA1*01:01/DQB1*02:01 | 3-20 | LQDYVLDLE | GEITTLQDYVLDLEPEAT | 4.7 |
| HLA-DRB1*12:02 | 76-88 | IRKLEDLLM | DIRKLEDLLMGTF | 4.7 | HLA-DRB1*12:02 | 76-88 | IRKLEDLLM | DIRKLEDLLMGTF | 4.7 | HLA-DRB1*12:02 | 76-88 | IRKLEDLLM | DIRKLEDLLMGTF | 4.7 |
| HLA-DQA1*01:01/DQB1*05:01 | 70-86 | IRKLEDLLM | VQSTHIDIRKLEDLLMG | 4.9 | HLA-DQA1*01:01/DQB1*05:01 | 70-86 | IRKLEDLLM | VQSTHIDIRKLEDLLMG | 4.9 | HLA-DQA1*01:01/DQB1*05:01 | 70-86 | IRKLEDLLM | VQSTHIDIRKLEDLLMG | 4.9 |
| HLA-DQA1*01:01/DQB1*05:01 | 72-85 | IRKLEDLLM | STHIDIRKLEDLLM | 4.9 | HLA-DQA1*01:01/DQB1*05:01 | 72-85 | IRKLEDLLM | STHIDIRKLEDLLM | 4.9 | HLA-DQA1*01:01/DQB1*05:01 | 72-85 | IRKLEDLLM | STHIDIRKLEDLLM | 4.9 |
| HLA-DQA1*01:01/DQB1*02:01 | 8-20 | YVLDLEPEA | LQDYVLDLEPEAT | 4.9 | HLA-DQA1*01:01/DQB1*02:01 | 8-20 | YVLDLEPEA | LQDYVLDLEPEAT | 4.9 | HLA-DQA1*01:01/DQB1*02:01 | 8-20 | YVLDLEPEA | LQDYVLDLEPEAT | 4.9 |
| HLA-DRB1*12:02 | 1-15 | ITTLQDYVL | MHGEITTLQDYVLDL | 4.9 | HLA-DRB1*12:02 | 1-15 | ITTLQDYVL | MHGEITTLQDYVLDL | 4.9 | HLA-DRB1*12:02 | 1-15 | ITTLQDYVL | MHGEITTLQDYVLDL | 4.9 |
| HLA-DQA1*01:01/DQB1*02:01 | 32-48 | DTIDGPAGQ | SEEEEDTIDGPAGQAKP | 5 | HLA-DQA1*01:01/DQB1*02:01 | 32-48 | DTIDGPAGQ | SEEEEDTIDGPAGQAKP | 5 | HLA-DQA1*01:01/DQB1*02:01 | 32-48 | DTIDGPAGQ | SEEEEDTIDGPAGQAKP | 5 |
| HLA-DRB1*12:02 | 74-91 | IRKLEDLLM | HIDIRKLEDLLMGTFGIV | 5 | HLA-DRB1*12:02 | 74-91 | IRKLEDLLM | HIDIRKLEDLLMGTFGIV | 5 | HLA-DRB1*12:02 | 74-91 | IRKLEDLLM | HIDIRKLEDLLMGTFGIV | 5 |
